# Solid–Liquid Interfacial Free Energy from Computer Simulations: Challenges and Recent Advances

**DOI:** 10.1021/acs.chemrev.4c00833

**Published:** 2025-05-12

**Authors:** Nicodemo Di Pasquale, Jesús Algaba, Pablo Montero de Hijes, Ignacio Sanchez-Burgos, Andres R. Tejedor, Stephen R. Yeandel, Felipe J. Blas, Ruslan L. Davidchack, Jorge R. Espinosa, Colin L. Freeman, John H. Harding, Brian B. Laird, Eduardo Sanz, Carlos Vega, Lorenzo Rovigatti

**Affiliations:** † Department of Industrial Chemistry “T. Montanari”, 9296 Università di Bologna, via Gobetti 85, 40129 Bologna, Italy; ‡ Laboratorio de Simulación Molecular y Química Computacional, CIQSO-Centro de Investigación en Química Sostenible and Departamento de Ciencias Integradas, 16743 Universidad de Huelva, 21006 Huelva, Spain; § Faculty of Physics, 27258 University of Vienna, A-1090 Vienna, Austria; ∥ Yusuf Hamied Department of Chemistry, 2152 University of Cambridge, Lensfield Road, Cambridge CB2 1EW, United Kingdom; ⊥ Department of Physical Chemistry, 16734 Complutense University of Madrid, Avenida Complutense, Madrid 28040, Spain; # Department of Materials Science and Engineering, 7315 University of Sheffield, Sheffield S1 3JD, United Kingdom; 7 School of Computing and Mathematical Sciences, 4488 University of Leicester, Leicester LE1 7RH, United Kingdom; 8 Department of Chemistry, 4202 University of Kansas, Lawrence, Kansas 66045, United States; 9 Physics Department, 9311 Sapienza University of Rome, P.le A. Moro 5, 00185 Rome, Italy; 10 Maxwell Centre, Cavendish Laboratory, Department of Physics, 2152 University of Cambridge, J J Thomson Avenue, Cambridge CB3 0HE, United Kingdom

## Abstract

The study of interfacial properties in liquid–liquid and liquid–vapor systems has a history of nearly 200 years, with significant contributions from scientific luminaries such as Thomas Young and Willard Gibbs. However, a similar level of understanding of solid–liquid interfaces has emerged only more recently, largely because of the numerous complications associated with the thermodynamics needed to describe them. The accurate calculation of the interfacial free energy of solid–liquid systems is central to determining which interfaces will be observed and their properties. However, designing and analyzing the molecular dynamics simulations required to do this remains challenging, unlike the liquid–liquid or liquid–vapor cases, because of the unique complications associated with solid–liquid systems. Specifically, the lattice structure of solids introduces spatial directionality, and atomic configurations in solids can be altered by stretching. The primary aim of this review is to provide an overview of the numerical approaches developed to address the challenge of calculating the interfacial free energy in solid–liquid systems. These approaches are classified as (i) direct methods, which compute interfacial free energies explicitly, albeit often through convoluted procedures, and (ii) indirect methods, which derive these free energies as secondary results obtained from the analysis of simulations of an idealized experimental configuration. We also discuss two key topics related to the calculation of the interfacial free energy of solid–liquid systems: nucleation theory and curved interfaces, which represent important problems where research remains highly active.

## Graphical abstract

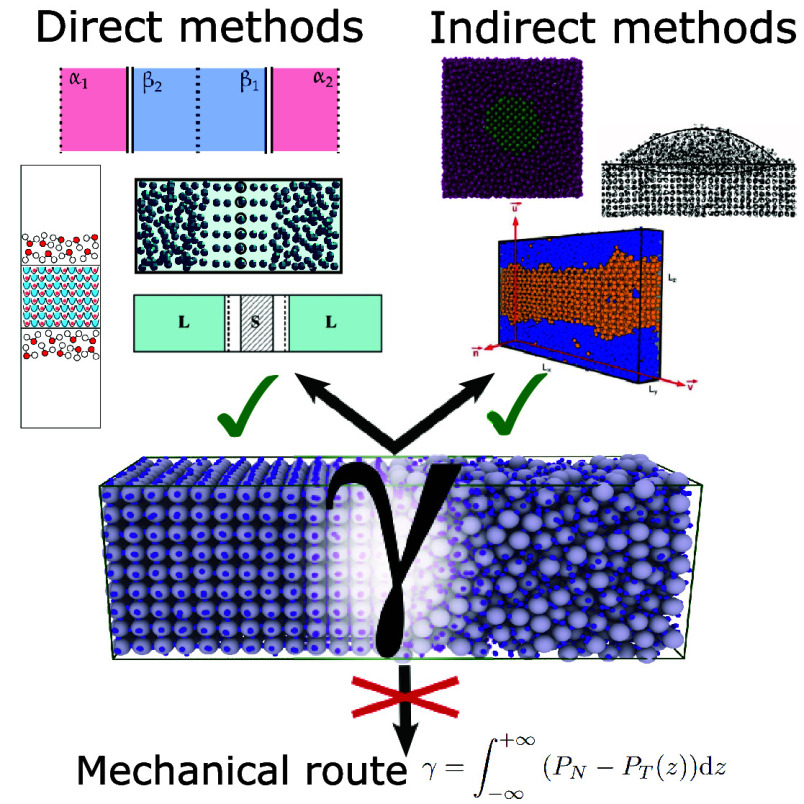

## 1 Introduction

Wolfgang Pauli once famously remarked, “God made the bulk; surfaces were invented by the devil”, vividly illustrating the notion that surfaces are realms of chaos and darkness, in contrast to the order and rationality of the bulk.^1^ He would, no doubt, have been happy to include interfaces as examples of diabolic malice. Whereas interfaces can be challenging to deal with, a topic we will explore in this review, they are often more fascinating than the bulk, as many interesting and intriguing phenomena arise exclusively at the interface between two or more phases. This is especially true for solid–liquid interfaces. Detailed knowledge of the structure and thermodynamic properties of the interfaces that are formed when a solid and other coexisting phases meet is the basis for many physical phenomena and technological processes, making them a matter of primary interest in several different fields. We dedicate the first part of this review to describing these problems and applications. This will show why so much effort has been required to develop reliable methods for the accurate determination of the structures, energetics, and properties of solid–liquid interfaces. The rest of the review will discuss the methods themselves, along with their advantages and disadvantages, and will indicate where further work is needed. A central challenge (and the theme of this review) is the accurate calculation of the interfacial free energy (IFE).

The formation of a new solid phase from a liquid involves two different but related processes. In *freezing* (or *solidification*) we have the formation of a new solid phase from its melt; in *crystallization* the new solid phase is precipitated from a solution in which the solid is dissolved. These two processes are related by the fact that the physical process allowing the formation of the new phase must involve the creation of a solid–liquid interface. This process is frequently *nucleation*,^2–5^ which we will discuss in detail later. In the following paragraphs, we give a few examples to illustrate the importance of solid–liquid interfaces.

One of the most studied systems undergoing freezing is water,^6–10^ not only because of its theoretical importance but also because of its wide range of applications. For example, ice formation plays a crucial role both in atmospheric science (in the accurate representation of the climate^11,12^ ) and in the design of functional materials with anti-icing properties.^13,14^ Such materials have many applications, ranging from increasing safety in aviation,^15^ where ice formation on wings is one of the main safety concerns, to increasing the performance of wind turbines in cold climates^16,17^ and reducing damage to overhead power lines.^18,19^

When metallic materials solidify,^20^ the solid–liquid IFE controls the formation of microstructures on which, in turn, the quality of the final product in casting depends.^21^ In particular, the dendritic growth velocity depends on the orientation of the crystal lattice with respect to the solid–liquid interface.^22,23^ This will be discussed in more detail later. It is important in several systems, e.g., the Al–Cu alloy^24,25^ and the solid–liquid coexistence curve in Ni, Cu, Al,^26^ and Ti.^27^ A different arrangement of the atoms in the solid will result in a different structure of the liquid layers close to the solid,^28–31^ making the IFE dependent on the orientation of the planes of the crystal structure with respect to the solid–liquid interface. This highlights one of the difficulties that concern solid interfaces in general (not only in the context of solidification), which we will discuss in the next section. When solids are involved, the IFE is not a single scalar quantity but assumes different values depending on the structure of the solid–liquid interface.

In the process of the formation of a new solid phase from a solution, the role of the IFE between the solid and liquid phases in the nucleation process is well established^32^ in systems as diverse as polymers^33,34^ and biominerals.^35^ The IFE can control not only the orientation of the crystal structure of the new solid phase but also which polymorph will be formed if the solid exhibits polymorphism.^36,37^ Predicting which polymorph can be formed is important not only for biological processes^35^ but also in the crystallization of pharmaceutical products where the formation of the right polymorphs of drugs is essential for their effectiveness and safety.^38,39^ The behavior of such industrial products (including both excipients and active pharmaceutical ingredients) with respect to binder–drug adhesion,^40^ granulation performance,^41–43^ the flow of powders, and compaction^44,45^ can be related to their interfacial properties.^46–49^

An application that has become extremely important in recent times, in which interfacial properties play a pivotal role, is the design of next-generation graphene-based energy storage devices, such as electrochemical double layer (super)­capacitors (EDLCs).^50,51^ Energy storage devices such as lithium-ion batteries, despite their good performance in terms of energy density (up to 180 Wh kg^–1^), have their downsides, including a slow power delivery or uptake.^50^ Therefore, new materials are being studied to overcome these limitations. Despite their lower energy density (about 5 Wh kg^–1^), graphene-based devices (using graphene,^52–55^ porous activated carbon,^56^ or carbon nanotubes^57,58^ ) have higher charge storage capacities, favorable specific energy-to-power ratios (owing to rapid charge–discharge cycling^53^ controlled by changes of an applied potential) and lifetimes that can reach millions of cycles.^56^ Such characteristics make graphene-based energy storage devices appealing as a solution to the problems of low charge capacities and slow charge/discharge rates found in conventional batteries.^59^ Energy storage in graphene-based supercapacitors is based on the reversible non-Faradaic physisorption of ions in the electrical double layer.^52^ With its high surface area, graphene can, in principle, guarantee a higher capacitance than amorphous carbon-based electrodes. The area offered by the electrode, however, is not the only parameter that enters the quantification of the capacitance. In order to be successful, the electrochemically active surface area of the electrode should be easily accessible to the electrolyte. This, in turn, depends on the ability of the electrolyte to wet the electrode surface, which represents another manifestation of the solid–liquid IFE. In addition, the ability of the electrolyte to wet the graphene surface changes as a function of the potential applied to the electrode, a phenomenon known as “electro-wetting”.^60^ For these reasons, the detailed simulation of graphene–electrolyte interfaces using molecular models has become an essential tool to understand them. Because of the scale of most systems, classical molecular dynamics (MD) is the preferred simulation tool (see, e.g., refs 61–63 and references therein). Two different setups are used: constant charge and constant (electrical) potential (see refs 64–67 for a discussion of these different methodologies). However, to capture the behavior of graphene in contact with electrolytes, a more detailed account of the electrostatic interactions is needed.^68^ The development of such improved descriptions of the interactions between electrode and electrolyte has been addressed using either quantum mechanical/molecular dynamics models (see, e.g., refs 69–71 ), polarizable force-fields,^72–74^ or force-fields based on machine learning^75–77^ (although ref 77 is specific for metal electrodes). The combination of such advanced descriptions with the methodologies presented here will surely be at the forefront of the investigation of such systems. For a more detailed discussion of the simulation and characterization of electrode–electrolyte interfaces, we refer the interested reader to the following reviews (and references therein).^78–80^

Another area where knowledge of interfacial properties is essential is thermal transport across solid–liquid interfaces, in particular when the size of the system considered is microscopic and, therefore, the interface/volume ratio of the system involved becomes large.^81^ The control of thermal transport at the nanoscale is important for medical applications, water purification, and microelectronics^82^ (see ref 83 and the references therein for a more detailed account of the different applications). The interfacial thermal conductance is related to the affinity between the solid and the liquid at the interface: the stronger the attraction between the two phases, the lower the thermal resistance.^83^ This effect was observed for the first time by Kapitza^84^ and is now known as “Kapitza resistance” (although it refers to conductance rather than resistance). A measure of the strength of this attraction is the wettability of the solid interface by the liquid, and previous work shows that there is a direct relationship between wettability and thermal conductance.^85,86^ In turn, as we discussed in the case of electrochemical devices, we can consider the wettability as just another manifestation of the IFE, and the ability to obtain a reliable value of this property in a variety of systems becomes vital.

Until now, we have talked about “surfaces” and “interfaces” without providing a proper definition, appealing instead to common usage. From now on, we will define these concepts more rigorously and put them into the context of thermodynamic theory for quantitative analysis and discussion. Indeed, the analysis of the properties of interfaces is deeply rooted in thermodynamics, as shown in the pioneering work of J. W. Gibbs, one of the earliest contributors to this topic and also one of the founders of modern thermodynamics. In his work,^87^ he defined the interface between two different phases as a zero-width plane (later called the “Gibbs dividing plane”), to which he ascribed the excess of the thermodynamic quantities that characterize the presence of an interface between two phases. One of these quantities is the IFE, γ, which is defined as the reversible work required to create a unit area of the interface under the coexistence conditions for the two phases. We introduce here some of the notation that will be used throughout the rest of the review by explicitly stating which two phases are in contact through the interface. Throughout the review we will indicate the solid–liquid IFE using γ_*sl*_. However, there will be some occasions in which we must distinguish between the solid in contact with its own melt and the solid forming some chemically heterogeneous interface with the liquid (e.g., in crystallization). We will use γ_*sm*_ for the former case and γ_*sx*_ for the latter (we refer to the Nomenclature Section for the description of all the symbols).

The Gibbs approach was later generalized by Cahn, with a formulation avoiding the need to locate the position of the dividing surface.^88^ As we shall discuss in the following sections, the definition of the IFE, while straightforward for liquid–liquid systems, involves some subtleties when the system contains a solid interface. Two basic differences between liquid–liquid and solid–liquid interfaces require that the strategy needed to calculate the IFE using MD simulations must be very different for the two types of systems. Whereas in liquid–liquid systems it is possible to use rather simple relations to calculate the interfacial properties using MD simulations (such as using the stress within the system as a proxy for the energy required to create the interface), such shortcuts are not possible for solid–liquid interfaces because a crystal structure can support a noncompressive stress. Moreover, the directionality of the crystal lattice implies a dependence of the IFE on the orientation of the solid surface in contact with the liquid. More complicated calculations are therefore needed, as one often has to resort to using very basic thermodynamic relations. The energy needed to create a solid–liquid interface must be calculated by creating a new interface in a simulation box, an operation that is much more complicated than just calculating the force acting between atoms in the system, with the additional burden that each of these complex calculations must be repeated for each independent orientation of the crystal in contact with the liquid phase.

This review is organized as follows: we will first introduce the interface-specific quantities in solid–liquid systems starting from their thermodynamic definitions. We provide a brief account of the reasons why solid–liquid interfaces differ from liquid–liquid ones. From this we will move on to the presentation of the different methods devised in the literature to obtain the IFE for solid–liquid systems. Here, we partition the different techniques into two main groups, which we label “indirect” and “direct” methods, based on the way that IFEs are computed. This is the most extensive part of the review, and is complemented by appendix B, which gives a critical discussion of the main direct methods presented. Although such methodologies have been applied to several different systems, there are some systems that can be considered as “benchmarks” against which the results of any new extension of existing methodologies or the development of new ones should be tested: the hard-sphere and Lennard-Jones models. For this reason, we include a detailed description of these systems, along with a detailed comparison of the results using the different approaches. We then discuss in some detail the calculation of interfacial properties for more realistic systems, namely water and hydrates. We selected these systems both for their importance and also because they exemplify the kind of complications one has to face when considering more realistic systems. We then explain the importance of determining IFEs in the theory of nucleation and devote a section to curved interfaces. These last two sections (sections 8 and 9) should be considered “open”, in the sense that, despite being long-known problems (the first appearance of the problem of curved interfaces is in the work of Young in 1805,^89^ and the theory of nucleation is now a century old^90,91^ ), they still present significant challenges and questions. In the last part, we draw some conclusions and give some perspective on future applications and ideas related to the methodologies described here.

## 2 Challenges in Characterizing the Physics of Solid–Liquid Interfaces

Whereas the IFEs for liquid–vapor and liquid–liquid interfaces are well-known quantities, characterized both theoretically^92,93^ and experimentally,^94,95^ this is not the case for solid–liquid interfaces. One of the most important reasons for this difference comes from the fact that, unlike liquid–vapor and liquid–liquid interfaces, the IFE of interfaces involving solids depends on the orientation of the interface with respect to the solid lattice.^96,97^ In particular, the IFE for a given solid–liquid system has different values of γ_*sl*_ for different orientations of the crystal lattice that are not related by symmetry (for example, see the results reported in refs 96 and 97).

The calculation of the IFEs associated with solid–liquid interfaces is extremely challenging for both simulation and experiment. Although we refer to the literature for more detailed accounts (see ref 98 for simulation methodologies and ref 99 for experimental techniques), we will highlight some of the major difficulties in the experimental determination of the IFE for solid–liquid interfaces. The reader is warned that in some of the early work (both simulations and experiments) there is ambiguity about what is being compared to what. The common assumption that the entropic contribution to the IFE is negligible means that frequent reference was made to “surface energies” or “interfacial energies”, without clarifying whether those quantities were free energies, enthalpies, or configurational (potential) energies per unit area (see, e.g., Tables 4 and 5 in ref 100).

One of the most widely used approaches to determine the solid–liquid IFE is based on Young’s equation^89^ and consists of measuring the angle that a liquid droplet makes with respect to a solid surface with which it is in contact.^101^ Although the idea itself is relatively straightforward, the measurement of the droplet angle is plagued by several issues, either kinetic (evaporation, vapor adsorption, swelling) or thermodynamic (because the surface on which the droplet is located has to be flat and chemically homogeneous down to the molecular scale, and gravity must not disturb the solid–liquid–vapor system). If these conditions are not met, the departure from ideality generates hysteresis between the direct process, wetting, and its inverse, i.e., surface dewetting.^102^ This hysteresis results in non-unique measurements of the contact angle, making Young’s equation inapplicable for the calculation of the solid–liquid IFE.^103^ Special care must be taken to minimize this effect during experiments (see e.g., ref 104). Moreover, new methodologies are badly needed to deal with rough surfaces.^105^

Another common way to determine IFEs is through the use of measurements of crystal nucleation rates in supercooled fluids, from which the IFE can be determined using classical nucleation theory (CNT)^106–109^ (see also section 8). This can be challenging: nuclei can be hard to identify even when the approximations of CNT are valid. As an example of the difficulty of measuring solid–liquid IFEs, we highlight the case of the ice–water interface, for which there is still no consensus on its experimental value^109^ despite the importance of such a system.

Another method of evaluating the IFE involves the measurement of the groove morphology at the intersections between a solid–liquid interfacial boundary and grain boundaries in the solid phase.^110^ For the archetypal case of the interface between hard-sphere solid and its melt, the IFE was inferred from nucleation measurements using the CNT framework^111^ and later compared to the equilibrium crystal-fluid IFE directly obtained from the analysis of the groove morphology under coexistence conditions.^112^ The observed values ranged from 0.51 to 0.66 *k*_B_ *T*,^113^ which agrees reasonably well with simulations and theory^96,114–116^ and shows a moderate systematic dependence on the degree of metastability. However, this technique usually requires additional alloying elements in the liquid phase to mark the interface position, which has a significant influence on the measurement result. Moreover, this technique cannot resolve the variation of γ­(**n̂**) as a function of the orientation of the crystal lattice with respect to the plane of the solid–liquid interface (defined by the unit normal **n̂**). The development of new techniques to determine solid–liquid IFEs is an active area of research, with newly proposed techniques to measure γ_*sl*_, such as Sessile Drop Accelerometry.^104^ The challenges and approximations required on the experimental side to determine the solid–liquid IFE increase the value of numerical methods for determining this quantity from molecular simulations, as shown by the amount of research that has been dedicated to the development of the methodologies discussed in the rest of this review.

## 3 Solid–Liquid Interfaces: From Macro to Micro and Back

While the main objective of this review is to provide an overview of the computational models developed to determine the solid–liquid IFE, we must first answer a question readers might ask: “why are there so many methods and why are they so diverse?” In order to answer this question, we must take a step back and discuss the thermodynamics of solid–liquid interfaces. This will be the main topic of section 3.1. However, since in this review we want to focus on computational methods, we will limit the discussion to the main ideas and concepts, leaving interested readers to consult the references for the details.

In section 3.2 we will connect the thermodynamics of interfaces to statistical mechanics by describing one of the first ways, proposed by Kirkwood and Buff, to calculate the IFE using knowledge of the interactions in an atomistic system (now a standard tool in molecular calculations). The Kirkwood–Buff method was developed for fluid–fluid interfaces, but it fails to give a proper account of solid–liquid interfacial energies. This failure arises from the fact that a solid phase not only possesses an internal orientation but also can be stretched. Neither consideration applies to a liquid. This motivates the discussion given in section 3.3 where we have to go back to first principles, to the thermodynamic definition of the IFE in a solid–liquid system as a free energy. This is the underlying reason for the number and types of molecular models proposed to determine IFEs. Calculating a free energy is a much more complicated problem than using the Kirkwood–Buff method.

### 3.1 Thermodynamics of Interfaces

Before starting the analysis of the thermodynamic properties of interfaces, we will give a brief discussion of terminology. The Helmholtz free energy is represented by *F*, and the Gibbs free energy is represented by *G*. Here and throughout the review we use A to represent the *total* interfacial area: if the system contains two interfaces, as in a slab, then A is twice the area of a single interface. We use the symbol *f*_*ij*_ to indicate the interfacial stress, where *i* and *j* refer to two Cartesian axes (usually the interfacial lattice vectors) in the plane of the interface. As noted before,^117^ it is better to avoid using the term “surface tension” for the quantity γ: it is harmless for the case of a (single-component) liquid, but it can be a source of confusion when a solid phase is involved.

Although the particular properties of interfaces have been known since the times of Young and Laplace, Gibbs was the first to offer a detailed and quantitative analysis of such systems in his monumental work *On the Equilibrium of Heterogeneous Substances*.^87^ When two masses of materials are put in contact, the total energy, the total entropy, and other extensive quantities are not simple algebraic sums of terms referring to each coexisting system considered without any interface. More formally, let us consider a solid system (indicated with the subscript *s*) in contact with a liquid system (indicated with a subscript *l*) and call the energy of the new composite system *E*. We use the symbol *E* for the internal energy of a system instead of the more common symbol *U*, since the latter will be used to denote the configurational (potential) energy in Molecular Dynamics simulations. Let *E*_*s*_ be the energy of the solid (sub)­system when it is not in contact with the liquid (that is, in Gibbs’ words, without a *surface of discontinuities*), and let *E*_*l*_ have an analogous meaning for the liquid subsystem. Then, Gibbs reasoned

$$E\neq E_{s}+E_{l}$$ 1

The difference is due to the presence of the interface between the two subsystems: if the interface changes, so does the energy of the composite system. This is different from any process involving other extensive quantities (such as mass). From eq 1 we obtain the definition of *interface excess* quantities as

$$E^{XS}=E-E_{s}-E_{l}$$ 2

where we use the superscript notation ^*XS*^ to indicate an excess property of the interface. Similarly, we can define an excess entropy *S* ^*XS*^ and an excess number of atoms at the interface *N* ^*XS*^ (see, e.g., ref 118). The difference between the extensive quantities in a system with and without an interface is therefore taken into account by the excess interface quantities. In our discussion of the thermodynamics of the interface, and also in later sections (e.g., the Gibbs–Cahn model discussed in section 5.7) we will use excess quantities per unit area of the interface. Following Cahn,^119^ we will define an excess thermodynamic (extensive) quantity per unit area of interface using brackets, as $\left[E\right]=E^{XS}/A$ for internal energy, $\left[S\right]=S^{XS}/A$ for entropy, $\left[N_{k}\right]=N_{k}^{XS}/A$ for the amount of chemical species *k*, $\left[V\right]=V^{XS}/A$ for the excess volume, and analogously for the other (extensive) thermodynamic quantities.

In the work of Gibbs, excess quantities are reported as relative to an interface of area A with a different notation from the one used here but which we include for completeness, as it can be useful when reading older work: energy $e=E^{XS}/A=\left[E\right]$ , entropy $\eta =S^{XS}/A=\left[S\right]$ , and $\Gamma_{k}=N_{k}^{XS}/A=\left[N_{k}\right]$ for the amount of chemical species *k*.

In Gibbs’ original treatment of the interface, excess quantities were assigned to a geometrical interface that separates the two subsystems, with each considered as if it was not in contact with another different phase.^87^ An alternative way of assigning these excess quantities was devised by Guggenheim.^120^ The equivalence of these two approaches was shown by Cahn^88^ and will be briefly discussed in section 5.7, which is dedicated to Cahn’s thermodynamic model of interfaces. Of the two, the latter is more general, as it includes the case where an excess of volume is assigned to the interfacial region, which cannot be described using the definition of an interface of separation as a two-dimensional surface. From the definition of excess properties, for a *c*-component system, the (specific) interface free energy is usually defined through the so-called *fundamental interface thermodynamic equation*:^121–123^

$$\gamma =\left[F\right]-\overset{c}{\underset{k=1}{\sum }}\mu_{k}\left[N_{k}\right]$$ 3

Equation 3 shows an important terminology problem with the different quantities used to describe an interface. The quantity γ is equal to the Helmholtz interfacial free energy [*F*] only when [*N*_*k*_] = 0 for all *k*, which is true for a one-component system but not necessarily true for multicomponent mixtures.^121^ Despite being usually reported as the definition of γ, eq 3 is not the most general equation describing the relation among thermodynamic quantities since it assumes, consistent with Gibbs’ formalism, a zero excess volume at the interface. The Cahn derivation, however (see eq 7 in ref 88), does not consider a two-dimensional interface of discontinuity but instead considers a three-dimensional layer that includes the interface. In this case, the following equation (reported also in ref 124) for γ should be used:

$$\gamma =\left[E\right]-T\left[S\right]+P\left[V\right]-\overset{c}{\underset{k=1}{\sum }}\mu_{k}\left[N_{k}\right]=\left[G\right]-\overset{c}{\underset{k=1}{\sum }}\mu_{k}\left[N_{k}\right]$$ 4

Using Cahn’s formalism, we can show that eq 3 is a special case of eq 4 when the excess volume is zero (see section 5.7).

The situation becomes even more complicated when we introduce the concept of *interfacial stress*, *f*_*ij*_. Although an accurate definition of these quantities is crucial for consistency in their analysis and description, the debate about their appropriate names, which began long ago,^125,126^ has not yet been resolved. Unfortunately, this problem has been overlooked in the past, since for a single-component liquid–liquid system γ = *f*_*ij*_ = [*F*][Bibr ref126] and therefore there is no need to distinguish between the three quantities [We are committing an abuse of notation here. For a liquid–liquid interface, the interfacial stress tensor is isotropic and diagonal. Therefore, it can be described by a single term *f*, which is the one to be equated with γ. The equality γ = [*F*] can be obtained from eq 3, with *k* = 1. In this case it can be shown^127^ that a Gibbs dividing surface can be chosen such that the excess component at the interface is equal to zero. The equality γ = *f*_*ij*_ for a liquid–liquid interface is then a consequence of the Shuttleworth equation (which will be introduced with eq 6)]. However, when considering more general systems (for example, multicomponent solid–liquid interfaces) the identification of γ, *f*_*ij*_, and [*F*] is no longer true (see e.g., ref 127), and the different quantities must be kept distinct by using a consistent nomenclature. We emphasize this point because it is crucial for understanding the rest of the review.

The chain of equalities γ = *f*_*ij*_ = [*F*] implies two issues that make the determination of the IFE for a solid–liquid system with arbitrary composition much more complicated than for the single-component liquid–liquid system:

1. γ = *f*_*ij*_ is no longer true for solid–liquid interfaces. This explains why the determination of IFEs for solid–liquid systems is more complicated than that for liquid–liquid ones.
2. γ = [*F*] is no longer true for multicomponent systems. This point is not specific to solid–liquid interfaces and will not be the main focus of this review. Nevertheless, it introduces further complexity into the simulations to determine the solid–liquid IFE and therefore merits a mention here.

Using eq 3 and the analysis in ref 128, we can work out the implications of such definitions for solid interfaces. In eq 3, γ is defined as the interfacial excess of the grand potential (also known as the Kramer potential^123^ or the Landau potential). The grand potential contains the term related to mechanical work acting on a system, which for a bulk phase α is $\Psi^{\alpha }=-P\left[V^{\alpha }\right]$ , whereas in the presence of discontinuities due to the interface it also takes into account the contribution of the IFE: Ψ = *F* – ∑_*k*_μ_*k*_ *N*_*k*_, i.e., γ ≔ [Ψ].

In a system with an interface, for any reversible transformation at constant *T* and μ generated by the action of mechanical forces, the work on the system is equal to the work done on the bulk phase plus interface work *W* ^*XS*^:^128^

$$W^{XS}=\Delta \int_{A}\gamma ds,\left(T,\mu \mathrm{const}.\right)$$ 5

where $\Delta \int_{A}\gamma ds$ represents the difference between the integral calculated with a system of area A+ΔA and with a system of area A . The previous equation implies that, in a transformation in which the total volume of the bulk phases α and β is kept constant, ΔΨ = *W* ^*XS*^, and therefore for a liquid–liquid interface the IFE is determined uniquely by *T* and μ. As a result, from eq 5 we obtain again the result that the interfacial work is equal to γΔA , where ΔA is the change in the interfacial area. However, for an interface involving a solid, the IFE may depend on the particular crystallographic orientation of the interface (as in the results reported in ref 129 for a modified Lennard-Jones system) or the state of strain of the crystal (as in the results reported in ref 117 for a Lennard-Jones system). In the latter case, the more general expression eq 5 must be considered. Gibbs was the first to point out that while the state of tension within liquids caused by surface-tension-related forces can be directly linked to the work required to create the interface, no such simple relation exists when solid phases are considered. Indeed, in this case the work needed to create a new surface and the work involved in stretching it may be different and must be distinguished. From this observation we therefore obtain two quantities related to an interface:

1. The IFE, the reversible work needed to create a new unit of interface in a system without an interface.
2. The interfacial stress, *f*_*ij*_, which describes the excess stresses occurring in a system with an interface with respect to the bulk.^119^ The interfacial stress is a two-dimensional second-order tensor (which means that it can be described by a 2 × 2 matrix).

These seemingly unrelated concepts have a straightforward connection in liquid–liquid (or liquid–vapor) systems. Here, due to the rotational symmetry, the interfacial stress tensor can be described by a single number *f*, which is numerically equal to the IFE, γ = *f*.

For interfaces involving solids, the relationship between γ and *f* was first established by Shuttleworth,^130^ who derived the equation (which now bears his name) from thermodynamic considerations:

$$f_{ij}=\delta_{ij}\gamma +\frac{\partial \gamma }{\partial u_{ij}}$$ 6

where *u*_*ij*_ is the (*i*, *j*)^th^ element of the strain tensor of the interface, δ_*ij*_ is the Kronecker delta, and the indices *i*, *j* = 1, 2 refer to the two Cartesian axes within the interfacial plane. Herring^128,131^ gave a simple derivation of eq 6, and we reproduce its main features here. Let us consider a region of interface bounded by walls normal to the interface, and let us deform the interface by displacing the walls. In general, the interfacial work defined in eq 5 will change as

$$W^{XS}=\gamma \delta A+A\delta \gamma =\gamma A\underset{i=1,2}{\sum }u_{ii}+A\underset{i,j=1,2}{\sum }\frac{\partial \gamma }{\partial u_{ij}}u_{ij}$$ 7

For a plane normal to the interface, the material on one side of that plane exerts a force on the material on the other side. The excess force (with respect to its value in the bulk) is the *interfacial force* acting across this plane. The *interfacial stress* is now defined as the interfacial force per unit length of the line of intersection of the plane with the dividing surface. Because the orientation of the plane normal to the interface is arbitrary, this force can be expressed as ∑_*j*=1,2_ *f*_*ij*_ *n*_*j*_, where *n*_*j*_ are the components of the unit vector, **n̂**, perpendicular to the plane defining the interface. Equation 6 is obtained by equating eq 7 with $A\underset{j=1,2}{\sum }f_{ij}u_{ij}$ . Equation 6 has been subject to criticism since its formulation, and different authors have debated its validity (see ref 132 for a critical review). In ref 117, the authors derived it from first-principles, starting from a statistical mechanics description of a solid–liquid system, and showed that eq 6 matched numerical results obtained with molecular dynamics simulations of a Lennard-Jones system.

When a new interface is created in a liquid, its orientation does not matter and the energy associated with its creation will always be proportional to γ, so the IFE can be represented as a unique scalar quantity. When solids are involved, γ becomes a function of the orientation of the interface with respect to the crystal structure, usually indicated as γ­(**n̂**). The effect of the dependence of the IFE on the orientation of the crystal lattice in solids is macroscopically observable because the equilibrium shape of a crystal suspended in its melt depends on the relative values of the IFE for each different orientation of the crystal. Roughly speaking, because the free energy of the crystal should be a minimum, certain crystal planes will be preferred over others with higher values for the IFE. This determines the shape of a crystal. This argument was made more rigorous by Wulff^133^ in a theorem that now bears his name (although it is also known as the Gibbs–Curie–Wulff theorem, as the final form of the theorem includes contributions from Gibbs and Curie^134^). We use the statement of Wulff’s theorem as reported in ref 135, “When a crystal is in its equilibrium shape, under negligible gravitational or other body forces or surface constraints, there exists a point whose perpendicular distances from all faces of the crystal are proportional to their specific interfacial free energies; any other possible face not belonging to the equilibrium shape has a surface free energy such that a plane drawn with the corresponding orientation and distance from this point would lie entirely outside the crystal.” Knowing the IFEs of the different facets, the shape can be predicted by the Wulff construction using the so-called polar plot,^128,136,137^ making it possible to predict the shape of nanoparticles^138^ (which may not be composed of a single crystal and can have a complicated structure^139^).

From the discussion in this section, it is clear that the calculation of the IFE includes some complications intrinsic to the solid phase, namely a quantitative difference between interfacial stress and interfacial free energy and the dependence of the IFE on the crystal orientation of the solid surface exposed to the liquid. As a result, determining surface properties for solid–liquid interfaces using molecular simulations requires special techniques not needed for the liquid–liquid case. Indeed, the differences between solid–liquid and liquid–liquid interfaces are the main reason why there exists a large number of approaches for the calculation of the IFE, as discussed in detail in the following sections.

### 3.2 Failure of the Mechanical Route

The standard route to obtain the IFE for liquid–liquid systems was devised by Kirkwood and Buff^140^ in an equation stating that

$$\gamma =\int_{-\infty }^{+\infty }\left(P_{N}-P_{T}\left(z\right)\right)dz$$ 8

where *P*_*N*_ is the uniform pressure normal to the interface (which we are assuming to be oriented with its normal aligned to the *z*-direction), *P*_*T*_(*z*) is the pressure in the directions tangential to the interface (see eq 52 and the discussion in section 5.7 for its explicit definition), and both quantities can be obtained from the stress tensor using the virial expression for the pressure.^141^

In the original work of Kirkwood and Buff, the expression for the virial was given only for a pair potential.^140^ Today, the calculation of the pressure using the virial expression is a standard routine in MD simulation codes, with several variants presented in the literature to take into account all possible situations, e.g., the long-range component of Coulombic interactions calculated with the Ewald summation^142^ or the PPPM model^143^ or in polar and charged systems.^144^

However, eq 8 comes with a catch. As was shown in ref 117, the quantity calculated in eq 8 is the average excess interfacial stress, which, as we have just discussed, is equivalent to the IFE only when no solid phase is involved. Since in a solid–liquid system *f*≠γ,^145^ eq 8 can be safely employed to calculate the IFE only in a fluid–fluid system.

Unlike fluid–fluid interfaces, for solid–liquid interfaces there is no equivalent mechanical route to obtain γ from the pressure tensor, making it particularly challenging to determine the IFE, both experimentally and theoretically. As an example, the experimental value of γ at room temperature for the vapor–liquid interface of water is known to be 71.99 mJ m^–2^.^146^ Yet the experimental value of γ for the ice Ih–water interface at its melting point is uncertain, with estimates ranging between 25 and 35 mJ m^–2^.^147^ MD simulations must use the thermodynamic definition of the IFE, i.e., the work needed to form a new interface in the system, to calculate its value. As a result, one has to go beyond eq 8, which requires only a single equilibrium calculation at the interface, and use more complex methodologies, e.g., thermodynamic integration, to determine the free energy directly.

As we discussed in the previous section, γ is a function of the crystal orientation for solid–liquid interfaces and, therefore, whatever approach one chooses to determine the IFE must be able to discriminate between different orientations of the crystal. Because the calculation should be repeated for every possible orientation of the crystal, the computer resources required are likely to become an issue. Such difficulties explain why the accurate evaluation of γ for solid–liquid interfaces remained elusive until recent advances in simulation techniques, coupled with adequate computer power, provided solutions. The presentation of such simulation techniques is the objective of the rest of this review, starting in the next section.

### 3.3 Molecular Dynamics Free Energy Calculations

Due to the Shuttleworth equation, eq 6, the calculation of the IFE between a solid and a liquid cannot rely on direct determination of stresses within the system, which requires only the calculation of a well-defined quantity (the different components of the pressure tensor) in a single equilibrium calculation. Indeed, the only way to determine γ_*sl*_ is to resort to using its thermodynamic definition as the *reversible* work needed to create a new interface between two phases equilibrated at coexistence conditions, which is a much more formidable task.

At constant system volume, V (i.e., no surface excess volume), temperature, *T*, and number of particles for a single component, we can write γ_*sl*_ in terms of the variation of the (integral) Helmholtz free energy *F*

$$\gamma_{sl}=\frac{F^{\mathrm{fin}}-F^{\mathrm{init}}}{A}$$ 9

where the init and fin superscripts refer to the initial and final states, i.e., systems without and with an interface, respectively. Equation 9 is nothing other than the fundamental interface thermodynamic equation, eq 3, for a single component system, where we used the definition of [*F*]. If the creation of the interface includes a variation of volume (i.e., we have a surface excess volume), then from eq 4 we have

$$\gamma_{sl}=\frac{G^{\mathrm{fin}}-G^{\mathrm{init}}}{A}$$ 10

In turn, eq 9 and eq 10 are the starting points for obtaining γ_*sl*_ through MD simulations. There are several ways to determine a difference in free energy using MD simulations. These will be outlined in the rest of this review.

## 4 Indirect Methods to Determine the Solid–Liquid Interfacial Free Energy

The methods for determining the IFE when a solid is involved can be divided into two broad categories: direct and indirect. As the names suggest, the former group includes all methodologies in which the IFE is determined directly from the measurement of the reversible work required to create a unit area of the interface, whereas the latter group obtains the IFE as a byproduct of the calculation of some related quantity. We begin by briefly reviewing some of the most popular indirect methods. This class of methods uses simulations that mimic an experimental setup to obtain γ_*sl*_.

### 4.1 Contact Angle Methods

A liquid droplet is placed on a solid surface and, after equilibration, the contact angle θ, which is the angle formed between the tangent to the liquid surface and the solid surface at the point where they meet, is obtained from the density contour of the droplet.^148–152^ Young’s equation^89,121^ gives the relation between θ and the solid–liquid (*sl*), liquid–vapor (*lv*), and solid–vapor (*sv*) IFEs:

$$\cos\theta =\frac{\gamma_{sv}-\gamma_{sl}}{\gamma_{lv}}$$ 11

Obtaining γ_*sl*_ from Young’s equation requires independently computing γ_*lv*_ and γ_*sv*_. The former can be readily obtained by simulating the liquid in contact with the vapor and using eq 8. However, computing γ_*sv*_ can be challenging. For instance, in refs 151 and 152, γ_*sv*_ was calculated through thermodynamic integration of the energy difference between a bulk solid and a free solid slab from low temperature up to the melting point.

The determination of an IFE from the wetting property of the interface includes several complications that not only increase the complexity of the calculation but also affect the precision of the result. Young’s equation was derived on the hypothesis that the interface between solid and liquid is sharp, perfectly flat, and homogeneous. Unfortunately, this is not the case in a realistic system. One commonly used approximation considers the solid to be completely frozen. This is usually justified on the grounds that the mobility of atoms in the solid is so much smaller than that of atoms in the liquid that it can be ignored. The validity of this is difficult to establish without independently determining the solid–liquid IFE (which negates the purpose of using the method). Doubts have been expressed for systems in which the liquid can efficiently pack at the interface.^153^ If the frozen solid approximation is relaxed, then the sharpness and flatness of the interface may be compromised, particularly if the solid is near its melting temperature. The Neumann equation^154^ (see refs 155 and 156 for a more modern description) should then be used, as shown in ref 153. This is based on the geometrical description of the three interfaces present (solid–liquid, solid–vapor, and liquid–vapor) as the sides of a triangle (the Neumann triangle) and includes Young’s equation as a special case.

Another complication in the determination of the IFE through the contact angle, which is particularly relevant to MD simulations, comes from finite size effects. The IFEs in eq 11 are macroscopic intensive thermodynamic quantities and therefore their value should not depend on the size of the system considered. However, the contact angle for microscopic droplets is known to be different from the angle for macroscopic ones.^157^ Young’s equation must be modified to include an extra term, the contact line tension (the tension at the line of contact between the three phases), which takes this difference into account.^158,159^ This term depends on the inverse of the radius of curvature of the contact line (i.e., the radius of the droplet). It is negligible for macroscopic droplets, but it becomes significant for microscopic ones, i.e., for the sizes normally considered in MD simulations.

As an example of the intrinsic problem of the determination of the IFE using the contact angle, we consider an attempt to obtain γ_*sl*_ for pure NaCl by simulating a drop of liquid NaCl on top of the halite solid at its melting temperature.^151,152^ The value obtained, 36 mJ m^–2^,^151,152^ is much smaller than values calculated using different methods (90–100 mJ m^–2^) such as classical nucleation theory,^160,161^ mold integration,^161^ capillary fluctuations,^162^ or test area^163^ (see Table 4 in section 8). The fact that the only outlier value of γ_*sl*_ is the one determined through the contact angle methods suggests that such calculations may be affected by one or more of the problems discussed earlier, the most likely being a finite-size effect.

### 4.2 Classical Nucleation Theory

CNT^164–166^ predicts that the *thermodynamic* barrier associated with the formation of a crystalline nucleus in a metastable parent liquid or saturated solution is given by^167^

ΔG=γA−|Δμ|N 12

where *N* and A are the number of growth units and the surface area of the nucleus, respectively. If we consider the solidification process, then Δμ is the chemical potential difference between the crystal and the liquid, where the liquid is the melt.^4,10^ Instead, if the liquid is a solution of the solid, then we must use the identity Δμ = *k*_*B*_ *T* ln ζ, where ζ = *a*_0_/*a*_sat_ is the supersaturation ratio, namely the ratio of the solute activity in the solution, *a*_0_, to the solute activity at saturation, *a*_sat_ (the supersaturation is usually indicated in the literature with the symbol *S*. Here, however, we reserve that letter to represent the entropy and we will use the symbol ζ instead).^168^ The first term on the right-hand side of eq 12 increases the nucleation barrier as the square of the nucleus diameter, whereas the second one decreases it as the cube of the same quantity. The competition between these two terms gives rise to a maximum in Δ*G*, denoted by Δ*G*_crit_, which is the free energy barrier to the formation of a critical nucleus of the new phase beyond which growth is spontaneous.^167^ Δ*G*_crit_ can be written as

$$\Delta G_{\mathrm{crit}}=\frac{16\pi {\gamma_{sl}}^{3}}{3{\rho_{s}}^{2}|\Delta \mu {|}^{2}}=\frac{16\pi {\gamma_{sl}}^{3}}{3{\rho_{s}}^{2}{k_{B}}^{2}T^{2}{\left(\ln\zeta \right)}^{2}}$$ 13

where ρ_*s*_ is the number density of the solid at the temperature and pressure of interest. Usually, we want to determine Δ*G*_crit_ from the parameters on which it depends (supersaturation, IFE, etc.). In turn, Δ*G*_crit_ allows us to determine the nucleation rate, which is essential in different applications, such as (for the crystallization case) precipitation of nanoparticles^169–171^ and in general in the study of macroscopic models such as the population balance equation.^172^ The use of γ_*sl*_ to determine the rate of nucleation in the context of CNT will be discussed here and in section 9. However, eq 13 can also be used in the other direction: by knowing Δ*G*_crit_ and the other factors in eq 13, it is possible to determine γ_*sl*_. In this section we provide a brief account on how to evaluate each factor in eq 13 (except γ_*sl*_).

The numerical factor 16π/3 in eq 13 assumes a spherical nucleus; the equivalent form factor for other geometries can be calculated (see e.g., ref 173). The chemical potential difference can be obtained through thermodynamic integration for both liquid cases: the melt and the supersaturated solution. For the melt case, we use the fact that μ is the same for both phases at the melting point.^174^ For the solution case, we use the identity Δμ = *k*_*B*_ *T*ln ζ. However, computing Δ*G*_crit_ is more challenging, as special rare event techniques such as umbrella sampling^175^ or metadynamics^176,177^ are needed to bias the formation of the critical nucleus from the liquid and compute the associated free energy change.^116,160,178–182^ Δ*G*_crit_ can also be obtained using the seeding method.^183–186^ This identifies the critical nucleus, which, by definition, has an equal chance of growing or redissolving, by inserting nuclei of different sizes in the liquid. This approach requires us to distinguish between particles in the critical nucleus (i.e., the stable phase) and the surrounding metastable phase. By selecting a criterion to make such a choice we are defining an *order parameter* (i.e., a quantity that measures a particular type of structuring in the system) to count the particles within the critical nucleus, *N*_crit_.^186–188^ As an example of an order parameter, we can use the coordination number, that is, the number of solute particles within a certain distance of a given solute particle. Then, for a specific value of the coordination number, a solute particle belongs to a nucleus only if it is surrounded by a number of other solute particles within a certain distance, equal to or greater than the coordination number. As shown in ref 187, the size of the nucleus depends on this coordination number: the larger it is, the smaller will be the critical nucleus identified. Using *N*_crit_, we can obtain Δ*G*_crit_ through the following CNT expression:

$$\Delta G_{\mathrm{crit}}=\frac{N_{\mathrm{crit}}\left|\Delta \mu \right|}{2}$$ 14

It follows that the evaluation of γ_*sl*_ depends on the mathematical criterion used to determine the size of the crystal cluster. However, it has been shown that by using a judicious choice of order parameter, consistent results between CNT, seeding and direct methods can be obtained for γ_*sl*_.^186,189,190^

Regardless of the approach used to compute Δ*G*_crit_, eq 13 is typically used to obtain γ_*sl*_ for several state points where the crystal is more stable than the melt (or solution, i.e., ζ > 1). Then γ_*sl*_ at coexistence is obtained through an extrapolation. This extrapolation involves the usual assumption that γ_*sl*_ is a scalar independent of the morphology of the critical nucleus, which can be identified with the IFE averaged over the crystal orientations that the nucleus exposes to the liquid. The extrapolation can be avoided by using eq 12 under coexistence conditions, where the second term on the right-hand side of the equation is zero (|Δμ| = 0).^191^ However, in such conditions, the free energy does not reach a maximum; instead, it increases monotonically with γA . Therefore, the calculation of the IFE depends on the size and shape of the cluster, which are parameters that cannot be unambiguously defined. Moreover, curvature corrections must be included in order to get γ_*sl*_ for a flat interface out of free-energy calculations of finite clusters.^191^

While CNT can be a useful framework to get estimates of the average of γ_*sl*_ across the different faces at coexistence from the free energy of critical clusters, one should be aware of the shortcomings of this approach:

1. Information about the complete function γ_*sl*_(**n̂**) is lost.
2. The route to obtain γ_*sl*_ depends on arbitrary criteria to determine the cluster size for both seeding and free-energy calculations of clusters.
3. Extrapolations to coexistence are required for seeding and for free-energy calculations away from coexistence.
4. To extract an IFE we must use the capillary approximation or correct for the finite curvature of the nucleus. For small critical nuclei, we must also face the problem of drawing a meaningful distinction between the interface and the bulk. These issues will be addressed further in section 8 and section 9.

### 4.3 Capillary Fluctuations Method

The capillary fluctuations method was proposed by Hoyt, Asta, and Karma^192^ and extended by Davidchack, Morris, and Laird.^193,194^ It is one of the most popular *indirect* methods for determining values of the IFE for different orientations of the crystal lattice. It is based on the observation that a diffuse (or rough, i.e., not faceted) solid–liquid interface will fluctuate because of thermal energy, as shown for an ice–liquid interface in Figure 1.^195^ We call these fluctuations “capillary fluctuations”. They have the property that they are enhanced by thermal energy and damped by a quantity called the “interfacial stiffness”, *γ̅*_*sl*_, which will be defined in the next paragraph.

**Figure 1:**
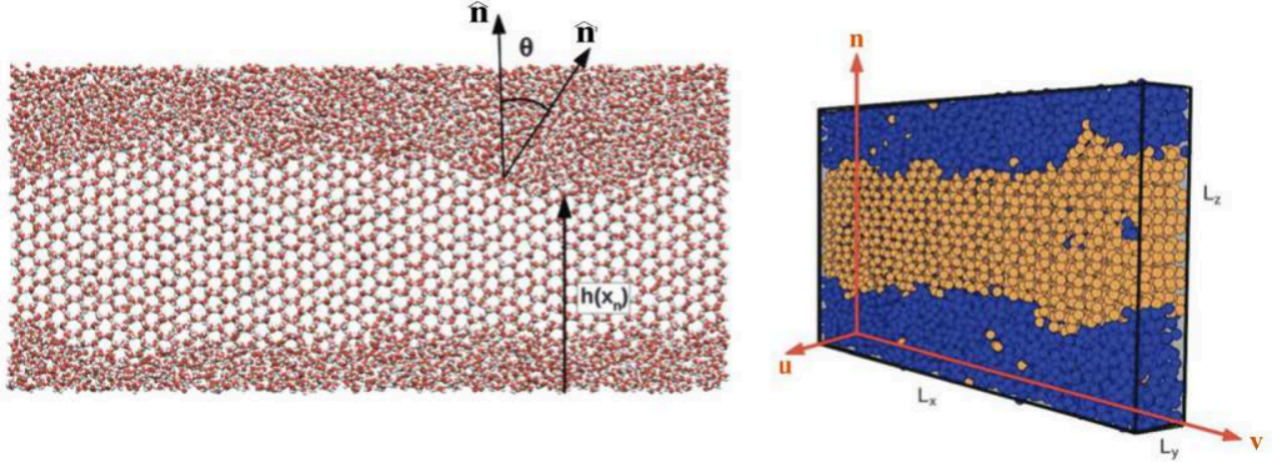
(Left) Front view of a simulation snapshot of hexagonal ice coexisting with liquid water (water molecules are represented as red and white spheres for oxygen and hydrogen atoms, respectively). The angle θ that quantifies the deviation with respect to the average interfacial orientation is defined in the figure. A point of the function *h*(*x*_*n*_) that defines a discretized interfacial profile in the real space is indicated. (Right) View of the simulation box showing the elongated strip geometry of the *x* – *y* side where the solid interface is exposed to the liquid. Ice and liquid molecules are depicted as orange and blue spheres respectively to enhance the visual contrast between both phases. This figure was adapted with permission from ref 195. Copyright 2014 Royal Society of Chemistry.

The capillary fluctuations method provides a direct calculation not of γ_*sl*_ but rather of the stiffness *γ̅*_*sl*_, which is equal to γ_*sl*_ plus the curvature of the dependence of γ_*sl*_ on the orientation of the interface:^196,197^

$${\mathrm{\gamma ̅}}_{sl}={\mathrm{\gamma ̅}}_{sl}\left(\theta =0\right)={\left(\gamma_{sl}\left(\theta \right)+\frac{\partial^{2}\gamma_{sl}\left(\theta \right)}{\partial \theta^{2}}\right)}_{\theta =0}$$ 15

where θ is the angle formed by the normal to the average interfacial plane, represented by the vector **n̂** in Figure 1, and the normal to a local fluctuation with respect to the average orientation, represented by the vector **n̂**′ in Figure 1.

Knowing the symmetry of the crystal, γ_*sl*_(θ) can be expanded around θ = 0, typically using either spherical^195^ or cubic^192,195,198^ harmonics. Combining the expansion and eq 15, a set of equivalent expansions is obtained for the stiffness. These expansions depend on γ_*sl*_ and several coefficients (typically 3–4 coefficients are needed). Therefore, the stiffness has to be obtained for different interface orientations and directions of wave propagation in order to extract the coefficients of the expansions by solving a system of equations. These coefficients, in turn, are used to calculate γ_*sl*_ for each crystal orientation. The capillary fluctuations method is particularly well suited to study the variation of the interfacial free energy with respect to the crystal orientation.

The determination of the stiffness *γ̅*_*sl*_ is crucial, and we now sketch how it is obtained, referring the interested reader to the literature reported in this section for a more detailed account of the methodology. The quantity we need to determine the stiffness is the interfacial profile *h*(*x*_*n*_). This is first computed for *N* discrete points along the elongated side of the simulation box (see Figure 1) and then transformed to Fourier space:

$$h\left(q\right)=\frac{1}{N}\overset{N}{\underset{n=1}{\sum }}h\left(x_{n}\right)e^{iqx_{n}}$$ 16

where the wave vector *q* is a multiple of 2π/*L*_*x*_. Although capillary waves propagate in 2D,^199^ in order to reduce system size and to control the propagation direction, the simulation box is built so that the interface is a thin elongated strip (see Figure 1, where *L*_*x*_ ≫ *L*_*y*_ for this purpose). In the example of Figure 1, the interface is exposed in the *x* – *y* plane, and the capillary waves propagate along the *x* direction. Then, the stiffness is obtained for a given orientation of the solid with respect to the liquid and for a given direction of propagation of the capillary waves. In order to ensure the stable interface required by the method, the simulation must be run in the NVT ensemble at the melting temperature and with an intermediate density between those of the coexisting solid and molten phases. A common issue in this type of calculation is the presence of stresses: these can be avoided by setting the edges tangential to the interface (i.e., *L*_*x*_ and *L*_*y*_ in Figure 1) to the value corresponding to a solid equilibrated at coexistence conditions.

Capillary wave theory^200–202^ uses the equipartition theorem to provide a relationship between the amplitude of *h*(*q*) and the stiffness *γ̅*_*sl*_ (see eq 15):

$$\left\langle \right|h\left(q\right){|}^{2}\rangle =\frac{k_{B}T}{A{\mathrm{\gamma ̅}}_{sl}q^{2}}$$ 17

where A is the interfacial area (*L*_*x*_·*L*_*y*_ in the nomenclature of Figure 1) and |*h*(*q*)| is given by eq 16. Equation 17 is valid in the limit of small *q* vectors, i.e., long wavelength capillary fluctuations, and reveals that the size of capillary fluctuations is inversely proportional to the interfacial stiffness.

The capillary fluctuations method was first applied to various pure metals, alloys, and other atomic systems with the fcc and bcc crystal structures, such as Ni,^192,203^ Cu,^97^ Al,^194^ Fe,^204^ hard spheres,^193,205–207^ and Lennard-Jones.^208^ It was then extended to other systems and solid structures such as Mg with an hcp solid,^209,210^ sodium chloride,^162^ water with a hexagonal ice solid,^195^ succinonitrile,^211^ charged colloids with a bcc solid,^212^ and the dipolar Stockmayer fluid with an fcc solid.^213^

## 5 Direct Methods to Determine the Solid–Liquid Interfacial Free Energy

Direct simulation methods are based on the thermodynamic definition of the IFE as the reversible work required to create a unit area of interface between a solid and a liquid phase under solid–liquid coexistence conditions. Such methods require the construction of a thermodynamic transformation path from a system without an interface (for example, isolated bulk solid and liquid systems under coexistence conditions) to a system containing the interface. The reversible work or, equivalently, the free energy difference between the two states can be calculated by a variety of free-energy calculation methods^214^ such as Thermodynamic Integration (TI), free-energy perturbation, Bennett acceptance ratio and nonequilibrium switching (employing the Jarzynski identity^215^). Because the different methodologies presented here are mostly based on the TI procedure, we include an introduction to Thermodynamic Integration in appendix A and discuss here only the specific details of each methodology. A critical discussion of the methods, aiming to guide interested readers in selecting the approach that best suits their needs, can be found in appendix B.

### 5.1 Cleaving Methods

The cleaving approach was used for the first time by Broughton and Gilmer^216^ to calculate the solid–liquid IFE for a truncated Lennard-Jones potential, although the idea was first proposed by Miyazaki et al. for the liquid–vapor interface.^217^ The method is based on the calculation of the free energy change along a reversible path that starts from separate solid and liquid bulk systems under coexistence conditions and ends with the solid–liquid interfacial system under the same conditions. The free energy change is thus related to the creation of the interface, and the IFE is determined as the ratio of this change to the area of the new interface.

The calculation starts by preparing separate solid and liquid systems under solid–liquid coexistence conditions with periodic boundary conditions in all directions. The systems should have the same size in the directions within the interfacial plane (typically aligned with the *x*- and *y*-axes, while the *z*-axis is taken to be normal to the interface). The transformation path is then constructed with the help of external *cleaving* potentials playing the dual role of splitting the solid and liquid bulk systems along a plane (chosen to be between two crystal layers in the solid system and chosen arbitrarily in the liquid system), so they can be combined into the interfacial system later, and introducing a structure in the liquid phase near the chosen plane that is compatible with the crystal structure of the solid phase.

The transformation path consists of four basic steps, as shown in Figure 2:

1. Insert the cleaving potential into the solid system along a plane between crystal layers of a given orientation (called the cleaving plane).
2. Insert the cleaving potential in the liquid system with the same dimensions as the solid system in the directions tangential to the plane.
3. Gradually (and reversibly) switch the interactions from solid–solid and liquid–liquid to solid–liquid across the cleaving plane while maintaining the cleaving potentials.
4. Remove the cleaving potentials from the combined solid–liquid system.

**Figure 2:**
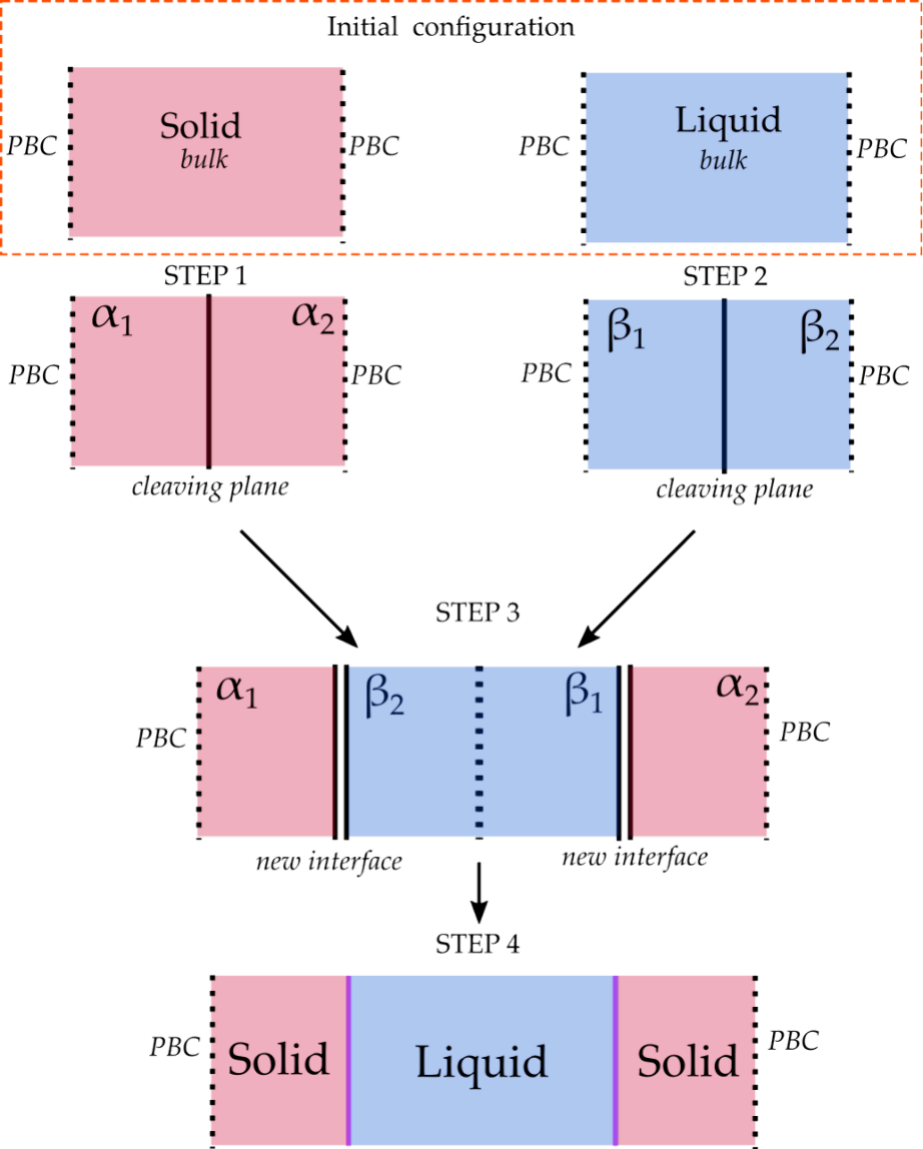
Schematic illustration of the cleaving procedure, with the four steps described in the text highlighted. The initial point is represented by two different systems, solid bulk and liquid bulk. The final point is represented by a single system with two new interfaces between the solid and liquid phases. Labels α_1_, α_2_ (for the solid) and β_1_, β_2_ (for the liquid) help to identify parts of the solid and liquid systems that are put into contact in step 3.

Each transformation step can be implemented using a standard coupling parameter approach,^214^ where the total potential energy of the system in step *n*, $U_{n}\left(\lambda \right)$ , depends on a coupling parameter λ such that changing the parameter from 0 to 1 transforms the system from the thermodynamic state at the beginning of the step to that at the end. In its simplest implementation, the potential energies $U_{n}\left(\lambda \right)$ take the form

$$U_{1}\left(\lambda \right)=U_{s}+\lambda \Phi_{s}$$ 18a

$$U_{2}\left(\lambda \right)=U_{l}+\lambda \Phi_{l}$$ 18b

$$U_{3}\left(\lambda \right)=\left(1-\lambda \right)\left(U_{s}+U_{l}\right)+\lambda U_{sl}+\Phi_{s}+\Phi_{l}$$ 18c

$$U_{4}\left(\lambda \right)=U_{sl}+\left(1-\lambda \right)\left(\Phi_{s}+\Phi_{l}\right)$$ 18d

where *U*_*s*_, *U*_*l*_, and *U*_*sl*_ are the potential energies of the solid, liquid, and combined systems, respectively, Φ_*s*_ and Φ_*l*_ are the cleaving potentials introduced in the solid and liquid systems. The simple linear dependencies on λ in the above equations can be replaced by any continuous functions *g*(λ) such that *g*(0) = 0 and *g*(1) = 1.

In the TI formulation of the coupling parameter approach, the reversible work, *W*_*n*_ (*n* = 1, 2, 3, 4), required to perform each step is calculated as

$$W_{n}=\int_{0}^{1}{\left\langle \partial U_{n}/\partial \lambda \right\rangle }_{\lambda }d\lambda$$ 19

where ⟨···⟩_λ_ denotes an average over the equilibrium state at a fixed value of λ. The solid–liquid IFE is then given by

$$\gamma_{sl}=A^{-1}\overset{4}{\underset{n=1}{\sum }}W_{n}$$ 20

where A has the usual meaning of the interfacial area. There is considerable flexibility in the design of the cleaving potentials Φ_*s*_ and Φ_*l*_. In their calculation of the IFEs of (100), (110), and (111) solid–liquid interfaces in the truncated Lennard-Jones system at the triple point, Broughton and Gilmer^216^ used a simple repulsive potential for the solid system (in Lennard-Jones units)

$$\Phi_{s}=3e^{-\xi_{1}z^{4}}$$ 21

and a combination of repulsive and attractive potentials for the liquid system

$$\Phi_{l}=3e^{-\xi_{2}z^{4}}-\left[\chi_{1}+\chi_{2}F\left(x,y\right)\right]e^{-\xi_{3}{\left(z-z_{\min}\right)}^{2}}$$ 22

where *z* is the distance to the cleaving plane and *z*_min_ is the distance from the cleaving plane to the nearest crystal layer (which is equal to half the interlayer distance for a given crystal orientation). The attractive part of the liquid cleaving potential is modulated by the function *F*(*x*, *y*), which has local minima corresponding to the locations of particles in the crystalline structure in order to induce formation of crystal-like layers that match the crystal layers in the solid system at the cleaving plane. Parameters χ_1_, χ_2_, ξ_1_, ξ_2_, and ξ_3_ are chosen to.

1. Ensure that atoms in solid and liquid systems do not mix during Step 3.
2. Introduce sufficient structure in the first liquid layer to match the corresponding crystal layers in the solid systems.
3. Perturb the systems as little as possible in order to minimize the amount of reversible work performed in Steps 1 and 2.

Note that Broughton and Gilmer^216^ used different symbols for χ_1_, χ_2_ and ξ_1_, ξ_2_, ξ_3_. We modified them to avoid confusion with other quantities defined in this work.

This last requirement is satisfied by making the cleaving potential relatively short-range (i.e., choosing large values for ξ_1_, ξ_2_, and ξ_3_). While this is fine for Step 1, in Step 2 it leads to a large uncertainty in the calculated reversible work due to the presence of a first-order transition associated with the formation of crystalline layers in the liquid system. A nucleation barrier is created (which must be crossed), resulting in a hysteresis loop in the process of switching the cleaving potential on and off in Step 2. Broughton and Gilmer noted that the size of the loop depended on the range of the attractive part of the cleaving potential Φ_*l*_. To reduce it, they increased the range of the attractive potential in regions of the liquid away from the cleaving plane. So, whereas ξ_3_ had values between 40 and 100 depending on the orientation of the interface, for *z* ≤ *z*_min_, ξ_3_ = 4.0 for *z* > *z*_min_ (we stress here that we are working in Lennard-Jones units; see Table 2 and eq 6 in ref 216). The results obtained by Broughton and Gilmer (see Table 2) had a precision of about 3–6%, which was not sufficient to resolve the dependence of γ_*sl*_ on the orientation of the solid surface in contact with the liquid. Further development of the cleaving method^96^ and the introduction of the capillary fluctuations method^208^ were necessary to achieve a precision sufficient to resolve the different values of γ_*sl*_(**n̂**).

The cleaving method was adapted by Davidchack and Laird to obtain the first direct calculation of the IFE of hard-sphere crystal–melt interfaces, with orientations corresponding to the (100), (110), and (111) crystal planes.^218^ Because the event-driven implementation of the time evolution of a hard-sphere system is conceptually very different from the time-stepping numerical solution of the equations of motion for continuous potentials, the cleaving method used to calculate the hard-sphere crystal–melt IFE needed an adaptation. To achieve this, Davidchack and Laird cleaved the solid and liquid systems using a pair of moving “walls” placed on either side of the cleaving plane and interacting only with the hard spheres of the solid and liquid systems (which we call the “system spheres”) on the opposite side of the plane. In order to induce the correct structure in the liquid system and minimize the perturbation of the solid system, the walls were made of hard spheres (which we call the “wall spheres”) of the same diameter as the system spheres but fixed at the ideal crystal positions consistent with the solid layers adjacent to the cleaving plane. At the start of Steps 1 and 2, the two walls were placed sufficiently far from the cleaving plane that they did not interact with the system spheres. During the steps, the two walls moved closer to the cleaving planes and started colliding with the system spheres. At the end of the steps, the walls reached positions where the system spheres on the opposite sides of the cleaving plane no longer collided with each other. Thus, the cleaving of the systems in Steps 1 and 2 was achieved, and rearrangement of the boundary conditions in Step 3 did not require additional work (i.e., *W*_3_ = 0). Step 4 was then performed on the combined solid–liquid system by moving the walls back to their initial positions. The original implementation of the cleaving method for hard-sphere systems^218^ contained an error that was later corrected;^115^ here we outline the corrected version. Let the spheres of the system have diameter σ. The spheres comprising the walls have the same diameter and are located at positions **R**_*j*_^(1, 2)^ = (*X*_*j*_^(1, 2)^, *Y*_*j*_^(1, 2)^, *Z*_*j*_^(1, 2)^) in ideal crystal layers with the same orientation as the solid system in Step 1. Depending on the orientation, each wall consists of one or two layers. The positions of the walls with respect to the cleaving plane are −*z*_*w*_ for Wall 1 and +*z*_*w*_ for Wall 2, where *z*_*w*_ is half the distance between the nearest layers of the two walls: $z_{w}=\frac{1}{2}\left(\min_{j}Z_{j}^{\left(2\right)}-\max_{j}Z_{j}^{\left(1\right)}\right)$ . The system spheres interact with the wall spheres as follows: a sphere collides with one of the wall spheres only if, at the moment of collision, it overlaps with a sphere belonging to the other wall. Therefore, during the simulation, collisions with wall spheres are first predicted and then, while processing a sphere collision with one wall, it is checked to see if the sphere overlaps with the other wall. If it does, the collision takes place; otherwise, the sphere continues to move in the same direction as before. This interaction can be described by the following cleaving potential exerted by the moving walls on the system spheres:

$$\Phi_{s}\left(r\right)=\Phi_{l}\left(r\right)=\{\begin{array}{ll} \infty , & \mathrm{if}\;\min_{j}\left|r-R_{j}^{\left(1\right)}\right|<\sigma \;\mathrm{and}\;\min_{j}\left|r-R_{j}^{\left(2\right)}\right|<\sigma \\ 0, & \mathrm{otherwise} \end{array}$$ 23

The cleaving process in Steps 1 and 2 is illustrated in Figure 3, where the shaded regions are inaccessible to the system spheres. When the two walls are placed sufficiently far apart, as in Figure 3(a), they do not interact with the system spheres. As the two walls move closer together, inaccessible regions appear and grow, so that at some point, as in Figure 3(b), they form a fully connected region which the spheres cannot cross. In order to prevent the system spheres from colliding across the cleaving plane, the walls should be moved to the position shown in Figure 3(c), where the minimal thickness of the inaccessible region is larger than the diameter of the hard sphere.

**Figure 3:**
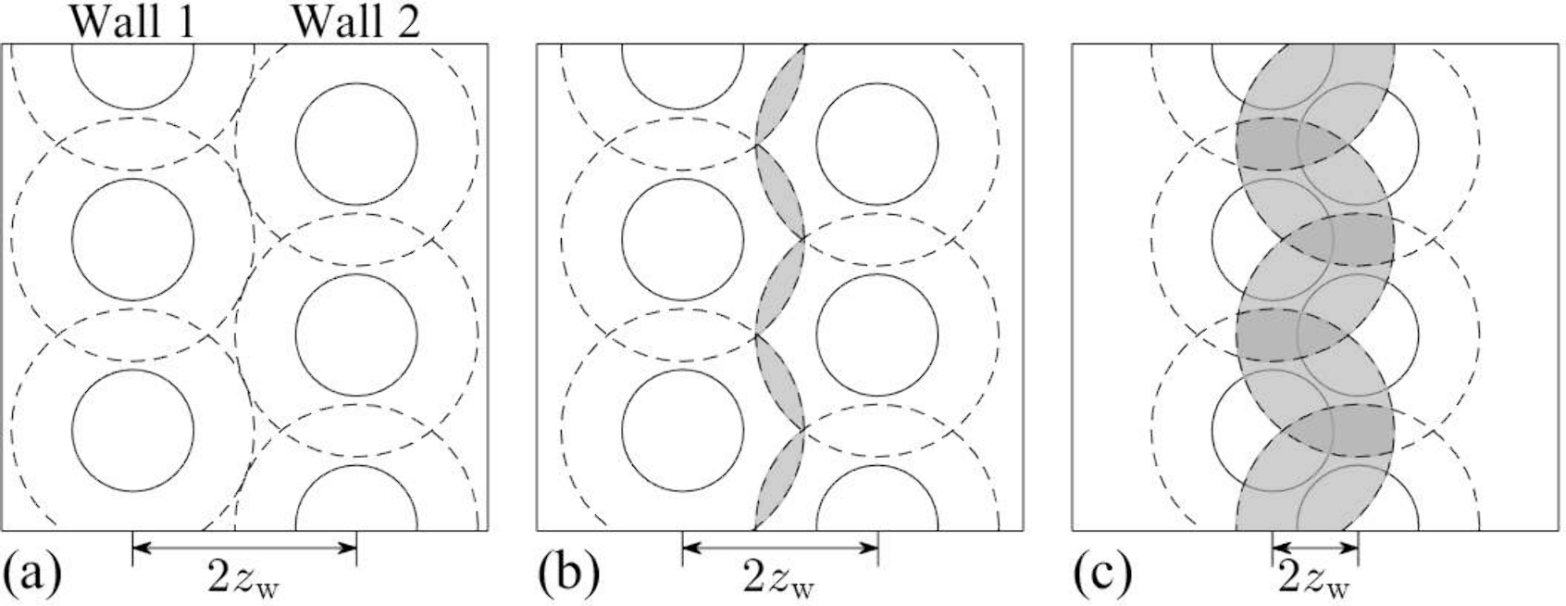
Illustration of the cleaving walls needed to calculate the IFE of the (100) crystal–melt interface in a hard-sphere system. Solid circles outline the wall spheres of diameter σ. Dashed circles outline spheres of radius σ centered at the wall spheres. Shaded regions indicate the excluded volume introduced by the cleaving walls, i.e., where the cleaving walls potential is infinite (see eq 23). (a) Initial position of the walls, where they do not interact with the system. (b) Intermediate wall position, where the system sphere can no longer pass through the cleaving plane. (c) Final position of the walls, where the system spheres cannot collide across the cleaving plane.

If the TI approach is used to calculate the reversible work for step *n* of the moving wall method, the system is equilibrated at a number of intermediate positions of the walls, and the average value of the pressure on the walls is computed as a function of the wall position *P*_*n*_(*z*_*w*_). The reversible work is then obtained by numerically evaluating the integral

$$W_{n}=A\int_{z_{i}}^{z_{f}}P_{n}\left(z_{w}\right)dz_{w},n=1,2,4$$ 24

where *z*_*i*_ and *z*_*f*_ are the initial and final wall positions, respectively.

Another approach to computing the reversible work in the cleaving method is to perform *nonequilibrium work measurements*. The transformation (either by changing λ or by moving walls) is performed over a finite time interval. This approach is based on the Jarzynski equality,^215,219^ which relates the work W done on the system during a nonequilibrium process starting from initial states sampled at equilibrium with temperature *T* with the reversible work *W* between the same initial and final thermodynamic states:

$$\left\langle e^{-W/k_{B}T}\right\rangle =e^{-W/k_{B}T}$$ 25

where the angle brackets denote the average over an ensemble of nonequilibrium processes starting from an equilibrium ensemble of initial states. Nonequilibrium work measurements are typically preferable to TI because they provide more efficient calculations and better error estimates for reversible work, especially when nonequilibrium work measurements can be performed in both forward and (time-)­reversed directions. In this case, as shown by Crooks,^220^ the probability distributions of forward (F) and (time-)­reversed (R) nonequilibrium work measurements are related by the formula

$$P_{F}\left(W\right)=e^{\left(W-W\right)/k_{B}T}P_{R}\left(-W\right)$$ 26

where the reverse process starts from equilibrium at temperature *T* and the switching protocol mirrors that of the forward process. Then the reversible work can be calculated from averages over these distributions

$$e^{-W/k_{B}T}=\frac{{\left\langle h\left(W\right)\right\rangle }_{F}}{{\left\langle h\left(-W\right)e^{-W/k_{B}T}\right\rangle }_{R}}$$ 27

where angle brackets with subscripts F and R denote averages over nonequilibrium work measurements in the forward and reverse directions, respectively, and

$$h\left(W\right)={\left(1+\frac{n_{F}}{n_{R}}e^{\left(W-W\right)/k_{B}T}\right)}^{-1}$$ 28

provides an asymptotically unbiased estimator for *W* with minimal variance, with *n*_F_ and *n*_R_ being the numbers of independent forward and reverse measurements, respectively.

The moving walls method was adapted for application to continuous potentials and applied to the truncated Lennard-Jones potential, improving the precision of the results obtained by Broughton and Gilmer at the triple point as well as calculating the solid–liquid IFE at temperatures *T* = 1.0 and 1.5 ϵ/*k*_*B*_.^96^ The cleaving walls potential was constructed from a short-range repulsive potential ϕ­(*r*) centered at the ideal crystal positions **R**_*j*_^(1, 2)^ and defined as the minimum of the two wall potentials

$$\Phi \left(r,z\right)=\min\left(\Phi_{1}\left(r,z\right),\Phi_{2}\left(r,z\right)\right)$$ 29

where

$$\Phi_{1,2}\left(r,z\right)=\underset{j}{\sum }\phi \left(|r-R_{j}^{\left(1,2\right)}\pm \mathrm{n̂}z|\right)$$ 30

with *z* being the distance of the cleaving walls from the cleaving plane and **n̂** denoting a unit vector normal to the cleaving plane. The cleaving walls potential was used with TI in ref 96, while Mu and Song obtained similar results using the same cleaving walls potential with nonequilibrium work measurements^221^ (see Table 2). A similar cleaving walls potential was used to calculate the IFE for inverse power potentials (also known as soft spheres), *U*(*r*) = *r* ^–*n*^, with *n* = 6, 7, 8, 10, 12, 14, 20, 30, 50, and 100 for the fcc–liquid interface and *n* = 6, 7, and 8 for the bcc–liquid interface.^222^

The cleaving method was further extended to calculate IFEs for ice–water interfaces, modeled using rigid-body TIP4P and TIP5P water potentials.^223,224^ In order to induce the formation of crystal layers with correctly oriented molecules in Step 2, attractive cleaving *wells* potentials were introduced instead of repulsive walls. The short-range attractive potential ϕ­(*r*) was placed at the ideal crystal positions **R**_*j*_, modulated by an orientation-dependent factor θ

$$\Phi \left(r,q\right)=\underset{j}{\sum }\phi \left(|r-R_{j}|\right)\theta \left(q,Q_{j}\right)$$ 31

where **r** and **q** are the translational and rotational coordinates of a molecule, respectively, and **Q**_*j*_ is the orientation of a molecule in the ideal crystal at position **R**_*j*_. The attractive potential has the form ϕ­(*r*) = *d*_*w*_[(*r*/*r*_*w*_)^2^ – 1]^3^ for *r* < *r*_*w*_ and zero otherwise, where *d*_*w*_ and *r*_*w*_ are the well depth and range parameters, respectively. The orientation-dependent factor has the form of a dot product between unit vectors directed from the oxygen atom to the midpoint between the hydrogen atoms in a water molecule: θ­(**q**, **Q**) = **n**(**q**)·**n**(**Q**). The results obtained with the cleaving method had sufficient precision to discriminate between the different values of γ_*sl*_(**n̂**) and show that the basal interface has the lowest IFE.^224^ The attractive wells external potential (without the orienting factor) was also used successfully to calculate the IFE of an Fe bcc crystal–liquid interface modeled using the embedded atom method potential.^225^

More recently, the cleaving approach has been extended to more complex systems, such as Ag–ethylene glycol,^226^ orcinol–chloroform, and orcinol–nitromethane^227^ interfaces. Because these are heterogeneous interfaces (interfaces between dissimilar materials that do not mix), the cleaving process can be simplified by switching off the interactions across the cleaving plane while introducing cleaving potentials in Steps 1 and 2. In this case, the rearrangement of boundary conditions in Step 3 does not require any work and can be performed instantaneously.

### 5.2 Mold Integration

Mold integration calculates γ_*sl*_ by reversibly inducing the formation of a crystalline slab in the fluid under coexistence conditions.^228^ The free energy needed to form such a crystalline slab, Δ*G*, is related to γ_*sl*_ by (see eq 10):

$$\gamma_{sl}=\Delta G/A$$ 32

Because the formation of the slab is performed under coexistence conditions, the fluid and the crystal have the same chemical potential. Hence, Δ*G* is just the specific IFE times the area of the interface. This corresponds to twice the cross-sectional area of the simulation box because the slab exposes two interfaces to the fluid (see Figure 4). To induce the formation of the crystal phase, mold integration uses a mold of potential energy wells located at the equilibrium positions of the perfect crystal lattice under coexistence conditions. Figure 4 shows a snapshot of the mold used to calculate γ_*sl*_ for the (100) plane of hard spheres.^228^ Each potential well must be small enough to accommodate no more than one particle. When the mold is turned off, particles freely diffuse in the liquid (see Figure 4, top), but when the mold is on, every well contains a particle. A crystal slab can then be induced in the liquid for a suitably parametrized mold. Although wide or shallow wells cannot induce crystal slab formation, if the potential is sufficiently narrow and deep to confine the particle at the crystal lattice position, the mold can induce a slab (see Figure 4, bottom), giving rise to two crystal–fluid interfaces. By gradually switching on the interaction between the mold and the particles, the work of formation of the crystal slab under coexistence conditions can be obtained by TI using the following expression:

$$\gamma_{sl}\left(r_{w}\right)=\frac{1}{A}\left(\epsilon_{m}N_{w}-\int_{0}^{\epsilon_{m}}d\epsilon \left({\left\langle N_{fw}\left(\epsilon \right)\right\rangle }_{NP_{x}T}\right)\right)$$ 33

where *N*_*w*_ is the total number of wells in the mold, and ⟨*N*_*fw*_(ϵ)⟩ is the average number of filled wells during an *NP*_*x*_ *T* simulation with wells of depth ϵ (the barostat in the simulation is applied only in the direction perpendicular to the crystal-fluid interface to avoid deforming the perfect equilibrium lattice). TI is then performed (using eq 33) along a path where the depth of the mold wells is gradually increased to its maximum value, ϵ_*m*_, yielding γ_*sl*_. To ensure the reversibility of this path, the crystal structure induced by the mold must melt quickly (in approximately the time it takes for a liquid particle to diffuse its own molecular diameter) when the interaction between the potential wells and the fluid is turned off. The integration must therefore be performed at well radii (*r*_*w*_) that are wider than the optimal one, *r*_*w*_^*o*^, at which the crystal slab is fully formed, so that its stability no longer depends on the mold–fluid interactions. In practice, as proposed in ref 228, γ_*sl*_(*r*_*w*_) can be estimated for several values of *r*_*w*_ > *r*_*w*_^*o*^ and then extrapolated to *r*_*w*_^*o*^, which is the well radius that provides the desired value of γ_*sl*_. The width chosen for *r*_*w*_^*o*^ is based on selecting the intermediate potential well radius between two different regimes:

1. A regime in which there is no induction time for the formation of the crystal slab at maximum potential well depth (i.e., 8–10 *k*_*B*_ *T* and small well radius).
2. A regime where, using the same potential well depth, the formation of the crystal slab must still overcome some activation energy barrier, and thus the system exhibits an induction time before the slab crystal grows (i.e., for wider potential wells).

**Figure 4:**
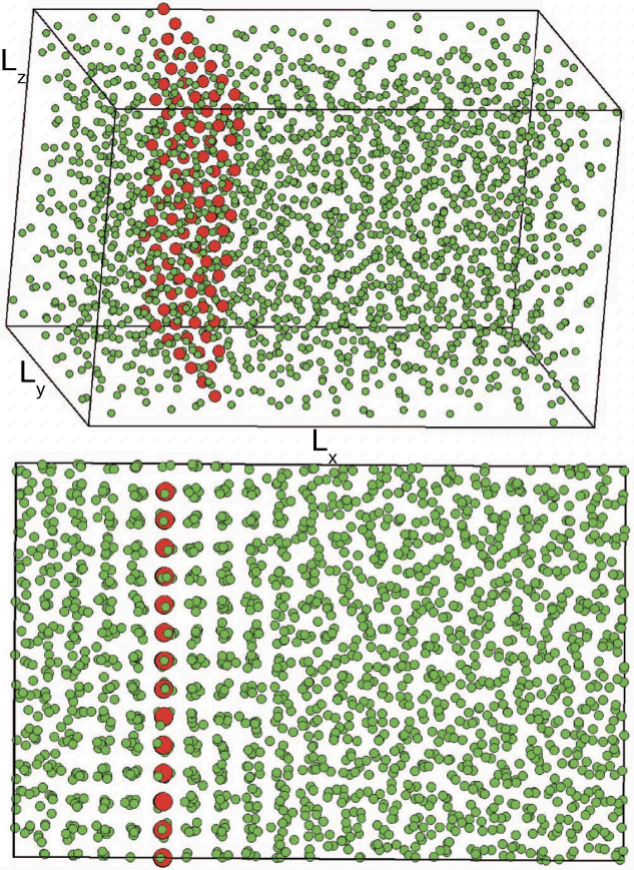
(Top) Snapshot of a hard-sphere fluid under coexistence conditions (green particles). (Bottom) Snapshot of a fluid with a thin crystal slab under coexistence conditions (a projection in the *x*–*z* plane is shown). The mold that induces the formation of the crystal slab consists of a set of potential energy wells (red spheres) whose positions are given by the lattice sites of the selected crystal plane under coexistence conditions. The interaction between the mold and the hard-spheres is switched off in the top configuration and switched on in the bottom one. The diameters of the green particles have been reduced to 1/4 of their original size. This figure was reproduced with permission from ref 228. Copyright 2014 American Institute of Physics.

Further technical details on how to evaluate *r*_*w*_^*o*^, ⟨*N*_*fw*_(ϵ)⟩, and ultimately γ_*sl*_ using the mold integration method can be found in refs 114, 228, and 229.

The mold integration method has been used to obtain the IFEs of different crystal phases (fcc and hcp in hard sphere models;^114^ hexagonal and cubic ice in water^7^). Since it can measure γ_*sl*_ directly for any crystal orientation,^228^ it has been used to distinguish different crystal orientations in Lennard-Jones systems^228^ and in the NaCl solid–melt interface.^161^ The technique has been extended to deal with more complex solid structures and coexisting liquids of different components. NaCl–water solutions have been tackled (including ice in contact with salty water^230^), along with crystalline NaCl in contact with a saturated NaCl aqueous solution at the solubility limit^137^). It has also recently been used to show the direct relation between the slope of the melting line and the pressure dependence of γ_*sl*_ for the interface between hexagonal ice and liquid water.^231^

Two important extensions of the mold integration method were developed by Algaba et al.^232^ and Zerón et al. to compute IFEs for interfaces between water and hydrates containing different guest molecules such as carbon dioxide, methane, nitrogen, hydrogen, and tetrahydrofuran.^232–234^ In the first extension, namely mold integration (host), the authors placed attractive interaction sites in the H_2_O-rich liquid phase at the equilibrium positions of the oxygen atoms of water in one of the principal planes of the sI structure of the CO_2_ hydrate.^232^ In the second extension, namely mold integration (guest), they placed attractive interaction sites at crystallographic equilibrium positions of a layer of carbon atoms of CO_2_ molecules in the CO_2_ hydrate.^233^ These clathrates and those formed from small molecules such as CH_4_, ethane, or hydrogen sulfide crystallize in the sI crystal structure. The unit cell of this structure, which exhibits cubic symmetry, is formed by 46 water molecules distributed in six T (tetrakaidecahedron or 5^12^6^2^) cages and two D (pentagonal dodecahedron or 5^12^) cages, usually denoted as “large” and “small” hydrate cages, respectively.^235,236^

Figure 5 shows two snapshots of trajectories obtained from MD simulations and used to determine the CO_2_ hydrate–water interfacial free energy using the mold integration (guest) method. The use of a mold in the mold integration (host) technique is similar to the original implementation of mold integration used for aqueous systems because the associating sites of the mold are located at the crystallographic positions of the oxygen atoms of the water molecules in the selected crystal planes. The use of both extensions of the technique for hydrates requires special attention because the coexistence conditions of the hydrate–water interface involve two different components (H_2_O and CO_2_) and three phases in equilibrium: the CO_2_ hydrate solid, the H_2_O-rich liquid, and the CO_2_-rich liquid. The presence of the three phases is necessary to ensure that calculations are performed under equilibrium coexistence conditions. It is also necessary to tune the local order parameters (See the discussion of eq 14 for a definition of the concept of an order parameter) to correctly identify hydrate-like and liquid-like water molecules in order to follow the growth of the thin hydrate layer induced by the mold. Zerón et al.^237^ recently revisited the Lechner and Dellago order parameters^238^ and have obtained a new set of order parameters that can distinguish water molecules in both phases, allowing them to correctly characterize the hydrates.

**Figure 5:**
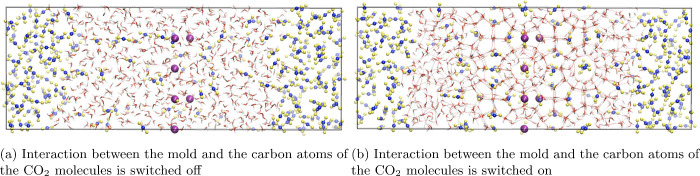
Snapshots representing trajectories extracted from molecular dynamics simulations of the CO_2_–water two-phase coexistence at 400 bar and 287 K. The mold that induces the formation of the crystal slab consists of a set of potential energy wells (magenta spheres) located at the crystallographic positions of the carbon atoms of the CO_2_ molecules of the selected crystal planes at coexistence conditions. The red and white licorice representation corresponds to oxygen and hydrogen atoms of water, respectively; blue and yellow spheres (van der Waals representation) correspond to carbon and oxygen atoms of CO_2_, respectively.

Although both methods (host and guest) are based on the mold integration technique, they require rather different calculations. In the host case,^232^ a mold of associating wells is placed in the crystallographic equilibrium locations occupied by the oxygen atoms of water molecules in the primary plane of the sI hydrate. However, in the guest case^233^ a mold of associating wells is located at the centers of the T and D cages in the sI hydrate structure, corresponding to the equilibrium positions of the carbon atoms of CO_2_ molecules in two different planes. The type, number, and well-depth of the associating wells are also different. The results can therefore be regarded as arising from two distinct approaches. The group has also determined the CO_2_ hydrate–water interfacial energy along the dissociation line of the hydrate at several pressures (100, 400, and 1000 bar).^234^ The results show a weak correlation between interfacial free energy values and pressure, with γ_*sx*_ decreasing with pressure. Unfortunately, this prediction cannot be compared with literature experimental data, since the latter assumes that the interfacial energies are independent of the pressure. We present a more detailed discussion on the hydrates, their structures, and the challenges in obtaining the value of the interfacial properties in section 7.2.

### 5.3 Einstein Crystal Method

This is a relatively recent method developed independently by Addula and Punnathanam^227^ and Yeandel et al.^239^ The key idea is to avoid an explicit real-space transformation of a bulk material into an interface by using a reference state to which both bulk and interfacial systems can be easily transformed. The chosen reference state is the Einstein crystal,^240,241^ which comprises noninteracting atoms confined to individual harmonic potential wells. The primary benefit of using the Einstein crystal as a reference state is that the real-space position of the harmonic potential does not affect the total free energy of the Einstein crystal, and therefore the thermodynamic work required to rearrange an Einstein crystal in real-space is zero. Exploiting this property allows for the construction of an interfacial system from bulk material without having to identify how the atoms must rearrange to achieve the transformation.

The usual approach used in the Einstein crystal method is to generate a liquid–vacuum interface and then replace the vacuum component of the interface with a solid component using an Einstein crystal (see Figure 6). The stages required for the calculation are

1. Compute the free energy required to generate a vacuum gap in the bulk liquid (Δ*F*_Liquid_^Liquid+Vacuum^).
2. Prepare a bulk solid system and transform it into an Einstein crystal, with the work required for the transformation recorded (Δ*F*_Bulk_^Ein.^).
3. Prepare a liquid–solid–liquid “slab” system with the desired interfacial configuration (crystal orientation/cutting plane/reconstruction) and transform the solid component of this system into an Einstein crystal, recording the work needed (Δ*F*_Slab_^Ein.^).

**Figure 6:**
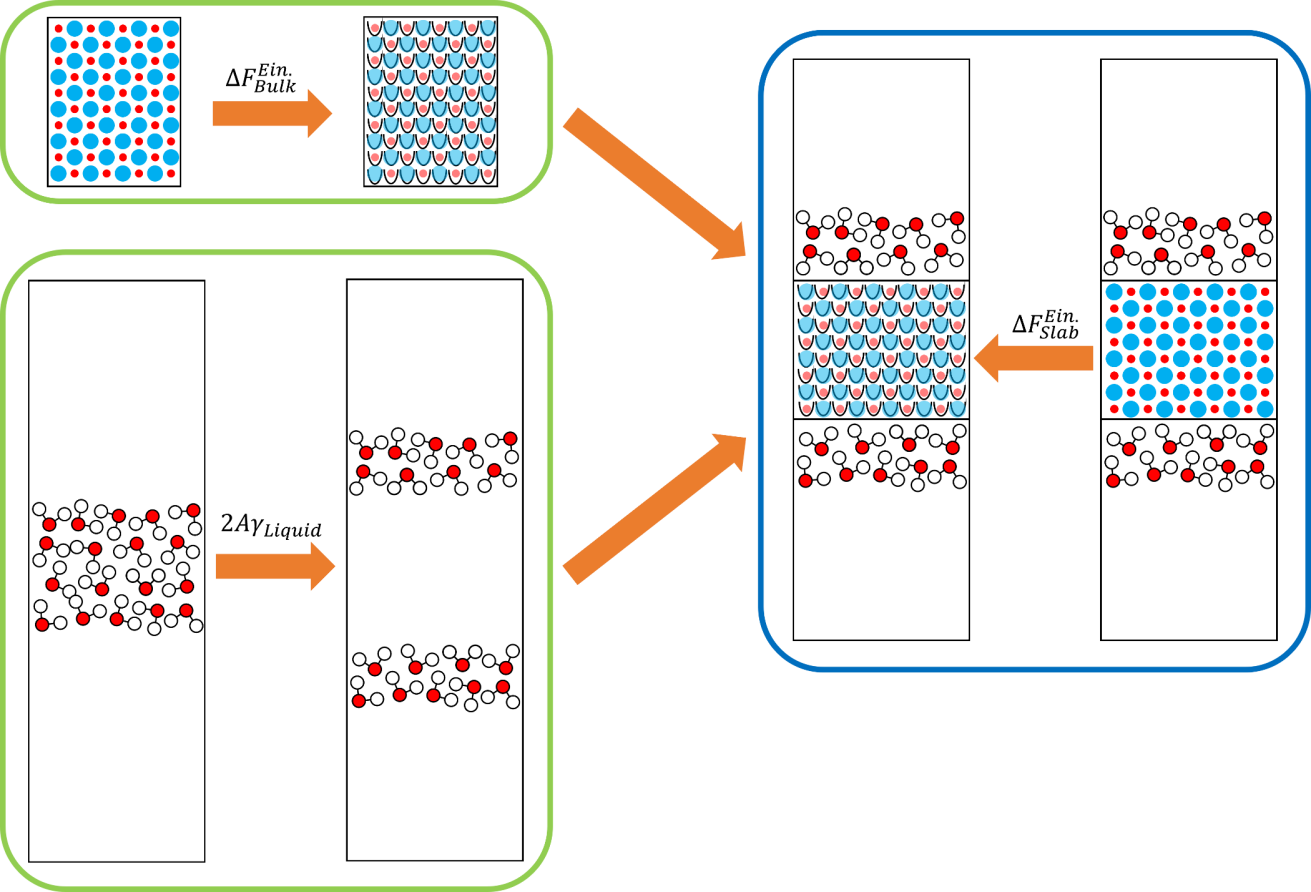
(Top left) Schematic of the transformation of bulk solid material into an Einstein crystal. (Bottom left) Schematic of the creation of a vacuum gap in a liquid. (Right) Schematic of the transformation of solid in the slab system into an Einstein crystal. Processes in green boxes need only be performed once and the free energy scaled to match the slab system. The process in the blue box is repeated for each slab system. This figure was reproduced from ref 239 under a CC BY 4.0 license.

The value of Δ*F*_Liquid_^Liquid+Vacuum^ computed in Step 1 is equivalent to creating two liquid/vacuum interfaces of total area A . As this is the free energy of creating a fluid/fluid interface, we may use the Shuttleworth equation, eq 6, and identify Δ*F*_Liquid_^Liquid+Vacuum^ with the appropriately scaled surface tension ( $\Delta F_{\mathrm{Liquid}}^{\mathrm{Liquid}+\mathrm{Vacuum}}=A\gamma_{l}$ ). The value of γ_*l*_ can then be efficiently computed using the method of Kirkwood and Buff^140^ (see eq 8) and reused for every calculation with the same liquid phase. The values of Δ*F*_Bulk_^Ein.^ and Δ*F*_Slab_^Ein.^ required by Steps 2 and 3 may be computed using any applicable methods (e.g., TI^214^ or Bennett acceptance ratio^242^). The values of Δ*F*_Bulk_^Ein.^ may be computed once for a given bulk solid and scaled for use with multiple different slab systems (surface configurations). By contrast, the value of Δ*F*_Slab_^Ein.^ must be computed for each interface of interest.

Although in principle many different approaches may be used to transform the solid material into an Einstein crystal, published studies have thus far opted to use TI^227,239^ (see appendix A). There are a number of different ways in which TI can be used to transform the solid into an Einstein crystal. In general, a two-stage approach is preferred in which the first stage is used to “switch on” the harmonic potential, and then a second stage is used to “switch off” all solid–solid and solid–liquid interactions. This choice restricts atoms from approaching too closely as interactions are “switched off”, which could otherwise lead to instability. Additional TI stages may also be included to further ensure stability of the transformation, such as using a “cleaving wall” type approach to first separate the solid and liquid components of the interfacial system before transformation to an Einstein crystal. Throughout the transformation to an Einstein crystal, the positions of the harmonic potentials must be kept stationary to ensure consistency in the TI procedure. The IFE is then computed using the equation

$$\gamma_{sx}=\frac{\Delta F_{\mathrm{Liquid}}^{\mathrm{Liquid}+\mathrm{Vacuum}}+\Delta F_{\mathrm{Bulk}}^{\mathrm{Ein}.}-\Delta F_{\mathrm{Slab}}^{\mathrm{Ein}.}}{A}=\gamma_{l}+\frac{\Delta F_{\mathrm{Bulk}}^{\mathrm{Ein}.}-\Delta F_{\mathrm{Slab}}^{\mathrm{Ein}.}}{A}$$ 34

where Δ*F*_Bulk_^Ein.^ has been scaled to match the stoichiometry of the “slab” system.

A key benefit of the Einstein crystal method is the ability to study specific surface configurations, which may not be directly accessible by cleaving or other simple real-space transformations (e.g., stepped surfaces, surface patterning, etc.). The use of the Einstein crystal as a reference state also allows efficient reuse of previous calculations because only a single transformation of the bulk solid needs to be calculated for many different surface configurations and/or liquid phases. The work of adhesion is also accessible by computing the transformation of the dry surface into an Einstein crystal (replacing the transformation of the bulk solid into an Einstein crystal, Δ*F*_Bulk_^Ein.^) and discarding the creation of the liquid surface from the bulk liquid, γ_*l*_, in eq 34. Another advantage of the Einstein crystal approach is that a vacuum gap may be added around the interfacial system. The use of this additional vacuum gap is that dipole corrections^243,244^ may be added to obtain consistent energies for dipolar surfaces. The additional vacuum gap and corresponding liquid/vacuum interfaces remain in place throughout the entire calculation, and so no additional correction is required for the computed interfacial free energy.

The Einstein crystal method is most appropriate for systems in which the bulk material has low solubility in the liquid phase. In highly soluble systems, or near the coexistence point, difficulties arise in defining which atoms belong to the solid and which to the liquid. Constraining an atom in the fluid state to a harmonic potential leads to a divergence in the TI procedure and the free energy is poorly defined. In such cases, other methods described in this section should be preferred. When the solid phase contains species that are miscible in the liquid phase (e.g., water in hydrous clays), corrections can be applied to the Einstein crystal method to obtain a consistent IFE.^239^ Although a relatively new approach, the Einstein crystal method has already been applied to a diverse set of interfaces including orcinol–chloroform and orcinol–nitromethane,^227^ NaCl–water and CaSO_4_·*x*H_2_O–water,^239^ and CaCO_3_–water.^245^

### 5.4 Phantom Wall Method

The phantom wall method^246,247^ takes its name from the fact that the liquid is separated from the solid by using a wall (for a slab configuration there will be two walls) described by an external potential that interacts only with liquid atoms and is completely transparent to solid atoms (hence the name “phantom wall”). γ_*sl*_ is determined by calculating the difference in the Gibbs free energy between a configuration in which the liquid is in contact with the solid and a reference configuration in which the liquid is in contact with the walls acting as an external potential. The thermodynamic path starts with the walls buried within the solid, sufficiently far away from the liquid to avoid any interactions with it. The walls are then moved in the direction perpendicular to the solid–liquid interface, pushing the liquid away from the solid interface. During this path, the volume of the system changes, and therefore this contribution needs to be taken into account in the calculation of the difference in Gibbs free energy. With this method, the Gibbs free energy change per unit area, Δγ^*PW*^, is given by^248^

$$\Delta \gamma^{PW}=\gamma_{wl}+\gamma_{s}-\gamma_{sl}+P_{N}\frac{\Delta V}{A}$$ 35

where *P*_*N*_ is the component of the pressure tensor in the direction normal to the interface (as in eq 8) and ΔV is the change in the system volume after the transformation. γ_*wl*_ is the wall-liquid interfacial tension, whereas γ_*s*_ is the IFE of the solid in contact with vacuum. γ_*wl*_ can be calculated through the mechanical route (eq 8),^140^) but the term γ_*s*_ needs to be determined from its thermodynamic definition. If the value of γ_*s*_ is not available, the methodology can only determine the work of adhesion (per unit of surface) between the solid and the liquid, *W*_*sl*_ = γ_*lv*_ + γ_*s*_ – γ_*sl*_,^249,250^ or the heat of immersion, defined as γ_*sl*_ – γ_*s*_, provided that γ_*s*_, the IFE of a solid in contact with vacuum, and γ_*sv*_, the IFE of a solid–vapor interface, can be considered equal. The latter assumption is approximately correct for surfaces with weak interactions with the fluid.^251^ The only term remaining in eq 35, Δγ^*PW*^, has to be obtained through TI.

The phantom-wall method was used to study a Lennard-Jones liquid in contact with its solid,^247^ water in contact with rugged graphite,^252^ and water in contact with α-quartz surfaces coated with perfluoro-dimethylsilanes.^253^ The interest in systems with rough or smooth interfaces stems from the fact that the roughness at the nanoscale can modify the hydrophobicity of an interface.^254^ Other applications involve the determination of the contact angle of a water–graphene system.^255^

### 5.5 Dry-Surface Method

Here we briefly discuss the dry-surface method developed by Leroy and Müller-Plathe,^248^ even though it was developed primarily to calculate the work of adhesion, *W*_*sl*_,^249,250^ between a solid and a liquid phase in contact. In the dry-surface method the quantity *W*_*sl*_ is obtained by modifying the depth of the well of the solid–liquid interaction potential, turning it into a purely repulsive interaction. The dry-surface method was used to determine the interfacial thermal resistance, which was then used to calculate the evaporation rate of droplets on a heated surface.^256^ The method was extended to three-phase systems in refs 158 and 257 to calculate the work of adhesion of a droplet to a surface. In this work, a liquid droplet was detached from a solid surface which is also in contact with vapor. In refs 257 and 258, the authors used the work of adhesion determined by this method to obtain the contact angle between the droplet and the solid surface predicted by Young’s equation and compare it with the one observed in a three-phase system (droplet on a solid surface in contact with vapor). In ref 158, the authors determined the work of adhesion in order to calculate the line tension of a liquid droplet in contact with a solid surface. The line tension is the locus of the intersection of the three phases, the droplet, the surface, and the vapor, and although it is a concept known since the time of Gibbs, there is no satisfactory description of its behavior in terms of the physical parameters of the system.^259^ As noted in ref 158, the use of the dry-surface method to calculate the work of adhesion for a droplet system may not give a reversible path, essential if TI is used to obtain the equilibrium value. Even if, as noticed in the same article, the error in the final value of the work (calculated by using different initial configurations) is small, one should be aware of these issues. For models using TI, the absence of hysteresis should always be carefully checked before extracting physical information from the simulations.

### 5.6 Other Methods

In addition to the methods discussed earlier, this section highlights a number of alternative approaches that, while less widely used, offer unique perspectives and potential applications in specific contexts.

The first example is the test area method,^260^ which estimates the IFE by calculating the free energy difference between two states with different interfacial areas at constant volume from the ratio of configurational phase-space integrals for isothermal perturbations

$$\gamma ={\left(\frac{\partial F}{\partial A}\right)}_{NVT}=\underset{\Delta A\to 0}{\lim}-\frac{k_{B}T}{\Delta A}\ln\langle \exp\left(-\frac{\Delta U}{k_{B}T}\right)\rangle_{NVT}$$ 36

where ΔA and Δ*U* are the differences in interfacial area and configurational energy between the perturbed and reference systems. The method can be used in the canonical,^260^ isothermal–isobaric,^261^ and grand canonical ensembles,^262^ and it has been applied to calculate the IFEs of vapor–liquid and fluid–fluid interfaces of many different systems, ranging from simple models,^263–269^ to more realistic molecular systems.^270–279^ It has been extended to include the calculation of fluid–fluid IFEs in different geometries.^280–283^ For a more detailed account of the work devoted to determining the IFE of fluid–fluid interfaces, we recommend the review of Ghoufi et al.^261^ For what concerns solid–liquid interfaces, the test area method has been used to estimate the IFE,^284–286^ but the solid walls are treated at the level of an external potential (and further work is needed to validate this approach). In any case, the method cannot be used to determine the value of γ between a fluid and its solid phase at coexistence. In addition, since the Test Area method can be regarded as a route to determine the components of the pressure tensor using small volume perturbations, it is also affected by the failure of the mechanical route for determining γ for fluid–solid interfaces, as discussed in section 3.2.

The interfacial free energy can also be estimated by using metadynamics, which is a biasing technique that makes it possible to efficiently reconstruct the free energy surface of a system in terms of collective variables^287^ (see refs 288–291 and references therein for details). The idea, introduced by Angioletti-Uberti et al.^292^ for a Lennard-Jones system using the Broughton–Gilmer potential, is to use metadynamics to reconstruct the free energy surface of a system transitioning from a single solid or liquid phase to coexistence using a local order parameter that distinguishes between the two phases as the collective variable. The difference in Gibbs free energy between these two regions at the solid–liquid equilibrium temperature is then proportional to the IFE.

In the context of energy applications, a method based on thermodynamic integration was recently developed to evaluate free energy differences associated with changes in the Thomas–Fermi screening length, making it possible to efficiently compute the interfacial free energies in systems with varying metallicity.^293^ Its applicability to both empty capacitors and electrochemical cells makes it a versatile tool for exploring substrate-dependent phenomena.

Another route for the determination of the interfacial tension is provided by the bias successive umbrella sampling (BSUS) technique, which has been applied for the first time to liquid–solid interfacial systems in a patchy particle model.^37^ By running grand canonical Monte Carlo simulations across overlapping density windows, BSUS reconstructs the probability distribution along the reaction coordinate. By reweighting the resulting distribution so that the liquid and solid phases are at coexistence, it is possible to obtain the free-energy cost of forming the interface, which can be divided by the interfacial area to yield γ_*sl*_.

### 5.7 Gibbs–Cahn Integration

We have left this part to the very end of this section, as the Gibbs–Cahn integration is not, strictly speaking, a technique to calculate γ_*sl*_ but rather a way to determine how γ_*sl*_ varies with respect to thermodynamic conditions, such as pressure, temperature, and composition. All of the methodologies presented above give γ_*sl*_ for a single thermodynamic point along the solid–liquid phase boundary. Finding the IFE at other thermodynamic coexistence conditions requires repeating the calculations for the new thermodynamic point (whichever approach is used). Gibbs–Cahn integration instead allows one to obtain simple rules to derive a range of values for γ_*sl*_ (for different thermodynamic conditions) from knowing at least one of its values on the thermodynamic coexistence path.

The Gibbs–Cahn integration technique is based on Cahn’s reformulation of the surface thermodynamics of Gibbs^119^ (reprinted in ref 88). In Cahn’s formulation, the excess quantities of the interface are now expressed in the form of determinants of matrices whose entries are the extensive properties of the interfacial and bulk systems, making it possible to establish a connection between the differential of the IFE and the properties of the system directly measurable in simulations. Once these properties have been computed numerically, the IFE is obtained by integrating the differential over a parameter of choice (similar in spirit to the well-known Gibbs–Duhem integration^294,295^ used to determine the phase coexistence line). We will now introduce the most important features of this methodology. We believe that the Cahn model is as important in the treatment of interfaces as the Gibbs model, and we therefore discuss it in detail. Assuming a *c*-component system containing an interface, we can write the total Gibbs energy as^296^

G=E−TS+PV 37

For a bulk system without an interface with *c* components, the Gibbs energy is given by

$$G_{b}=\overset{c}{\underset{k=1}{\sum }}\mu_{k}N_{k}$$ 38

where μ_*k*_ is the chemical potential of particles of type *k*. The interfacial free energy, γ, is given by the difference (per unit area) between the Gibbs energy of the system including the interface and that of the coexisting bulk phases (solid and liquid):

$$\gamma A=G-G_{b}=E-TS+PV-\overset{c}{\underset{k=1}{\sum }}\mu_{k}N_{k}$$ 39

where we are assuming that the solid phase is under hydrostatic stress. Taking the differential of this quantity gives

$$d\left(\gamma A\right)=dE-TdS-SdT+PdV+VdP-\overset{c}{\underset{k=1}{\sum }}\mu_{k}dN_{k}-\overset{c}{\underset{k=1}{\sum }}N_{k}d\mu_{k}$$ 40

For a system containing a planar interface where one of the coexisting phases is a crystalline solid, the differential for the energy, still assuming hydrostatic conditions in the crystal, is given by^297^

$$dE=TdS-PdV+\underset{i,j=1,2}{\sum }\left(\psi_{ij}+\delta_{ij}P\right)Vdu_{ij}+\overset{c}{\underset{k=1}{\sum }}\mu_{k}dN_{k}$$ 41

where ψ_*ij*_ and *u*_*ij*_ are the *ij* components of the stress and strain tensors, respectively, and *i* and *j* are elements of the set {1, 2}, which represent the transverse Cartesian directions. Substituting eq 41 into eq 40 yields

$$d\left(\gamma A\right)=-SdT+VdP+\underset{i,j=1,2}{\sum }\left[\left(\psi_{ij}+\delta_{ij}P\right)V\right]du_{ij}-\overset{c}{\underset{k=1}{\sum }}N_{k}d\mu_{k}$$ 42

For a solid–liquid interface, in addition to eq 42, we have the two Gibbs–Duhem equations for the hydrostatic bulk solid and bulk liquid:

$$0=-S_{s}dT+V_{s}dP-\overset{c}{\underset{k=1}{\sum }}N_{k,s}d\mu_{k}$$ 43

and

$$0=-S_{l}dT+V_{l}dP-\overset{c}{\underset{k=1}{\sum }}N_{k,l}d\mu_{k}$$ 44

where the subscripts *s* and *l* denote properties of the bulk solid and liquid, respectively. For the set of three simultaneous linear eqs (eqs 42–44), Cahn used Cramer’s rule to eliminate any selected pair of differentials d*x* and d*y* (e.g., d*P* and d*N*_*k*_) to give

$$d\left(\gamma A\right)=-\left[S/XY\right]dT+\left[V/XY\right]dP+\underset{i,j=1,2}{\sum }\left[\left(\psi_{ij}+\delta_{ij}P\right)V/XY\right]du_{ij}-\overset{c}{\underset{k=1}{\sum }}\left[N_{k}/XY\right]d\mu_{k}$$ 45

where *X* and *Y* are the variables conjugate to the displacements d*x* and d*y*, and the notation [*R*/*XY*] (where *R* is a generic thermodynamic extensive variable) is defined as

$$\left[R/XY\right]=\frac{\left|\begin{array}{ccc} R & X & Y\\ R_{l} & X_{l} & Y_{l}\\ R_{s} & X_{s} & Y_{s} \end{array}\right|}{\left|\begin{array}{cc} X_{l} & Y_{l}\\ X_{s} & Y_{s} \end{array}\right|}$$ 46

where quantities without subscripts refer to the entire system (solid + liquid + interface). We are now ready to provide the explanation promised in section 3.1 about the relation between eq 3 and eq 4. For a single-component system (*c* = 1), a common choice is X = *N* and *Y* = *V*, which is equivalent to choosing a Gibbs dividing surface (i.e., no excess volume) in which the excess number of particles is zero ([*N*] = 0) (Note that this is a special case, as Cahn’s approach is more general than Gibbs’: if neither *X* nor *Y* are chosen to be *V*, then there is a nonzero excess volume, a choice that goes beyond the usual Gibbs dividing surface concept). With this choice, the d*P* and dμ terms in eq 45 are both zero, since two columns in the determinant of eq 46 are identical. Applying this choice gives

$$d\left(\gamma A\right)=-\left[S/NV\right]dT+\underset{i,j=1,2}{\sum }\left[\left(\psi_{ij}+\delta_{ij}P\right)V/NV\right]du_{ij}$$ 47

where the determinant [*S*/*NV*] reduces to the total excess entropy *S* ^*XS*^, with excess quantities defined as in eq 2. Because we assume that the system is hydrostatic, and the stress in the bulk is zero, the second term on the right-hand side of eq 47 can be obtained as follows:

$$\left[\left(\psi_{ij}+\delta_{ij}P\right)V/NV\right]=\frac{\left|\begin{array}{lll} \left(\psi_{ij}+\delta_{ij}P\right)V & N & V\\ 0 & N_{s} & V_{s}\\ 0 & N_{l} & V_{l} \end{array}\right|}{\left|\begin{array}{cc} N_{s} & V_{s}\\ N_{l} & V_{l} \end{array}\right|}=\left(\psi_{ij}+\delta_{ij}P\right)V$$ 48

Note that the quantity $\left(\psi_{ij}+\delta_{ij}P\right)V$ is an excess surface quantity. However, in this case the total stress and the excess stress are the same (because of our assumption of zero stress in the bulk), and there is no need to overburden the notation.

For simplicity, it is useful to restrict the discussion to high-symmetry interface orientations where ψ_12_ = ψ_21_ = 0, but the extension to lower symmetry crystal structures or orientations is straightforward. Mechanical equilibrium at the interface guarantees that ψ_33_ = – *P*_*zz*_ = −*P* at the interface, yielding the following (after dividing by A ):

$$\frac{1}{A}d\left(\gamma A\right)=-\left[S\right]dT+\left(\psi_{11}+P\right)Vdu_{11}+\left(\psi_{22}+P\right)Vdu_{22}$$ 49

The strain can be related to the change in the interfacial area because the crystal expands as one moves along the coexistence curve:

$$du_{11}=du_{22}=\frac{dA}{2A}$$ 50

so that eq 49 becomes

$$\frac{1}{A}d\left(\gamma A\right)=-\left[S\right]dT+\left(\psi_{11}+\psi_{22}+2P\right)V\frac{dA}{2A}=-\left[S\right]dT+f\frac{dA}{A}$$ 51

where *f* is the average excess interfacial stress defined as *f* = $f=\frac{\left(f_{xx}+f_{yy}\right)}{2}$ with *f*_*xx*_ = ∫ _–*∞*_^+*∞*^(*P* – *P*_*xx*_)­d*z* and *f*_*yy*_ = ∫ _–*∞*_^+*∞*^(*P* – *P*_*yy*_)­d*z* (see ref 117). From this definition and eq 51 we obtain:

$$f=\frac{1}{A}\left(\frac{\psi_{11}+\psi_{22}}{2}+P\right)V=\int_{-\infty }^{\infty }\left[P_{zz}-\frac{P_{xx}+P_{yy}}{2}\right]dz$$ 52

where *P*_*zz*_ and (*P*_*xx*_ + *P*_*yy*_)/2 are the pressure components normal and transverse to the interface, respectively (compare section 3.2 eq 8). Note that we do not indicate the surface excess quantity per unit of area in eq 52 using the brackets as we have a specific symbol for it. The use of eq 51 requires knowledge of the excess interfacial entropy, [*S*], which is not readily available from the simulations. To remedy this, Frolov and Mishin^297^ in their work on surface free energy and Baidakov et al.^298^ in the context of liquid–vapor interfaces combine the equation γ = [*E*] – *T*[*S*] (Note that this equation is given as γ = *e* – *Tη* in ref 118) with the fact that $\left[S\right]=-{\left(d\gamma /dT\right)}_{A}$ (from eq 51) to derive

$$\frac{1}{A}d\left(\gamma A/T\right)=-\frac{\left[E\right]}{T^{2}}dT+\frac{f}{T}\frac{dA}{A}$$ 53

which relates changes in γ to the more easily obtainable excess interfacial energy per unit area, [*E*], by analogy with the familiar Gibbs–Helmholtz equation in thermodynamics. Dividing both sides of eq 53 by d*T* along the coexistence curve and using the fact that the interfacial area, A , is proportional to ρ_*s*_^–2/3^ for high symmetry crystals, where ρ_*s*_ is the number density of the solid, we obtain

$${\left[\frac{d\left(\gamma_{g}/T\right)}{dT}\right]}_{\mathrm{coex}}=-{\rho_{s}}^{-2/3}\left[\frac{\left[E\right]}{T^{2}}+\frac{2f}{3\rho_{s}T}{\left(\frac{d\rho_{s}}{dT}\right)}_{\mathrm{coex}}\right]$$ 54

Here γ_*g*_ = ρ_*s*_^–2/3^γ is the “gram-atomic” IFE per surface atom defined by Turnbull.^106^ Given a value of γ at a reference point on the coexistence curve determined by one of the methods discussed in this section, eq 54 can be integrated along the coexistence curve to calculate γ at any other point on the curve using the values of [*E*] and *f*, which are easily calculated from a single simulation. This process is far less computationally expensive than the many simulations required to perform a full γ calculation at each temperature using direct methods.

Gibbs–Cahn integration has been successfully applied to solid–vapor and solid–liquid IFEs of metals and metal alloys,^124,297^ to Lennard-Jones systems,^118^ to investigate the dependence across the coexistence line of the liquid–vapor and liquid–solid IFEs of Lennard-Jones particles and atomistic and coarse-grained models of water.^145^ Frolov and Mishin later extended the formalism to include the effect of nonhydrostatic stress on the solid–fluid interfaces.^299,300^ For systems in which the solid is modeled as a static surface (such as a hard-sphere fluid at a structureless hard wall), the application of the Gibbs–Cahn formalism is simplified by the fact that there is only one Gibbs–Duhem equation and the matrices describing the excess quantities are 2 × 2 only. This modification has allowed the calculation of the IFE for the hard-sphere (3D) and hard-disk (2D) fluids at planar hard walls. The method has also been extended to hard-core fluids at curved interfaces in both two and three dimensions.^301–304^ Analysis of the case of a hard-disk fluid inside a circular hard wall (container)^304^ requires a reformulation of the Gibbs–Cahn formalism within the grand canonical distribution.

## 6 Interfacial Solid–Liquid Free Energy for Benchmarked Systems

In the previous section we provided an account of the different methods available for the calculation of interfacial properties, specifically for systems involving a solid. In the same section, we included many examples where such approaches have been applied. However, there are some systems that occupy a privileged position in the development of the methods presented here. These systems are usually characterized by simple interaction potentials so that they do not show the complications that can often be found when dealing with complex molecules and molecular crystals. This simplifies the development of the methodologies (e.g., the determination of a thermodynamic path for thermodynamic integration), yet they are also general enough to mimic physicochemical properties of real systems. These *benchmark systems*, which comprise the hard-sphere and Lennard-Jones models discussed in this section, are usually the first considered in any development of a new methodology, which is why we discuss them in more depth. For convenience, this section (unlike the others) uses reduced Lennard-Jones units throughout.

### 6.1 Hard Spheres

#### 6.1.1 Hard-Sphere Crystal–Melt Interface

The hard-sphere model has been extensively used to benchmark different computational approaches designed to evaluate solid–melt interfacial free energies. The first calculation for this system was performed in 2000 by Davidchack and Laird.^218^ Later, alternative methods such as capillary wave fluctuations,^193,205^ nonequilibrium capillary simulations,^115^ tethered Monte Carlo,^305^ thermodynamic integration,^306^ mold integration,^114,228^ and ensemble switch^307^ have been used to estimate γ_*sl*_ for different crystal planes of the fcc crystal phase in hard spheres. Additionally, the analysis of free energy barriers based on classical nucleation theory^165^ has provided estimates of γ_*sl*_ as a function of supersaturation and under coexistence conditions by data extrapolation.^114,186,191^ In Table 1, we summarize all the known (to us) published values of γ_*sl*_ for different crystal orientations of the fcc and hcp phases, as well as the average values of γ_*sl*_ (*γ̅*_*sl*_) from crystal nucleation studies. A similar table was reported in ref 308, where the authors also included the interfacial stiffness for the hard-sphere case, along with the IFE.

**Table 1:** Solid–Melt Interfacial Free Energy (*γ*_*sl*_ in *k*_B_ *T*/*σ* ^2^) of the Hard-Sphere fcc and hcp Phases for Different Crystal Orientations as Indicated by the Miller Indexes^a^

γ_*sl*_ fcc	technique	(100)	(110)	(111)	(120)	average γ_*sl*_
Davidchack and Laird 2000 ^218^	CW	0.62(1)^a^	0.62(1)^a^	0.58(1)^a^		
Cacciuto et al. 2003 ^191^	CNT					0.616(3)
Mu et al. 2005 ^309^	CF	0.64(2)	0.62(2)	0.61(2)		
Davidchack et al. 2006 ^193^	CF	0.574(17)	0.557(17)	0.546(16)		
Davidchack 2010 ^115^	CF	0.582(2)	0.559(2)	0.542(3)	0.567(2)	
Fernandez et al. 2012 ^305^	TMC	0.636(11)				
Hartel et al. 2012 ^207^	CF	0.639(1)	0.600(1)	0.600(1)		
Benjamin and Horbach 2015 ^306^	TI	0.596(6)	0.577(4)	0.556(3)		
Schmitz and Virnau 2015 ^307^	ES	0.581(3)	0.559(1)	0.544(8)		
Espinosa et al. 2016 ^186^	CNT					0.58(3)
Bültman and Schilling 2020 ^310^	TI	0.591(11)				
Sanchez-Burgos et al. 2021 ^114^	MI/CNT	0.586(6)	0.572(7)	0.554(6)		0.57(1)

γ_*sl*_ hcp	technique	(112̅0)	(101̅0)	(0001)	average γ_*sl*_
Sanchez-Burgos et al. 2021 ^114^	MI/CNT	0.597(6)	0.586(6)	0.554(6)	0.59(1)

a In the last column we report the averaged values of *γ*_*sl*_ obtained from nucleation studies. Various computational approaches have been employed for the determination of *γ*_*sl*_: cleaving wall [CW], capillary wave fluctuations [CF], mold integration [MI], tethered Monte Carlo [TMC], ensemble switch [ES], thermodynamic integration [TI], and classical nucleation theory analysis of nucleation free energy barriers [CNT]. Numbers in parentheses indicate the estimated error on the last digit(s) shown.

b These results contain a systematic error, which was later corrected in ref 115.

As can be seen, most of the calculations of γ_*sl*_ for the fcc phase show that the relative values of IFE as a function of the crystal planes considered are γ_*sl*_(100) > γ_*sl*_(110) > γ_*sl*_(111). Some of the first direct calculations^207,218,305,309^ predicted slightly higher values of γ_*sl*_ for these three planes than those from refs 114, 115, 193, 306, 307, and 310, with values ranging from 0.60 to 0.64 *k*_B_ *T*/σ^2^ depending on the technique and the crystal plane. More recent calculations have predicted slightly lower values: approximately 0.58–0.59 *k*_B_ *T*/σ^2^ for the (100) plane, 0.56 *k*_B_ *T*/σ^2^ for the (110) plane, and 0.54–0.55 *k*_B_ *T*/σ^2^ for the (111) plane, reaching a consensus through different computational techniques.^114,115,193,306,307,310^ In addition, predictions from nucleation studies using the CNT framework^114,186,191^ also agree relatively well with direct calculations of γ_*sl*_ under coexistence conditions, with values ranging from 0.57 to 0.61 *k*_B_ *T*/σ^2^, as shown in Table 1.

Only ref 114 provides values for two additional crystal orientations of the hcp phase (because the (0001) orientation in the hcp and (111) plane of the fcc phase are equivalent). It is unclear which of these two phases would have a lower overall IFE given the small number of crystal orientations studied. However, in ref 114 the authors used seeding calculations to estimate the average γ_*sl*_ for fcc and hcp crystal clusters of different sizes (ranging from 300 to 95000 atoms). The values of γ_*sl*_ obtained from these calculations seem to support the notion that the overall γ_*sl*_ for the hcp phase is slightly higher than that for the fcc crystal. Nevertheless, the differences are within the uncertainty of the calculations for most of the clusters. Therefore, if hcp crystals indeed show slightly higher IFEs than fcc ones, the difference is likely to be minimal.

Extending the work on this potential to multicomponent systems, the IFE for a two-component (binary) mixture of hard-spheres with a diameter ratio of 0.9 was calculated by Amini and Laird^206^ using the capillary fluctuation method. This diameter ratio was chosen because an accurate phase diagram for this system had been previously calculated by Kranendonk and Frenkel^311^ and the structure and dynamics of this system had been studied in detail by Davidchack and Laird,^312^ which established the protocols for constructing an equilibrium binary interface in MD simulation.

#### 6.1.2 Hard-Sphere Fluid at Structureless Hard Walls

The hard-sphere fluid at static structureless hard walls is a benchmark system for the generic understanding of inhomogeneous fluids and solid–liquid interfaces and for the testing of related theoretical techniques, such as classical density functional theory. A “static” wall is one that is rigid and nonelastic and can be either patterned (with fixed hard spheres arranged in a regular pattern, for instance) or structureless; in other words, the wall acts as an unchanging external field as opposed to being a dynamic coexisting solid. The first calculation of the IFE for a hard sphere fluid at a planar hard wall from atomistic simulation came from Henderson and van Swol,^313^ who used the mechanical route (see eq 8 discussed in section 3.2). We note here that usual failure of the mechanical route for solid–liquid interfaces does not apply in this system because the solid phase is static and not an atomistic elastic solid. This method is numerically challenging because the calculation of the difference between two pressures has a high statistical error and for the highest density studied, 0.901 σ^–3^, the IFE value is measured to be 1.8(6) *k*_B_ *T*/σ^2^ (The value actually reported was negative because the authors used a definition of the wall position that ignored the contribution of the external field to the value of γ, which is given by *Pσ*/2, where *P* is the pressure. In general, when comparing calculations for interfaces with static solids, it is important to note the definition of wall position used). Later application of the mechanical Kirkwood–Buff equation, eq 8, by de Miguel and Jackson^314^ still exhibited a significant statistical error, although it was much improved over the earlier calculation. Heni and Löwe,^315^ followed later by Fortini and Dijkstra,^316^ calculated γ for this system using thermodynamic integration. These calculations also included evaluations of the hard sphere crystal/hard wall IFE, which together with the hard sphere crystal–melt IFE determined earlier, can be used to see whether the hard sphere crystal will wet the hard wall to validate simulation evidence for hard sphere surface prefreezing. The results showed clear evidence of partial wetting at the (100) and (110) surfaces but were not sufficiently precise to determine if prefreezing (complete wetting) could occur for the (111) surface.

To increase the precision of the hard sphere/hard wall calculation, Laird and Davidchack^317^ adapted the cleaving method to determine the wall–fluid and wall–crystal IFE for the full range of fluid pressures and demonstrated that the (111) crystal exhibits complete wetting at the surface in the presence of the fluid for densities at and just below the freezing transition. They later repeated the calculation of the wall–fluid IFE using Gibbs–Cahn integration^318^ to show that this method can be used to determine the IFE over the entire fluid range with a computational effort lower than that required to determine γ for a single density by other methods. The results for γ for the hard sphere fluid at a planar hard wall for several methods are shown in Figure 7. The Gibbs–Cahn integration method has also been applied to the binary hard-sphere fluid at a planar hard wall.^319^

**Figure 7:**
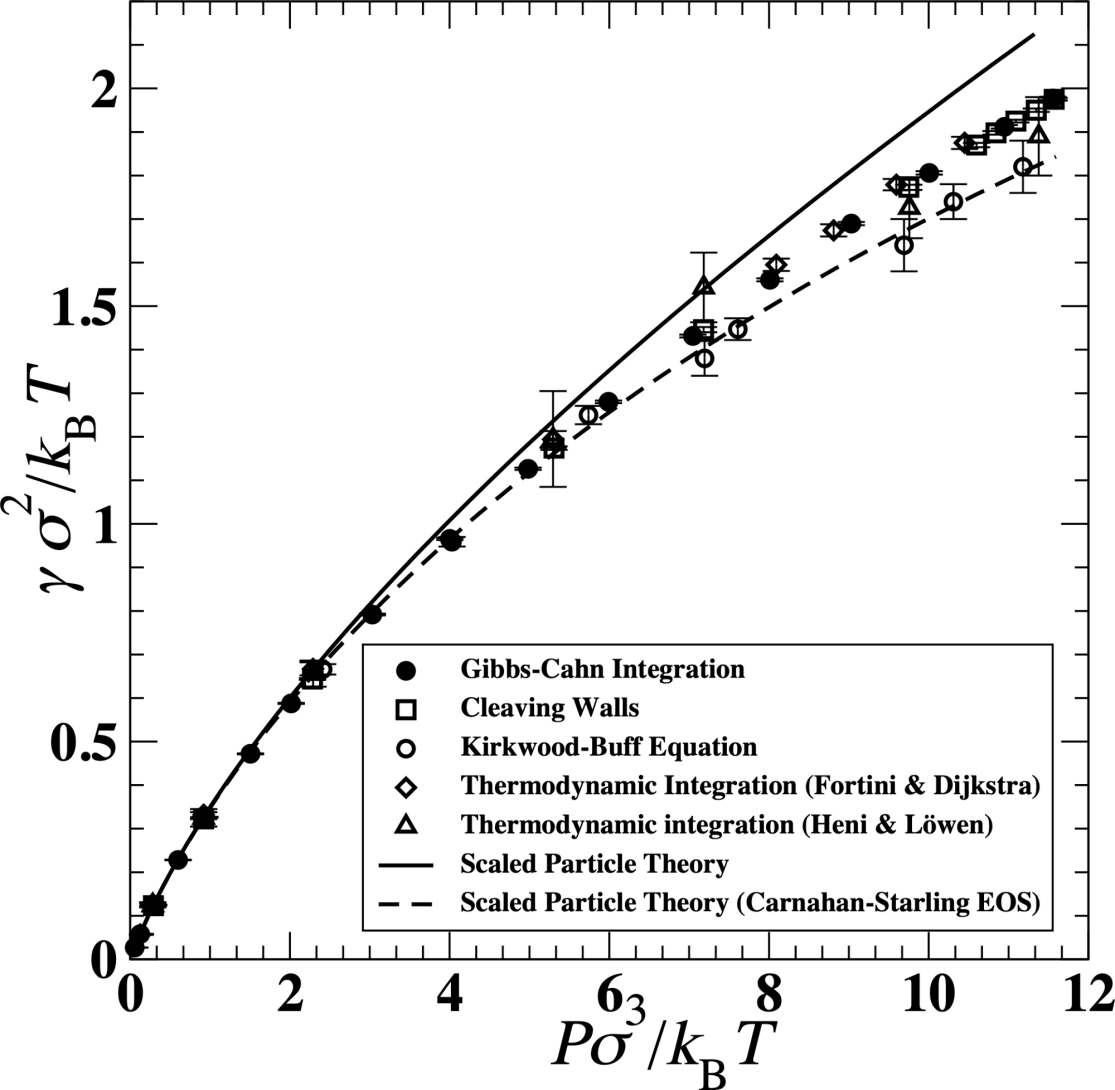
Summary of IFE results for the hard-sphere/structureless hard-wall system calculated using Gibbs–Cahn integration,^318^ cleaving walls,^317^ the Kirkwood–Buff equation, eq 8,^314^ and thermodynamic integration.^315,316^ The solid and dashed lines are theoretical results from standard scaled particle theory^320^ and scaled particle theory using the Carnahan–Starling equation of state (EOS) pressure to correct for the position of the wall, respectively.

The Gibbs–Cahn formalism can also be used to calculate γ for hard-sphere (and hard-disk) fluids at curved hard walls to test theories of the curvature dependence, such as the so-called morphometric thermodynamics,^321^ which states, for a 3D system, that the curvature dependence of γ can be determined as a linear combination of the mean and Gaussian curvatures. Evaluation of the dependence of the IFE for the hard sphere fluid at spherical and cylindrical walls on the wall radius shows that morphometric thermodynamics is valid except at high densities near the freezing transition.^301,302^ Similar conclusions are obtained for the hard-disk fluid on a circular wall.^322,323^ Morphometric thermodynamics was also shown to hold for surfaces of negative curvature (a hard-disk fluid inside a circular container) except at high density and very high curvature (small radius R ).^304^ The results obtained for these systems using classical density functional theory were also shown to be in very good agreement with simulations.

### 6.2 Lennard-Jones Particles

The Lennard-Jones potential has also been extensively used to calculate liquid–solid interfacial free energies, and, in particular, the Broughton–Gilmer version of the Lennard-Jones potential is the first model for which γ_*sl*_ has been estimated using computer simulations in 1986.^324^ (Note that the truncated Lennard-Jones potential given by Broughton and Gilmer in their original paper contains a typo, which was corrected in a later paper by Davidchack and Laird^96^)

The methodology employed was the cleaving method, presented as a direct TI-based approach to compute IFEs with a resolution capable of discriminating between the different values of γ_*sl*_(**n̂**). Both the cleaving walls^96^ and the capillary fluctuations^208^ methods were used with the Broughton–Gilmer potential to obtain results at the triple point consistent with those of ref 324 for the fcc crystal orientations summarized in Table 2. Davidchack and Laird^96^ also performed cleaving walls calculations to obtain γ_*sl*_ for the Broughton–Gilmer Lennard-Jones potential at higher temperatures than those used in ref 324 and used Gibbs–Cahn integration^118^ to estimate γ_*sl*_ along the coexistence line. Their method, which relies on the calculation of the IFE and interfacial stress of the crystal–melt interface (*f*_*ij*_) has also been tested for the Lennard-Jones potential, producing consistent results (see refs 145 and 325). The cleaving walls have also been used by Mu and Song^221^ in combination with free energy perturbation calculations, while Benjamin and Horbach^326^ have used a thin flat Gaussian wall potential in combination with the cleaving walls in order to stabilize the interfacial system. Alternative techniques, based on thermodynamic integration methods such as the phantom wall^247^ and mold integration,^228^ have also been employed to compute the IFEs of fcc crystal phases of the standard Lennard-Jones potential and the Broughton–Gilmer modification, respectively. In addition, values from nucleation studies using the seeding technique and a CNT analysis have also predicted average values of γ_*sl*_ consistent with previous independent direct estimates for the Broughton–Gilmer potential at different pressures.^327^ In Table 2, we provide the reported values of γ_*sl*_ for both the standard Lennard-Jones potential and the Broughton–Gilmer version from different direct and indirect calculations.

**Table 2:** Solid–Melt Interfacial Free Energy (*γ*_*sl*_ in *ϵ*/*σ* ^2^) of the Broughton–Gilmer Lennard-Jones Potential for Different Crystal Orientations of an fcc Crystal in Contact with Its Melt at Coexistence Conditions of the Temperatures Indicated^a^

γ_*sl*_ fcc	technique	*T* ^a^	(100)	(110)	(111)	average γ_*sl*_
Broughton and Gilmer 1986 ^324^	cleaving potential	0.617	0.34(2)	0.36(2)	0.35(1)	
Morris and Song 2003 ^208^	CF	0.617	0.369(8)	0.361(8)	0.355(8)	
Davidchack and Laird 2003 ^96^	cleaving wall	0.617	0.371(3)	0.360(3)	0.347(3)	
Mu and Song 2006 ^221^	Cleaving-FEP	0.617	0.371(3)	0.361(3)	0.354(3)	
Angioletti-Uberti et al. 2010 ^292^	metadynamics	0.617	0.370(2)			
Espinosa et al. 2014 ^228^	MI	0.617	0.372(8)		0.350(8)	
Sanchez-Burgos et al. 2024 ^145^	MI	0.617	0.372(8)		0.347(8)	
Baidakov et al. 2013 ^325^,^a^	cleaving	0.617	0.430(4)	0.422(4)	0.408(4)	
Montero de Hijes et al. 2019 ^327^	CNT	0.617				0.358(3)
Benjamin and Horbach 2014 ^326^	Cleaving-TI	0.617	0.372(5)	0.357(3)	0.344(6)	
Davidchack and Laird 2003 ^96^	cleaving wall	1.0	0.562(6)	0.543(6)	0.508(8)	
Sanchez-Burgos et al. 2024 ^145^	MI	1.0	0.562(8)		0.510(8)	
Montero de Hijes et al. 2019 ^327^	CNT	1.0				0.543(6)
Benjamin and Horbach 2014 ^326^	Cleaving-TI	1.0	0.572(3)	0.545(3)	0.515(6)	
Davidchack and Laird 2003 ^96^	cleaving wall	1.5	0.84(2)	0.82(2)	0.75(3)	
Sanchez-Burgos et al. 2024 ^145^	MI	1.5	0.845(9)		0.815(9)	
Benjamin and Horbach 2014 ^326^	Cleaving-TI	1.5	0.866(5)	0.785(6)	0.774(7)	

a In the last column we report the averaged values of *γ*_*sl*_ obtained from nucleation studies. Various computational approaches have been employed for the determination of *γ*_*sl*_: cleaving potential and cleaving wall, cleaving with thermodynamic integration [Cleaving-TI] and cleaving with free energy perturbation [Cleaving-FEP], capillary wave fluctuations [CF], metadynamics, mold integration [MI], and classical nucleation theory analysis of nucleation free energy barriers and extrapolation to coexistence [CNT]. Numbers in parentheses indicate the estimated error in the last digit(s) shown.

b For a standard Lennard-Jones potential, see ref 325.

All calculations of γ_*sl*_ for the different crystal planes (100), (110), and (111)) at the triple point temperature (T* = 0.617) of the Broughton–Gilmer Lennard-Jones potential agree independently of the technique employed within the uncertainty of the calculation. Only the first value provided by Broughton and Gilmer for the (100) plane slightly underestimates the most recent calculated IFEs by different groups. Furthermore, nucleation studies using the seeding technique^327^ also report an average value of γ_*sl*_ that perfectly matches direct estimates under coexistence conditions of different groups (Table 2). On the other hand, for the standard Lennard-Jones potential (shifted and truncated at 2.5 σ), the values of γ_*sl*_ at the same temperature are approximately 10*%* higher.^325^ This is expected because the potential shape is not equivalent to the Broughton–Gilmer expression. However, the fact that the relative values of the IFE for different crystal orientations match those found for the corresponding planes using the Brougton-Gilmer Lennard-Jones potential gives credibility to these independent calculations. At higher temperatures and pressures, cleaving techniques,^96^ mold integration,^228^ and seeding techniques^327^ have been also used to obtain the value of γ_*sl*_. The agreement at T* = 1 between direct estimations for different orientations is excellent, as well as the extrapolation of values from critical fcc clusters under supercooling conditions.^327^ For T* = 1.5, the reported IFEs for the (100) plane match within the uncertainty, while those for the (111) orientation differ somewhat.^145^ More work is probably required to clarify the origin of this small discrepancy. However, the Lennard-Jones model and in particular the Broughton and Gilmer version have excellent potential to validate novel techniques to estimate γ_*sl*_ alongside the hard-sphere model.

## 7 Interfacial Free Energies of Realistic Systems

In the previous section, we have shown how some indirect and direct simulation techniques have been used in the literature to deal with the standard hard-sphere and Lennard-Jones systems, which are characterized by simple intermolecular interaction potentials. Although these systems are invaluable for developing new methodologies in computer simulation, sooner or later it is necessary to extend the applicability of new techniques to more complex systems. Increasing complexity, in the case of solid–fluid interfaces, can mean more complex intermolecular interactions between the molecules forming the system, but also more complex solid crystalline structures. In this section, we concentrate on the determination of ice–aqueous solution IFEs, with particular emphasis on pure water systems and on a class of aqueous solutions that are able to form clathrate hydrates.^235,236^

### 7.1 Water

Water is probably the simplest (but by no means simple) molecule that can be found in solid, liquid, and gas phases in nature under ordinary temperature and pressure conditions. In addition to its significance in our daily lives, water is also a fascinating subject of study because of its remarkable properties. Both condensed phases, liquid and solid, present a series of anomalies compared to other compounds.^328,329^ The complexity of the liquid–solid water phase diagram (there are at least 13 solid ice structures^330–334^ ) and the existence of a liquid–liquid phase transition are particularly noteworthy.^335,336^ Taking these into account, it is easy to understand why obtaining a deep understanding of the factors that control the homogeneous nucleation of ice in water, including the solid–liquid IFE, is still a formidable challenge.

Several authors have reported their findings obtained from different simulation techniques, thermodynamic conditions, and water models. The most relevant results for the Ih ice–water IFE are summarized in Table 3. The first time γ_*sm*_ was computed entirely from molecular simulations was in 2005 by Haymet et al.^342^ They determined γ_*sm*_ predicted by the SPC/E water model for the basal ice Ih–water interface, obtaining a value of 39(4) mJ m^–2^. However, this result was of limited value, since it was obtained using the mechanical route without considering any corrections. In 2007, Wang et al.^337^ determined γ_*sm*_ for the ice Ih–water interface through MD simulations using the TIP4P-Ew and TIP5P-Ew water models and the indirect superheating (or undercooling) hysteresis method.^343^ This technique relates the solid–melt interfacial free energy to the melting temperature, the enthalpy change of melting per unit volume, and a dimensionless nucleation barrier parameter. Based on CNT, this parameter is obtained from the maximum superheating (or undercooling) temperature at which the solid/melt system can be heated (or cooled) beyond the melting temperature. In this work, the values of γ_*sm*_ obtained for both TIP4P-Ew and TIP5P-Ew models at 1 bar and the corresponding melting temperature were 37 and 42 mJ m^–2^, respectively. Although the values obtained in these two papers agree with each other, they are higher than literature experimental data. As Wang et al.^337^ claimed in their work, accurate superheating literature data for ice are scarce because heterogeneous melting makes it difficult to measure the correct superheating limit. On the other hand, homogeneous crystallization of liquid water is rarely reported in molecular dynamics simulations because ice nucleation is a rare event in a homogeneous bulk of undercooled water. The same authors claimed that more accurate values of γ_*sm*_ can be computed by using more rigorous techniques such as the cleaving wall or capillary fluctuations methods.

**Table 3:** Solid–Melt Interfacial Free Energy for the Ih-Water System as Obtained from Different Water Models, Thermodynamic Conditions, and Methods^a^

γ_*sm*_ Ih	technique	water model	*T* (K)	*P* (bar)	basal	prism I	prism II	average γ_*sm*_
Wang et al. 2007 ^337^	CNT	TIP4P-Ew	244	1				37(3)
		TIP5P-Ew	254	1				43(3)
Handel and Davidchack 2008 ^223^	Cleaving	TIP4P	219	1	23.3(8)	23.6(1.0)	24.7(8)	
Li et al. 2011 ^338^ ^a^	CNT	mW	274.6	1				31.01(21)
Reinhardt and Doye 2011 ^180^ ^a^	CNT	mW	220	1				23
Davidchack et al. 2012 ^224^	Cleaving	TIP4P	230	1	24.5(6)	27.6(7)	27.5(7)	
		TIP4P-Ew	245	1	25.5(7)	28.9(8)	28.3(7)	
		TIP5P-Ew	270	1	27.8(9)	27.4(8)	31.6(7)	
Reinhardt and Doye 2013 ^339^ ^a^	CNT	TIP4*P*/2005	240	1				24
			252	1				26.1
Sanz et al. 2013 ^185^	CNT	TIP4*P*/2005	252	1				28.7
		TIP4P/Ice	270	1				28.7
Benet et al. 2014 ^195^	CF	TIP4*P*/2005	248.5	1	27(2)	28(2)	28(2)	
Espinosa et al. 2014 ^340^	CNT	TIP4P/Ice	272	1				30.8
		TIP4*P*/2005	252	1				29.0
		TIP4P	230	1				25.6
Espinosa et al. 2016 ^186^		mW	274.6	1				35
Espinosa et al. 2016 ^229^	MI	TIP4P/Ice	272	1	27.2(8)	31.6(8)	30.7(8)	29.8(8)
		TIP4*P*/2005	252	1	27.2(8)	29.5(8)	30.0(8)	28.9(8)
		TIP4P	230	1	25.5(8)	28.2(8)	28.0(8)	27.2(8)
		mW	274.6	1	34.5(8)	35.1(8)	35.2(8)	34.9(8)
Espinosa et al. 2016 ^7^	MI and CNT	TIP4P/Ice	246	2000				40
		mW	270.7	2000				38.4
		mW	261.6	5000				40.3
Ambler et al. 2017 ^341^ ^b^	CF	mW	274.6	1	33.7(4)	36.0(3)	36.1(3)	
Montero et al. 2023 ^231^	MI	TIP4P/Ice	279.0	–2600	27.1(1.5)			
		TIP4P/Ice	280.0	–2000	26.5(1.5)			
		TIP4P/Ice	278.0	–1000	25.6(1.5)			
		TIP4P/Ice	260.0	1000	29.0(1.5)			
		TIP4P/Ice	246.5	2000	37.2(1.5)			

a Basal, prism I, and prism II are the solid–melt interfacial free energy when the aqueous phase is in contact with the Ih basal, prism I, and prism II faces, respectively. The last column represents *γ*_*sm*_ averaged over all the faces. In all cases, the solid–melt interfacial free energy values are expressed in mJ m^–2^. Various computational approaches have been employed to determine *γ*_*sm*_: superheating (or undercooling) hysteresis [SUH], cleaving, capillary wave fluctuations [CF], mold integration [MI], and classical nucleation theory analysis of nucleation free energy barriers and extrapolation to coexistence [CNT]. Numbers in parentheses indicate the estimated error on the last digit(s) shown.

b An initial 50/50% Ih/Ic cryo embryo is used as the initial cluster seed.

c The results presented in the table are the average obtained over two different order parameters employed by the original authors.

In 2008, Handel et al.^223^ determined γ_*sm*_ for the first time using a direct simulation technique, the cleaving method. In this pioneering work, Handel et al. determined γ_*sm*_ for the three principal crystal ice Ih planes, namely basal, primary prismatic (pI), and secondary prismatic (pII), using molecular dynamics simulations and the TIP4P water model. The values obtained in this work for the same three principal planes of ice Ih in contact by a planar interface with pure water at ambient pressure and coexistence temperature were 23.8(8), 23.6(10), and 24.7(8) mJ m^–2^, respectively. Later, the same authors extended their original work^224^ to two other water models: TIP4P-Ew and TIP5P-Ew. In addition, they revisited their results obtained with the TIP4P water model, going beyond the truncated electrostatic interactions used in the original work by using Ewald sums to account for the full electrostatic interaction. The new values obtained using the cleaving wall method and the TIP4P model were 24.5(6), 27.6(7), and 27.5(7) mJ m^–2^ for the basal, pI and pII Ih ice planes, respectively. They obtained similar results by using the TIP4P-Ew model (25.5(7), 28.9(8), and 28.3(7) mJ m^–2^) and the TIP5P-Ew model (27.8(9), 27.4(8), and 31.6(7) mJ m^–2^) for the three principal planes of ice Ih at 1 bar and the coexistence temperature. In all cases, the agreement between simulation and experimental data was very good.

Benet et al.^195^ determined γ_*sm*_ using the capillary fluctuations method for the basal, pI, and pII ice Ih–water interfaces, obtaining values 27(2), 28(2), and 28(2) mJ m^–2^, respectively, using the TIP4*P*/2005 water model at 1 bar and the coexistence temperature. Some years later, Ambler et al.^341^ applied the same technique to determine the average Ih, Ic, and 0 ice–water γ_*sm*_ values using the coarse-grained monatomic water (mW) model. In all cases, they obtained a γ_*sm*_ value around 35 mJ m^–2^ at 1 bar and the melting temperature. It is interesting to note that the results reported by Ambler et al.^341^ are ≈20*%* higher than those obtained by Benet et al.^195^ with the same technique. However, this can be explained by noting that γ_*sm*_ is extremely sensitive to the water models used in both studies, as well as to their respective ice–water coexistence temperatures.

As explained in section 4.2, γ_*sm*_ can be related to the free energy barrier, Δ*G*_crit_, required for the formation of a critical solid nucleus in the middle of a homogeneous liquid. Although this is an indirect method for determining γ_*sm*_ through the calculation of Δ*G*_crit_ and has some shortcomings (see sections 4.2, 8, and 9 for more details), this approach has great versatility because Δ*G*_crit_ can be determined from different simulation techniques such as forward flux sampling, umbrella sampling, and seeding. In 2011, Li et al.^338^ studied homogeneous ice nucleation at 1 bar from supercooled water using forward flux sampling, MD simulations, and the mW water model. The nucleating ice embryo contains ice Ic and Ih structures in a 50%/50% mixture. Combining their findings with CNT, they estimated a γ_*sm*_ value of 31.01(21) mJ m^–2^. A year later, Reinhardt and Doye^180^ studied the homogeneous nucleation of ice from supercooled liquid water with Monte Carlo simulations using umbrella sampling and the mW water model. By combining their findings with CNT, they obtained a value of γ_*sm*_ at 1 bar and under supercooling conditions (23.0 mJ m^–2^ at 220 K). Later, the same authors^339^ extended their results to the TIP4P/2005 water model, obtaining IFE values of 24.0 and 26.1 mJ m^–2^ at 240 (supercooled conditions) and 252 K (melting temperature), respectively. Interestingly, the two models yielded similar values of γ_*sm*_ at 220–240 K even though the water model and the degree of supercooling were different. However, their results seem to be lower than those reported by Li et al.^338^ As Reinhardt and Doye claimed in their work,^180^ these discrepancies arise because the two groups used different order parameters to monitor the number of water molecules in the solid critical cluster.

In 2013, Sanz et al.^185^ combined for the first time the seeding method and CNT to determine the ice Ih–water γ_*sm*_ at 1 bar and the melting temperature using the TIP4*P*/2005 and TIP4P/Ice water models. They obtained, for both models, a value of 28.9 mJ m^–2^, in very good agreement with the results obtained by Reinhardt and Doye for the TIP4P/2005 water model.^339^ A year later, some of the authors of the original work of Sanz et al.^185^ employed the same methodology and determined the ice Ih–water γ_*sm*_ predicted by the TIP4P, TIP4P/Ice, TIP4P/2005, and mW water models at 1 bar and in a broad range of supercooled temperature conditions.^340^ By extrapolating γ_*sm*_ to the melting temperature for each model, they reported values of 25.6, 30.8 and 29.0 mJ m^−2^ for the TIP4P,TIP4P/Ice and TIP4P/2005 water models, respectively.

For mW the correct value extrapolated at the melting temperature was 35.0 mJ m^−2^ as was later reported^186,327^ (due to an insufficient equilibration time, the value of γ_*sm*_ reported in ref 340 for mW was incorrect). The same authors extended this study to determine the ice Ic–water γ_*sm*_ at 1 bar and the corresponding melting temperature predicted by the TIP4P/Ice model.^344^ They obtained a value of 31(3) mJ m^–2^, in very good agreement with their previous results. The similar results obtained for both Ih and Ic ice–water interfaces are consistent with the fact that both ice structures have the same nucleation rate, which means that under these conditions the formation of the two ice I polymorphs is equally favored. Very recently, Tipeev and Zanotto^345^ employed the same methodology to determine the Ic–water γ_*sm*_ at 1 bar and supercooled conditions (215–240 K) using the mW water model. They obtained an average value of 27.5(11) mJ m^–2^ for the crystal nuclei seeded in the supercooled water.

Although the seeding + CNT combination provides very reliable results, it is still an indirect way to evaluate solid–fluid IFEs. On the other hand, the mold integration methodology (see section 5.2) proposed by Espinosa et al.^228^ provides a direct and relatively simple way to predict solid–fluid IFEs from a fundamental point of view. The same authors of the original work where the mold integration method was proposed determined the ice Ih–water γ_*sm*_ value using the TIP4P/Ice, TIP4P/2005, TIP4P, and mW water models with the mold integration method. They calculated the ice Ih–water γ_*sm*_ at 1 bar and the melting temperature of each water model, for the three main planes of the ice Ih (basal, prism I, and II) obtaining an average value of 29.8(8), 28.9(8), 27.2(8), and 34.9(8) mJ m^–2^ for the TIP4P/Ice, TIP4*P*/2005, TIP4P, and mW water models, respectively.^229^ They also calculated the ice Ic–water IFE for three different ice Ic planes in contact with the water phase ((100), (110), and (111)), obtaining an average value of 30.1(8) mJ m^–2^. As the authors claimed in previous work,^340^ there are no significant differences in the ice–water IFE between the ice I polymorphs. These results are in very good agreement with those reported previously in the literature using seeding + CNT,^185,340,344^ umbrella sampling + CNT,^339^ capillary fluctuations,^195^ and the cleaving walls method.^223,224^ Shortly after determining the value of ice Ih–water γ_*sm*_ at 1 bar and melting temperature, some of the authors of the original work extended that previous study and determined γ_*sm*_ at 2000 bar to analyze the effect of pressure on the interfacial free energy using the mold integration methodology and the TIP4P/Ice water model.^7^ They obtained an increase of γ_*sm*_ with pressure of ∼10 mJ m^–2^. In the same work, they determined the ice Ih basal plane–water γ_*sm*_ at 2000 and 5000 bar using the mW water model and the mold integration methodology. As for the case of the TIP4P/Ice water model, they observed an increase of γ_*sm*_ when the pressure was increased. In the same work,^7^ the authors determined the ice 0–water γ_*sm*_ at 1 bar using the mW water model and the mold integration technique, obtaining a value of 35.4 mJ m^–2^. All of the results obtained in this work were also obtained by the seeding + CNT combination, obtaining excellent agreement with those obtained by the mold integration methodology. Finally, it is worth mentioning that recently^231^ the mold integration methodology has been employed to determine the basal ice Ih–water γ_*sm*_ at coexistence temperatures from −2600 to 2000 bar using the TIP4P/Ice water model. This study was carried out by some of the authors of the original work of Espinosa et al.^228^ and they reported a γ_*sm*_ minimum of 26(1) mJ m^–2^ around −2000 bar.

Sanchez-Burgos et al.^145^ determined γ_*sm*_ along the ice Ih–water coexistence line from single-state calculations utilizing the Gibbs–Cahn integration method.^88^ They used the mW water model and the result previously obtained using the mold integration methodology by some of them^7^ as the initial single-state IFE value. They find excellent agreement between the results obtained following the mold integration methodology^7^ and those obtained from the Gibbs–Cahn integration approach, proving the power of this approach to quantify the dependence of the IFE along a coexistence line.

### 7.2 Hydrates

Clathrates are nonstoichiometric inclusion compounds where guest molecules, such as methane (CH_4_), carbon dioxide (CO_2_), hydrogen (H_2_), nitrogen (N_2_), and tetrahydrofuran (THF), are trapped within cavities formed by a periodic network of associating molecules or host.^235,236^ These associating molecules interact through not only van der Waals forces but also specific, short-range, and highly directional interactions that cause the network arrangement of the system. When the associating system is formed by water molecules, the association is mediated through hydrogen bonding, and clathrates are also known as hydrates. Hydrates crystallize into several distinct structures^235,236^ and also exhibit proton disorder, satisfying the Bernal–Foller rules,^346^ as do various phases of ice, including Ih ice.

However, hydrates are much more complex than ice. The nature and concentration of guest molecules in a hydrate greatly affect the stability conditions of these compounds as well as the crystalline structure adopted by the hydrate. As we have already seen in section 5.2, small molecules, such as CO_2_ or CH_4_, crystallize in the sI structure.^235,236^ However, hydrates of medium-size molecules, such as isobutane, propane, cyclopentane, and THF, crystallize in the sII structure, which also shows cubic symmetry. The sII unit cell is more complex than the sI structure, being made up of 136 water molecules distributed in 16 D cages (pentagonal dodecahedron or 5^12^) and 8 H cages (hexakaidecahedron or 5^12^6^4^).^235,236^ The D or “small cages” are the same in both structures, but the “large cages” (H) are larger in the sII structure, allowing them to accommodate larger molecules. The sII structure has the peculiarity that it can be stabilized by medium-sized or small molecules, such as H_2_ or N_2_ through multiple occupancy of the H cages.^347–349^

According to the literature, CO_2_ and CH_4_ hydrates exhibit mainly a single occupancy in each cage but in such a way that each unit cell can accommodate eight CO_2_ or CH_4_ molecules.^235,350–354^ However, THF occupies only the H cages (5^12^6^4^) of the sII hydrate structure.^355,356^ The T cages (5^12^) remain empty and can be occupied by other small guest molecules of low molecular weight. The formation of sII structures of hydrates with small molecules such as N_2_ and H_2_ is unusual. However, the nonstoichiometric nature of hydrates offers the possibility of multiple cage occupancy. The explanation of the preference to form sII hydrates instead of sI hydrates is that the N_2_ and H_2_ molecules better stabilize small hydrate cages, which are more common in the sII crystallographic structure.^357–361^ Multiple occupancy of these molecular cages is another complexity that makes hydrates fascinating and very complex substances to model and understand from a molecular perspective. Obviously, the prediction of hydrate-water IFEs is not an exception.

The first calculation of the CH_4_ hydrate–water IFE was performed by Jacobson and Molinero and dates back to 2011.^362^ Water molecules were modeled using the mW water model.^363^ The guest molecule, which the authors call M,^364,365^ is represented by a single particle with properties intermediate between CH_4_ and CO_2_. The authors performed seeding simulations at 50 MPa using a slab of M liquid in contact with a saturated water solution with M containing clusters of M hydrates of different sizes to determine the melting temperatures of the crystalline nuclei. Combining these results with the well-known Gibbs–Thomson relationship,^366–368^ it was possible to estimate the M hydrate–water IFE, γ_*sx*_, obtaining a value of 36(2) mJ m^–2^. This value agrees well with the experimental data obtained by Uchida et al.^369,370^ and Anderson et al.^371,372^ for the real CH_4_ hydrate–water interface, γ_*sx*_ = 34(6) and 32(3) mJ m^–2^, respectively. It also agrees well with the experimental values of the free energy values of the CO_2_ hydrate-water interface, obtained independently by the same authors, γ_*sx*_ = 28(6) and 30(3) mJ m^–2^. One year later, Knott et al.^373^ used the mW model for water and a single-site Lennard-Jones potential for methane to predict the IFE of the CH_4_ hydrate using seeding simulations in combination with CNT^164–166^ (see section 4.2 for more details). They obtained a value for the IFE, γ_*sx*_ = 31 mJ m^–2^, that was also in good agreement with experimental data taken from the literature.

More recently, Grabowska et al.^374,375^ have estimated homogeneous nucleation rates for the CH_4_ hydrate from seeding simulations at 400 bar for a supercooling of 35 K (260 K) using the TIP4P/ice model^376^ and a Lennard-Jones center to model methane.^377,378^ Using simulations and CNT, they compared γ_*sx*_ values for two critical clusters found at 400 bar and 260 K as a function of their radius and extrapolated to the planar limit (see Figure 14 in the work of Grabowska et al.^375^). Their calculations suggest a value of around 38 mJ m^–2^ for the CH_4_ hydrate–water planar interface. The coexistence temperature of this hydrate at 400 bar is approximately 295 K. IFE values under supercooling conditions *increase* as the temperature increases (at constant pressure).^379^ Thus, the results of this work seem to suggest a higher value of γ_*sx*_ for the planar CH_4_ hydrate–water interface than for the CO_2_ hydrate-water IFE of a planar interface. The value found by Grabowska et al.^375^ for the CH_4_ hydrate–water planar interface from simulation is higher than the experimental value found by Anderson et al.,^371,372^ which is equal to 32 mJ m^–2^. However, the value found from seeding simulations seems to be consistent with the preliminary results obtained by Zerón et al.^380^ using the two extensions of the mold integration technique to estimate the hydrate–water IFEs. These authors have obtained values of 43(2)–44(1) mJ m^–2^ for CH_4_ hydrate–water IFE using the same molecular models for water and CH_4_.

All of the works just presented use indirect methods to determine the CH_4_ and CO_2_ hydrate–water IFE, including the combination of seeding simulations with CNT or the use of the Gibbs–Thomson relationship. However, as discussed in section 5.2, Algaba and collaborators have obtained the interfacial free energy of CO_2_ hydrate-water using the mold integration host and guest methodologies.^232–234^ In both cases, water molecules are modeled using the well-known TIP4P/Ice^376^ and TraPPE-UA force field for CO_2_ molecules.^381^ In the first case, the authors obtained a value of γ_*sx*_ = 29(2) mJ m^–2^, and in the second case they obtained a value of γ_*sx*_ = 30(2) mJ m^–2^. Both values are in excellent agreement with the experimental data of Uchida et al.,^369,370^ 28(6) mJ m^–2^, and Anderson et al.,^371,372^ 30(3) mJ m^–2^, discussed above.

Whereas CH_4_ and CO_2_ hydrates crystallize in the sI structure, many other aqueous solutions form hydrates in the more complex sII structure. Some authors have used the mold integration technique to estimate IFEs of two different hydrates. Torrejón et al.^382^ predicted the THF hydrate–water IFE using the mold integration (host) technique at 500 bar under the conditions defined by the univariant two-phase coexistence line of the hydrate. This hydrate exhibits a sII crystallographic structure more complex than the sI structure of the CH_4_ and CO_2_ hydrates. The IFE obtained, 27(2) mJ m^–2^, is in excellent agreement with the experimental data taken from the literature, 24(8) mJ m^–2^.^383,384^

## 8 Role of Interfacial Free Energy in Crystal Nucleation

In section 4.2 we discussed how CNT can be used to calculate the IFE. In this section, we examine how the IFE can be used to explore nucleation and its challenges in the light of the previous sections. However, it is not our intention to add another full-scale review on the subject of nucleation; we refer readers who want to be introduced to the vast literature on nucleation to the relevant chapters in ref 385 and then to reviews such as refs 386–392 .

Classical nucleation theory, as developed by Volmer–Weber and Becke−Döring^165,167,393^ aims to describe homogeneous nucleation, although it has been extended to the heterogeneous case (we refer the interested reader to the classical works of Turnbull,^394^ Fletcher,^395^ and refs 173, 396, and 397 for modern accounts). Nucleation is defined in terms of the nucleation rate *J*_CNT_, which represents the number of critical nuclei, *N*_c_, that appear per unit time and volume. We add the subscript “CNT” to emphasize the fact that we are working within the framework of classical nucleation theory. The nucleation rate is defined in terms of a product of a kinetic factor, *J*_kin_, describing the rate of attachment of particles to the growing cluster, and a thermodynamic factor, *J*_thd_, related to the free energy barrier of nucleation (It is worth noting that, to date, the thermodynamic contribution has received much more attention in the literature). In general, the nucleation rate can be written as follows:

$$J_{\mathrm{CNT}}=J_{\mathrm{kin}}J_{\mathrm{thd}}=J_{\mathrm{kin}}\exp\left(-\frac{\Delta G_{\mathrm{crit}}}{k_{B}T}\right)$$ 55

where Δ*G*_crit_ is the thermodynamic barrier to nucleation. This is strongly dependent on the IFE as can be seen from eq 13, where Δ*G*_crit_ ∝ γ_*sl*_^3^. The nature of this term will depend on the rate-determining step of the nucleation mechanism.

In studying systems (such as metals) with solid–melt and solid–semisolid interfaces, modeling strategies using CNT are now employed in phase prediction and precipitation studies. These can be collectively termed classical nucleation growth theories. These models have been built largely by exploring the kinetic prefactor in CNT.^91,398^ The kinetic prefactor in this case is given by

$$J_{\mathrm{kin}}=\rho_{l}Z\phi^{+}$$ 56

where ϕ^+^ is the rate of attachment of formula units to the growing cluster, *Z* is the so-called Zeldovich factor, and ρ_*l*_ is the number density of formula units in the fluid phase. In crystallization from solution, the latter term represents the number density of solute molecules in solution, whereas in freezing from a melt it represents the number density of molecules in the melt. The rate of attachment is a feature of atoms hopping from the matrix phase into the nucleus and also of the flow of matter into the matrix surrounding the nucleus. There have been many different interpretations of this term, which is often estimated using forms of jump frequency and diffusion coefficients.^399–401^ The Zeldovich factor can be expressed as^91,398^

$$Z=\frac{1}{2\pi {R_{C}}^{2}\rho_{s}}\sqrt{\frac{\gamma_{sl}}{k_{B}T}}$$ 57

where ρ_*s*_ is the number density of the solid and $R_{C}$ is the critical nucleus radius.^402,403^ We will discuss these in more detail in the next section, where the problem of curved interfaces is considered (see section 9 and eq 71). As can be seen from eq 57, the evaluation of the Zeldovich factor requires knowledge of the IFE that itself is difficult to resolve in the case of a nonplanar interface, which is often present in a growing nucleus.

CNT has been extended in several directions to include different effects. One of these extensions is related to the inclusion of an incubation time, τ, to account for the time required for the clusters to reach a steady state with their environment. In fact, in some applications, such as the solution deposition of organic thin films,^404^ the process is far from equilibrium. Therefore, the transient concentration of critical nuclei in such systems differs from its steady-state value. One model that accounts for transient nucleation was derived by Kampmann and Wagner^405^ and describes the variation of the number of nuclei over time, *N*_c_, as^402,403^

$$\frac{dN_{c}}{dt}=\rho_{l}Z\phi^{+}\exp\left(-\frac{\Delta G_{\mathrm{crit}}}{k_{B}T}\right)\exp\left(-\frac{\tau }{t}\right)$$ 58

Several numerical methods have been built around this model, with the Kampmann–Wagner numerical model becoming one of the most popular due to its few basic assumptions and its ability to work with grain coarsening.^402,406^ This model has also been extended and implemented in various ways to explore precipitation dynamics.^402,407^ The expression for the incubation time τ depends on the IFE (see ref 402), further increasing the dependence of the theory on this parameter.

It should be clear by now the important role γ_*sl*_ plays in the theory of nucleation, and it should be expected that different approximations have been proposed for it. Turnbull^106,408^ proposed a relationship equating the IFE to the latent heat of fusion Δ*H*_*f*_ for crystal–melt systems:

$$\gamma_{sl}=B{\rho_{s}}^{2/3}\left(\Delta H_{f}/N_{A}\right)$$ 59

where B is an empirical coefficient (about 0.45 for most metals; about 0.32 for most nonmetals,^409^ although the precise value is system-dependent, see, for instance, the value reported for hcp metals^410^), and *N*_A_ is the Avogadro number. The empirical expression shown in eq 59 can give reasonable results when dealing with systems that include one or two dominant species where the values of solid–melt IFE are generally thought to be of the order of ∼10 mJ m^–2^, and little distinction is made between enthalpy and free energy. Moving to multicomponent systems such as high-entropy alloys where there is often much more heterogeneity at the interface, using eq 59 may become more problematic. Moving beyond metals to ionic melts, greater directionality appears around atom positioning, and therefore a much greater degree of difference between the IFE values can be found,^161,411^ which further increases doubt concerning the accuracy of such empirical correlations. Two further caveats about the use of eq 59 for nucleation studies should be made. The first is that the rule attempts to estimate γ_*sl*_ for a planar solid–fluid interface, but for nucleation studies one needs the value of γ_*sl*_ for a curved interface (i.e., for the critical nucleus) that often is significantly different (this issue will be discussed later in this review). Second, even for a planar interface it is not clear which value of B should be used. Using the exact values of γ_*sl*_ obtained rigorously for the ice Ih–water interface, it has been found^229^ that at 1 bar the value of B is 0.34 for the TIP4P/Ice model of water and 0.39 for the mW model of water. Thus, there is no universal value of B even for an ice Ih–water interface. In fact, the situation is even worse since for the Ih–water interface of the TIP4P/Ice model at 2000 bar one obtains a value of B of 0.58.^231^ For these reasons, one should be extremely careful when using Turnbull’s rule to estimate γ_*sl*_ for nucleation studies.

More challenges emerge when we consider nucleation from a solution. The use of atomistic simulations to study nucleation from solution was recently reviewed by Finney and Salvalaglio,^412^ building on the reviews of Agarwal and Peters^413^ and Sosso et al.^4^ We therefore limit our discussion to issues concerning the IFE. There are several compilations of IFEs extracted from crystallization data; the most extensive is probably that of Söhnel (1982).^414^ As with Turnbull’s analysis of metal solid–melt interfaces (i.e., eq 59 reported above), other empirical correlations have been proposed to estimate the IFEs for solutes–solvent systems. These fit reasonably well to expressions of the form

$$\gamma_{sl}=\kappa_{1}\log_{10}C_{\mathrm{eq}}+\kappa_{2}$$ 60

where *C*_eq_ is the solubility of the solute in water and κ_1_ and κ_2_ are fitted parameters.^414^

For crystallization from solution, the kinetic prefactor (the term *J*_kin_ in eqs 55 and 56) is again a measure of the attachment frequency of the formula units, but now the rate-determining step in the mechanism may be the desolvation of the ions in solution. This latter effect can be large and dominate the overall activation energy for nucleation; an example is given by the nucleation of LiF in water.^415^ Zimmermann et al.^416^ have argued that the kinetic term in the nucleation of NaCl is also dominated by a dehydration mechanism, although in this case it is still possible to estimate the IFE from the thermodynamic term. Typical values of the kinetic prefactor, *J*_kin_ are of the order of 10^37 ± 3^ s^–1^m^–3^.^189,391^ Simulations have proved particularly useful in assessing the accuracy of CNT, as one can compare values of *J*_CNT_ obtained from direct simulation with those obtained by calculating the CNT parameters using the same potential model. The case of the nucleation rate for the precipitation of NaCl from a supersaturated aqueous solution has proved extremely useful in this regard.^189^ When the Joung–Cheatham potential^417^ is used for NaCl and SPC/E for water,^418^ the values of *J*_CNT_ obtained from CNT are in quite good agreement with the exact values obtained from forward flux sampling^419,420^ (i.e., with deviations of only about 3–4 orders of magnitude). Considering that literature values of *J*_CNT_ obtained from CNT can deviate from experimental values by 20–30 orders of magnitude^421^ or more,^422^ this is not bad. This illustrates that CNT works quite well if the correct value of γ is used.^187,189,416^ However, when compared to experiments, the nucleation rates obtained for the same force field are about 10 orders of magnitude lower than those observed.^189,419^ The fact that the comparison between calculated quantities (with the same force-field but different approaches) gives consistent results but the agreement deteriorates when compared with experiments illustrates there could be deficiencies in the force field rather than in the nucleation theory itself. In fact, using a polarizable force field for *J*_CNT_ improves the predictions significantly.^420^

In Table 4, we report the values (both experimental and calculated) for the IFE of the solid–liquid interface between crystalline NaCl and molten NaCl (top) and brine (bottom). For the solid–liquid interface of molten NaCl, the values of γ_*sm*_ are located around 90 mJ m^–2^ (except for the value reported by Zykova-Timan et al.,^152^ which is much lower). For the solid–liquid interface in aqueous solutions, γ_*sx*_, there is some scatter. However, the greater number of different sources for the value of γ_*sx*_ can be helpful for suggesting some reasons for such a large scatter in the results. We can identify two causes for the dispersion of the data:

1. The values of IFEs for a planar interface are not necessarily identical to those for spherical clusters due to the presence of curvature effects in γ. In this case, we should compare the values of γ_*sx*_ determined for similar systems.
2. Changing the force-field can have an impact on the final value of γ_*sx*_.

**Table 4:** Solid–Melt (*γ*_*sm*_) and Solid-Solution (*γ*_*sx*_) Interfacial Free Energies in mJ m^–2^ for NaCl^g^

	γ_*sm*_	face	technique
Buckle and Ubbelohde 1960 ^431^	84	clusters	expt: homogeneous nucleation
Zykova-Timan et al. 2005 ^152^	37	(100)	calc: contact angle^a^
Espinosa et al. 2015 ^161^	100(10), 114(10)	(100), (111)	calc: mold integration^a^
Benet et al. 2015 ^162^	89(6), 88(6)	(100), (114)	calc: capillary fluctuations^a^

	γ_*sx*_	face	technique
Yeandel et al. 2022 ^239^	128	(100)	calc: Einstein crystal^b^ ^d^
Sanchez-Burgos et al. 2023 ^411^	104(18), 153(11)	(100), (111)	calc: MI^b^ ^c^
Sanchez-Burgos et al. 2023 ^411^	137	planar average	calc: MI^b^ ^c^
Lamas et al. 2021 ^189,411^	150	planar average	calc: fit to seeding simulations^b^ ^c^
Jiang et al. 2018 ^425^	68(7)	clusters	calc: forward flux^e^
Jiang et al. 2018 ^425^	97(5)	clusters	calc: forward flux^b^ ^c^
Jiang et al. 2018 ^425,432^	144(4)	clusters	calc: forward flux^f^
Bulutoghu et al. 2022 ^428^	60–98	clusters	calc: nucleation Two-step model^b^ ^c^
Söhnel 1982 ^414^	38	clusters	expt: nucleation
Na et al. 1994 ^424^	87	clusters	expt: nucleation
Cedeno et al. 2023 ^423^	47.5–61.9	clusters	expt: nucleation

a Numbers in parentheses indicate the estimated error on the last digit(s) shown.

b NaCl BMHFT model force-field used in the calculations.^433,434^

c NaCl Joung-Cheatham model force-field used in the calculations.^417^

d water: SPC/E force-field used in the calculations.^418^

e water: SPC/Fw force-field used in the calculations.^435^

f MAH/BK3 polarizable model force-field used in the calculations.^436^

g AH-TIP4*P*/2005 force-field used in the calculations.^437^

For the planar NaCl-aqueous solution, the average value of the IFE is consistent between the results reported in Sanchez-Burgos et al.^411^ and the one reported in Lamas et al.^189^ The consistency just highlighted is further reinforced when considering that the surface energy values for different crystal faces show a range between 104 and 153 mJ m^–2^ and that all of these values are obtained with the Joung–Cheatham model for NaCl^417^ and a water force-field belonging to the SPC family.^418^ Unfortunately, the fact that the values of γ_*sx*_ are consistent across different calculation techniques does not give us certainty on the correct value when there is no experiment to compare with. For clusters (nucleation), there are some experimental values that can guide us in analyzing the results that come from the calculation. Among the experimental values of γ_*sx*_ reported in Table 4, the value from Söhnel^414^ is the lowest and can most likely be considered outdated, as it was obtained using very old experimental data. Cedeno et al.^423^ used a value of 10^22^ s^–1^m^–3^ for the kinetic prefactor *J*_kin_. Using the recommended value *J*_kin_ of 10^37^ s^–1^m^–3^ instead, their values of γ_*sx*_ are in the range 65–85 mJ m^–2^, consistent with the result reported in Na et al.^424^ If we accept this value for γ_*sx*_ and now compare the results of the calculations, we can see from the results reported in Jiang et al.^425^ that the simulations using a polarizable model outperform the nonpolarizable ones (By polarizable, we mean an explicit polarization model, e.g., using a Drude oscillator-like treatment^426^ rather than effective scaled, but static, charges^427^). An important observation made by Jiang et al. is that, despite the salt solubility being well captured by the AH-TIP4P/2005 model and underestimated by the Joung–Cheatam–SPC/E one, the agreement of the calculations with experimental values is better for the latter model than the former (in the original work the authors refer to nucleation rate, but from Table 4 we can see that it is true for γ_*sx*_ also). These comparisons between polarizable and nonpolarizable models made Jiang et al. advocate for the use (and development) of polarizable force-fields in the calculation of nucleation rates, a statement with which the authors of this review are inclined to agree.

A different story emerges from the result of ref 428. Despite using the Joung–Cheatham model for ions and SPC/E for water, the authors report a range of values for the IFE that is consistent with experiments, i.e., an agreement similar to the polarizable models discussed in previous paragraphs. The solution to this apparent contradiction lies in the fact that the nucleation mechanism simulated in ref 428 belongs to the class of the so-called nonclassical nucleation theories^429,430^ instead of the classical ones considered here. The determination of the mechanisms of nucleation beyond CNT is a separate problem from the one discussed in this work, namely, the calculation of the IFE in molecular simulations, and we will not discuss it further. As we briefly showed here, the seemingly scattered results for the IFE, when put into perspective (i.e., as done by comparing results obtained in the same way, for instance by considering IFE of flat interfaces and clusters separately), are consistent with each other. This last observation, in turn, implies that the methodologies developed and described here are robust.

The CNT theory discussed in this section includes several simplifying approximations that lead to short-comings of the theory. These have frequently been discussed in the literature (see, e.g., refs 389 and 438), and we also present in section 9 a development of the formulation of the CNT theory in which some of these approximations are removed. Two of the most important approximations usually considered in CNT theory (or at least the most related to the current review) are

1. The clusters grow by one unit (often a molecule or a formula unit) at a time to form a spherical cluster with a sharp interface and a crystal structure that is identical to that of the bulk.^173^ The assumption of a single scalar value for γ is reasonable if the nucleus is amorphous (as it will be for a small cluster). However, if the nucleus is faceted the correction is simple. In that case, the IFE is a weighted average over all the facets, given by
$$A_{\mathrm{total}}\left\langle \gamma \right\rangle =\underset{\left\{\mathrm{hkl}\right\}}{\sum }A_{\left\{\mathrm{hkl}\right\}}\gamma_{\left\{\mathrm{hkl}\right\}}$$ 61
  where $A_{\mathrm{total}}$ is the total surface area of the nucleus, ⟨γ⟩ is the effective (scalar) IFE, and the sum is over all the planes with Miller indices {*hkl*} exhibited by the nucleus. Note the constraint $\underset{\left\{\mathrm{hkl}\right\}}{\sum }A_{\left\{\mathrm{hkl}\right\}}=A_{\mathrm{total}}$ . The areas $A_{\left\{\mathrm{hkl}\right\}}$ can be found using the Wulff construction.
2. The interfacial free energy between the solid and liquid (whether it is a melt or a solution) is constant (independent of the temperature) and equal to the value for an infinite plane (i.e., the curvature of the cluster can be ignored). This is usually part of the *capillary approximation*. Sometimes the failures of CNT reported in the literature correspond to failures of CNT within the capillary approximations.

Of these two approximations, the second is more relevant for the present discussion. For CNT to work within the capillary approximation, it is sufficient that the value of γ does not change much with curvature, but this is not guaranteed a priori. As discussed in section 9, we cannot ignore the effects of the curvature of the interface for clusters that are certainly not macroscopic, so that size effects become non-negligible (see ref 439, although it focuses on nucleation in liquid–vapor systems). Simulations by Montero de Hijes et al.^440^ using a hard-sphere model and a spherical solid cluster have shown that the interfacial free energy is a function of the size of the cluster (see the discussion of eq 66 in section 9). The same results were found for other systems (Lennard-Jones, water) for values of γ obtained from seeding simulations.^114,185,186^ These findings also suggest that when using CNT expressions to fit experimental results to the nucleation rate, the value of γ obtained from the fit includes curvature effects and does not correspond to the value of γ of a planar interface. In recent years, simulations using seeding methods have become increasingly popular because they offer a way to avoid the problems associated with the capillary approximation.^189,190^

An illustration of the problems encountered in the comparison of experimental and calculated values for the solid–liquid IFE is shown in Table 5, which gives a (not comprehensive) set of experimental and calculated values for the case of calcium carbonate. Two measurements of the heat of immersion (*q*_imm_ = γ_*sx*_ – γ_*sv*_) have been omitted^452,453^ because they imply a negative value for γ_*sx*_ for any reasonable estimate of the surface-vapor free energy, γ_*sv*_. In addition, we omitted the value reported in ref 454 because it is based on the dubious estimate of the free energy given by ref 451. Finally, the value from ref 447 seems unreasonably large; in fact, Wang et al.^391^ have argued that it is so large that, if correct, the nucleation of calcite would never be seen. The spread of the values in experimental numbers (55–170 mJ m^–2^) is similar to that seen in the NaCl values, once the unreliable values are removed. The set of calculated values also have some issues. The three values labeled “internal energy” assume that the configurational energy is a reasonable proxy for the enthalpy and further that the enthalpy is a reasonable proxy for the free energy (i.e., that the entropic contribution is negligible). Bruno et al.^451^ do attempt to estimate the entropic contribution, but their whole calculation assumes that a continuum approximation can be used to describe the water. The calculated free energy from ref 245 does not suffer from these problems, but there is the inevitable question of the accuracy of the force field, since the value they obtain is at the high end of the range of experimental values. However, this comparison assumes that the capillary approximation holds, which, as discussed above, is questionable. This suggestion is reinforced by the recent work of Darkins et al.,^455^ who used the first nucleation theorem and experimental nucleation rates under a wide range of conditions to determine the number of formula units in the critical cluster. The low values obtained (an average of about 10 formula units) are small enough to rule out prenucleation cluster pathways, and the lack of dependence of the values on the saturation index rules out the capillary approximation.

**Table 5:** Solid-Solution (*γ*_*sx*_) Interfacial Free Energies in mJ m^–2^ for CaCO_3_ (Calcite)^a^

	γ_*sx*_	face	technique
Söhnel and Mullin 1983 ^441^	98	average	expt: homogeneous nucleation
Liu and Lim 2003 ^442^	170	average	expt: homogeneous nucleation
Ro̷yne et al. 2011 ^443^	150	(101̅4)	expt: subcritical cracking
Jańczuk et al. 1986 ^444^	98	(101̅4)	expt: contact angle
Okayama et al. 1997 ^445^	72	(101̅4)	expt: contact angle
Hadjittofis et al. 2021 ^446^	55	(101̅4)	expt: inverse gas chromatography
Forbes et al. 2011 ^447^	1480(210)	average	expt: calorimetry
DeLeeuw and Parker 1998 ^448^	160	(101̅4)	calc: internal energy
Duffy and Harding 2004 ^449^	140	(101̅4)	calc: internal energy
Kvamme et al. 2009 ^450^	288	(101̅4)	calc: internal energy
Bruno et al. 2013 ^451^	412(20)	(101̅4)	calc: free energy (estimate)
Armstrong et al. 2024 ^245^	205	(101̅4)	calc: free energy

a Numbers in parentheses indicate the estimated error on the last digit(s) shown.

Despite its shortcomings, CNT continues to provide the framework through which much experimental work continues to be analyzed. No other theory combines its simplicity and practical utility. However, even if corrections are made to account for curvature effects, there is an additional issue: central to CNT is the assumption that the nucleation pathway is characterized only by the size of the cluster, with its ordering being identical to that of the final bulk phase. This discounts the increasing volume of evidence for the importance of clusters (of varying density and composition including dense amorphous phases) in many systems (see ref 456 for a discussion of prenucleation clusters and refs 168 and 457 for amorphous phases). Therefore, many authors have concluded that it is time to look for an alternative approach. We cannot do justice here to the considerable literature on this topic in recent years. Gebauer et al.^458^ have produced an interesting map of the territory. Authors such as Kashchiev^459^ and Jia et al.^460^ continue to argue for CNT-based approaches. A group of theories, the so-called “mesoscopic nucleation theories” ^461–464^ have been advanced to remedy the most fundamental deficiencies of CNT. The simplest versions of these theories add a second order parameter to represent the mean inner density of the cluster, thus permitting density fluctuations to evolve independently of the cluster size. However, such theories do not produce the simple analytic connection between the thermodynamic nucleation barrier and the interfacial free energy found in the classical theory. Here, our main contribution to this topic is the revision of the concepts of CNT, when the assumption of planarity of the interface between the solid and liquid phases is removed. This is discussed in the next section.

## 9 Thermodynamics of Curved Interfaces: An Approach to Nucleation

As discussed above, the capillarity approximation, which is often considered in CNT, frequently fails. Therefore, any attempt to apply CNT to probe interfacial properties must consider the effect of curved surfaces. Curved solid–liquid interfaces play a crucial role in various processes such as crystal nucleation from melts or solutions,^392,465^ nanoparticle sintering,^466,467^ and liquid storage in porous media.^468,469^ The limited development of the characterization of curved interfaces is very likely due to the increased complexity and challenges these systems pose. Experimentally, setting up a system where a curved interface is stable is difficult, if not impossible, making the direct measurements of the interfacial properties impractical. However, over the past two decades, computer simulations have significantly contributed to clarifying key thermodynamic aspects of solid–liquid curved interfaces. Here, we focus on the spherical interface formed by a solid nucleus (indicated by the usual letter *s*) surrounded by a molten phase (identified by the letter *l*) in a single-component system. Figure 8 shows a snapshot of a configuration that is stable in the NVT ensemble (Under certain conditions, a solid nucleus can remain stable within its melt in various ensembles, e.g., NVT , NVE , or *NPH*,^470,471^ as demonstrated in computer simulations^472–477^ ). We shall now present a brief introduction to the thermodynamics of curved interfaces at equilibrium.

**Figure 8:**
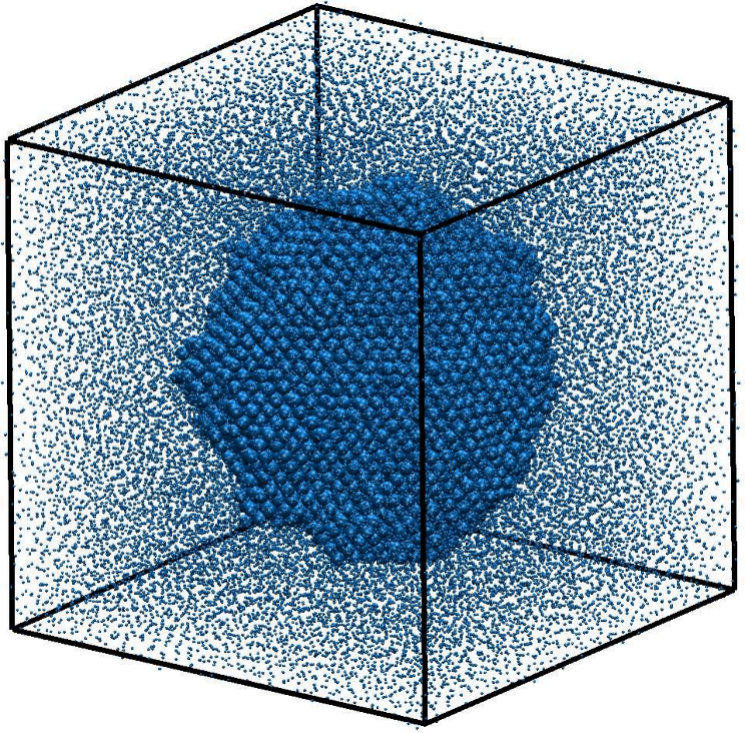
Solid cluster of hard spheres is shown in equilibrium with the surrounding melt in the NVT ensemble. For clarity, liquid particles are depicted at a reduced size. The system is in thermodynamic equilibrium, meaning that molecular motion ensures both temperature and chemical potential (but not pressure) are uniform throughout the system. This figure was reproduced with permission from ref 478. Copyright 2022 American Institute of Physics.

### 9.1 Thermodynamics of Curved Interfaces

Because the system is in equilibrium, the IFE, γ_*sl*_, can be defined (for a system at constant volume) from^155,479^

$$F=N\mu -P_{s}V_{s}-P_{l}V_{l}+\gamma_{sl}A$$ 62

where *F* is the Helmholtz free energy, *N* is the total number of particles, μ is the chemical potential, A is the interfacial area, *P*_*s*_ and *P*_*l*_ are the pressures of the solid and liquid phases, respectively, and $V_{s}$ and $V_{l}$ are their respective volumes (with the total volume given by $V=V_{s}+V_{l}$ ). It is important to realize that eq 62 applies to both planar and curved interfaces [Equation 62 is usually the one reported in the study of curved interfaces, but we want to stress that it is consistent with the thermodynamic relations reported in other sections of this review. In particular, it is equivalent to eq 39 in assuming that there is no excess volume at the interface, $V^{XS}=0$ (see also the discussion regarding eqs 3 and 4), *c* = 1, and *P*_*s*_ = *P*_*l*_, which is true for a planar interface as considered in previous sections]. For further extensions to other curved interfaces and multicomponent systems, see refs 480 and 481.

According to Gibbs, we should assume that the system consists of a solid up to a certain Gibbs dividing surface, and of a liquid beyond that. Is the value of γ_*sl*_ affected by the choice of the location of the dividing surface? For a planar interface, the answer is no. In this case, since *P*_*s*_ = *P*_*l*_, moving the interface does not change the area A and thus γ_*sl*_ remains invariant to the choice of the dividing surface. However, for curved interfaces, the value of γ_*sl*_ *does* depend on the choice of the dividing surface. In this case, *P*_*s*_ and *P*_*l*_ differ, and so changing the dividing surface alters $V_{s}$ , $V_{l}$ , and A . Therefore, we must express the IFE of a given thermodynamic state with a curved interface as γ[R] , where the brackets imply that γ_*sl*_ changes with the choice of the dividing surface (for the rest of this section we drop the subscript *sl* for notational simplicity).

Since *F* (and similarly μ) must remain invariant to the choice of the dividing surface, we can take the notational derivative of eq 62 with respect to the radius of the cluster, R , and set it to zero, yielding:

$$\Delta P=P_{s}-P_{l}=\frac{2\gamma }{R}+\left[\frac{d\gamma }{dR}\right]$$ 63

The derivative in brackets is a *notational* derivative, describing how γ changes with the *chosen* position of the dividing surface for a given system. Gibbs suggested a particular choice, known as the surface of tension, where γ[R] reaches its minimum. The radius at which this minimum occurs is denoted $R_{C}$ , and the corresponding value of γ at this minimum is γ_C_. By rewriting eq 63 at the surface of tension, we obtain the Young–Laplace equation:

$$\Delta P=\frac{2\gamma_{C}}{R_{C}}$$ 64

Therefore, the Young–Laplace equation only holds when using the radius at the surface of tension and the corresponding value of γ at that surface. This highlights the fact that when reporting values of γ for curved interfaces, it is essential to specify the choice of the dividing surface. For example, another commonly used dividing surface is the equimolar surface, $R_{E}$ , defined by $N=\rho_{s}V_{s}\left[R_{E}\right]+\rho_{l}V_{l}\left[R_{E}\right]$ , where the number of excess surface molecules is zero. However, the Young–Laplace equation does *not* apply to this surface, and the value of γ = γ_E_ for this surface is higher than γ_C_.^155,482,483^ A general expression can be used to describe γ at any R if γ_C_ and $R_{C}$ are known:

$$\gamma \left[R\right]=\gamma_{C}\frac{2R^{3}+{R_{C}}^{3}}{3R^{2}R_{C}}$$ 65

which has been extended to account for cylindrical interfaces in ref 478. Equation 65 describes *notational* changes in γ, that is, changes in γ for a given solid cluster due to changes in the arbitrary choice for the dividing surface. However, γ_C_ changes with *real* changes in the size of the solid cluster as will be discussed in the next subsection.

### 9.2 Changes in γ_*C*_ with the Size of the Cluster: Tolman’s Equation

The quantity γ_C_ can *only* be defined when the system is at equilibrium, (i.e., when *T* and μ are homogeneous and the divergence of the pressure tensor is zero). $R_{C}$ is not an independent variable, since for each value of *T* and μ there is a unique value of $R_{C}$ at which the cluster is in equilibrium with the liquid (For hard spheres only μ is required, but this is not the case for Lennard-Jones or other thermal systems). In Figure 9, we illustrate the variation of γ_C_ with real changes in the radius of $R_{C}$ for the three benchmark systems considered in this review: hard spheres, Lennard-Jones, and water (considering two water models, mW^363^ and TIP4P/Ice^376^). As shown there, γ_C_ changes with the size of the spherical solid cluster: a real, physical change, not merely a notational one. The capillarity approximation should be reconsidered in the light of the thermodynamic description of curved interfaces, not just for liquid–solid interfaces but also for liquid–liquid interfaces. Recent simulation evidence overwhelmingly supports the perspective presented here.^327,484–488^

**Figure 9:**
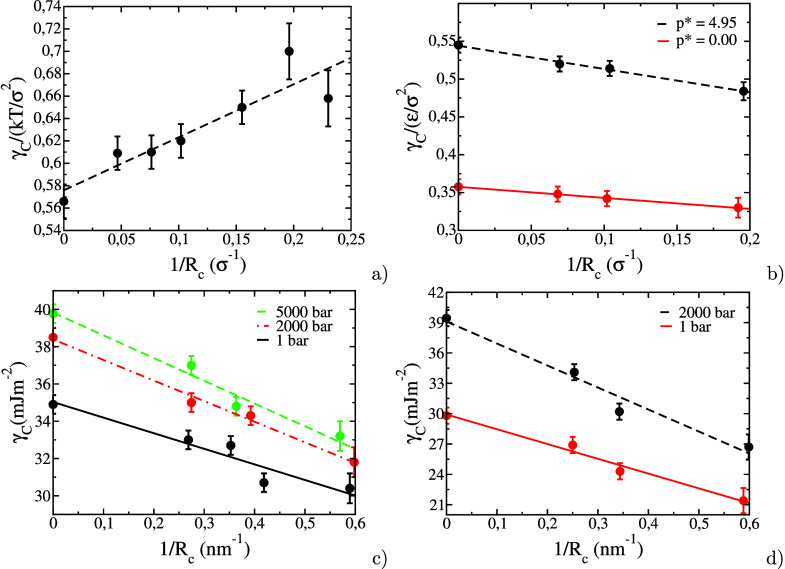
Variation of γ_*C*_ with the radius of the cluster $1/R_{C}$ for (a) hard spheres, (b) Lennard-Jones (two isobars), (c) mW^363^ model of water (three isobars), and (d) TIP4P/Ice (two isobars). This figure was reproduced with permission from ref 327. Copyright 2019 American Institute of Physics.

The change of γ_C_ with curvature can be described by an expression first proposed by Tolman^489^

$$\gamma_{C}=\gamma^{0}\left(1-\frac{2\partial }{R_{C}}\right)$$ 66

where γ^0^ is the value of γ_C_ at the planar interface and ∂ has the units of length. When relating γ_C_ at a specific *T* and *P* ( which define uniquely both the value of chemical potential μ and the radius at the surface of tension of the equilibrium cluster $R_{C}$ ) to the value at a planar interface, one can maintain constant *P* (moving along an isobar) or keep *T* constant (moving along an isotherm). Tolman chose the isothermal path, which is why ∂ is referred to as the Tolman length (in the original work^489^ the Tolman length is given the symbol δ). Although originally proposed for liquid–liquid interfaces, the Tolman equation has also been found to be useful for crystalline nuclei.^327,475,490^ The inclusion of higher-order (quadratic) terms in the expansion has been discussed,^491^ along with its application to isobaric paths.^492,493^ The physical interpretation of γ^0^ in eq 66 for the solid–liquid interface remains somewhat ambiguous. As $R_{C}$ approaches infinity, the system converges to a planar interface; yet, as we discussed earlier (see section 3.1), the value of γ for a planar interface depends on the specific crystallographic plane considered.^494^ Furthermore, as a solid cluster grows, it tends to form facets rather than a smooth spherical interface.^473^ In practice, the value of γ^0^ is generally close to the average of γ_{*hkl*}_ for planes with lower Miller indices {*hkl*}, but more research is necessary to fully understand this phenomenon.

### 9.3 Young–Laplace Equation for Solid–Fluid Curved Interfaces Reconsidered

So far, *P*_*l*_ has referred to the pressure of the external liquid phase. However, what value should be used for *P*_*s*_ (the pressure of the solid phase) in eq 62? In Figure 10, the tangential and normal components of the pressure tensor (for a pseudo hard sphere system) are displayed as functions of the distance from the center of the solid cluster of Figure 8. The pressure inside the solid cluster is lower than that in the external liquid phase. This result was initially observed in Lennard-Jones solid clusters^477^ and has been corroborated for pure hard spheres.^495^ Moreover, *P*_*s*_ was implicitly described in terms of the density of the nuclei of the hard spheres.^496–498^ This anomalous behavior (*P*_*s*_ < *P*_*l*_) has no analogy in liquid–liquid interfaces, where the pressure of the internal spherical liquid phase is always greater than that of the external liquid phase. The lower pressure of the solid, as indicated in Figure 10, leads, according to eq 64, to a negative value of γ_C_, which is a nonphysical result.

**Figure 10:**
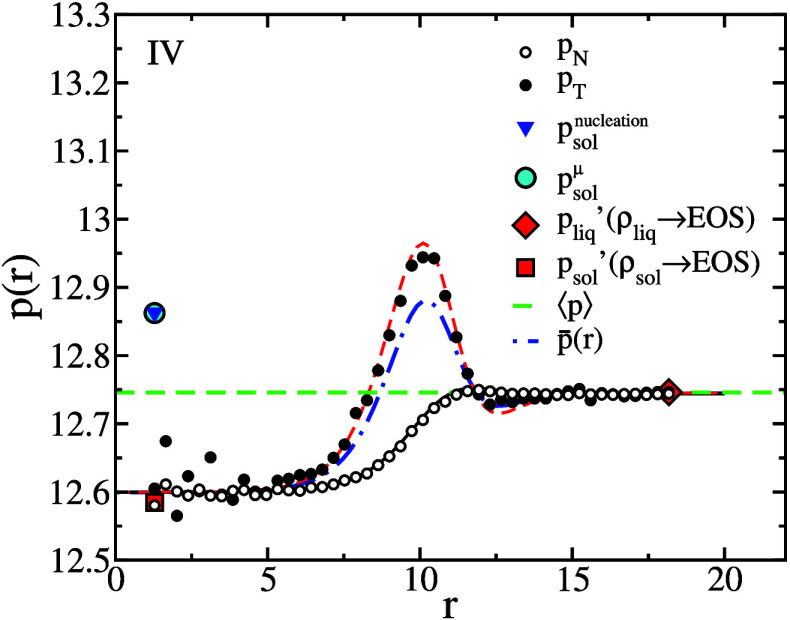
Normal *P*_*N*_ and tangential *P*_*T*_ components of the pressure tensor for a spherical solid cluster of pseudo hard spheres in equilibrium with the liquid at constant *N*, V , and *T*. The pressure components are shown as a function of the distance (*r*) to the center of mass of the cluster. We refer for the exact definition of all the other symbols to the original ref 476. This figure was reproduced with permission from ref 476. Copyright 2020 American Institute of Physics.

Tolman, following Gibbs’ original work, suggested the solution to this anomaly, although initially only in the context of small liquid droplets. Following Tolman and Gibbs, we define *P*_*s*_ as the pressure of a perfect bulk solid (without defects or strain) that has the same chemical potential as the external liquid phase. This definition of *P*_*s*_ is referred to as the thermodynamic pressure *P*_*s*_^μ^ (i.e., the pressure of a perfect bulk solid with the same chemical potential μ as the external liquid phase). Recent findings indicate that this definition should be applied not only to small clusters but also to any spherical solid cluster, regardless of its size. As in the previous formalism, the properties of a reference solid are utilized rather than those of the actual solid. The use of reference systems is common in thermodynamics. For example, the reference state of the solute in the thermodynamics of mixtures follows similar principles. These properties of this “reference solid” are necessary for defining γ_*sl*_ in a curved solid–liquid interface (when using eq 62).

How is it possible to have two solids with the same value of μ, one with *P*_*s*_^μ^ and the other with the actual mechanical pressure of the cluster, *P*_*s*_^mech^? The first represents a reference bulk solid without defects at *P*_*s*_^μ^, while the second is the actual solid, which contains vacancies and/or strain, but maintains the same chemical potential at *P*_*s*_^mech^ as the external liquid phase. These internal degrees of freedom could be incorporated into a thermodynamic description of the solid,^499^ but the solution of using a reference bulk solid is both simple and elegant. The extension of the Gibbsian formalism to account for the additional state variables arising from the possibility of strained states and defects within the spherical interface was addressed by Mullins,^500^ who already noted that the actual nucleus is not bulk in nature. This approach was later applied in the context of simulations of hard-sphere systems.^496,497^ Mullins expanded the solid variables in terms of unit cell volume, number of unit cells, and number of components per unit cell. Surprisingly, the approach of Mullins has received little attention since it was suggested in 1984. However, it has inspired recent work on the statistical mechanics of a crystalline nucleus of hard spheres in liquid,^495^ leading to conclusions in agreement with ref 476 (i.e., *P*_*s*_^mech^ < *P*_*s*_^μ^ in that system) while providing more insight into the interfacial stress of the system and the role of vacancies. Some authors have used a bulk solid without defects at *P*_*s*_^mech^ as the reference state for the solid. However, this choice should be avoided^501,502^ because this reference solid will have a chemical potential different from that of the liquid. In our view this is not appropriate, as the system is at equilibrium and the chemical potential must be homogeneous.^482,503^

Although we have focused on the Young–Laplace equation, other equations commonly used in the literature are influenced by similar reasoning, namely that the value of γ varies with R and also depends on the choice of the dividing surface. This is also true for the Gibbs–Thomson equation, which describes the freezing point depression under confinement. This equation, like the Young–Laplace equation, incorporates γ for a curved interface.^504^ A source of confusion may arise from the fact that in Gibbs' formalism, the value of the interfacial free energy depends on the choice of the dividing surface when the interface is curved. However, an approach that does not come with this limitation and was previously applied to solids is that proposed by Cahn,^296^ which was thoroughly discussed in section 5.7. However, the Cahn approach has rarely been applied to the curved solid–liquid interface.^303,304,477^

### 9.4 Connecting Equilibrium and Nucleation

So far we have discussed the thermodynamic aspects of the curved solid–fluid interface at equilibrium. However, there is an important connection between equilibrium and nucleation. Let us start with two simple questions: can these stable spherical clusters (in the NVT ensemble) provide insights into nucleation? What happens when we switch to the *NPT* ensemble using the average pressure obtained during the NVT simulation? In Figure 11, we show that upon changing the ensemble, these stable clusters either melt or grow, with each process occurring approximately half of the time. In other words, they behave as critical clusters. They are at equilibrium in both ensembles (i.e., both temperature and chemical potentials are homogeneous); however, the equilibrium is *stable* in the NVT ensemble and *unstable* in the *NPT* ensemble. This is represented in Figure 12, where the same system is at a minimum in *F* in the NVT ensemble and at a maximum in *G* in the *NPT* ensemble (or Ω in the grand-canonical ensemble).^505^ Figures 11 and 12 have significant implications because they connect the thermodynamics of curved interfaces with the nucleation realm.

**Figure 11:**
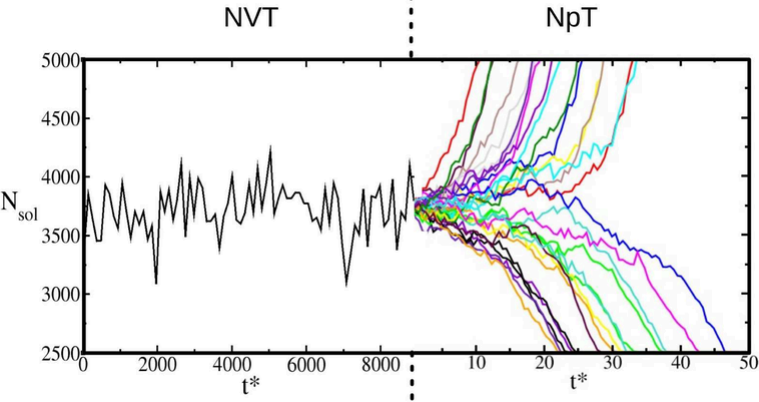
Trajectories in the *NPT* ensemble from a configuration of the stable solid cluster in the NVT shown in Figure 8. Results shown were obtained for the hard-sphere potential introducing a spherical solid cluster of size *N*_sol_ in the fluid phase. This figure was adapted with permission from ref 475. Copyright 2020 American Chemical Society.

**Figure 12:**
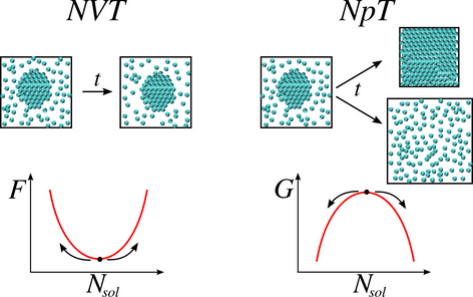
Sketch showing a stable solid cluster in the NVT ensemble (minimum in *F*) corresponding to a saddle point in the *NPT* ensemble corresponding to a critical cluster. This figure was reproduced with permission from ref 475. Copyright 2020 American Chemical Society.

We will now summarize some of the notation and ideas reported in section 8 for nucleation, as we want to discuss CNT in light of relaxing some of the approximations considered in that section. In particular, we will highlight the role of the curved interface, which was neglected in section 8. We start by writing the expression of the nucleation rate *J*_CNT_ in a slightly different form from that discussed in section 8. The main difference is the expression of the Zeldovich factor (see section 8, eq 57):

$$J_{\mathrm{CNT}}=\rho_{l}\sqrt{\frac{\left|\Delta G_{\mathrm{crit}}^{\prime \prime }\right|}{\left(2\pi k_{B}T\right)}}\phi^{+}\exp\left(-\frac{\Delta G_{\mathrm{crit}}}{k_{B}T}\right)=\rho_{l}Z\phi^{+}\exp\left(-\frac{\Delta G_{\mathrm{crit}}}{k_{B}T}\right)$$ 67

where again ρ_*l*_ represents the number density of molecules in the liquid phase, *Z* is the dimensionless Zeldovich factor, ϕ^+^ is the attachment rate (which has units of the inverse of time), and Δ*G*_crit_^*″*^ indicates the curvature (that is, the second derivative) of the free energy profile at the critical maximum, Δ*G*_crit_.

Computer simulations enable the testing of CNT, since the quantities in eq 67 can be evaluated numerically. Pioneering studies by Frenkel et al.^506–509^ demonstrated that the free energy profile as a function of the size of the largest solid cluster could be determined using the umbrella sampling technique. From this free-energy profile, one can determine Δ*G*_crit_ and *Z*, while ϕ^+^ can be obtained from additional simulations that estimate the diffusive behavior of the cluster at the top of the barrier. This technique has proven to be highly successful in estimating the values of *J*_CNT_ for various systems, including hard spheres,^485,510^ Lennard-Jones,^511^ water (mW),^511,512^ silicon,^513^ and sodium chloride,^514^ among many others. Such estimates of *J*_CNT_ have been found to be generally quite accurate (except in the case of two-step nucleation processes) and are not sensitive to the choice of the order parameter used to classify molecules as liquid or solid (the definition of the order parameter is given in section 4.2). Moreover, the umbrella sampling technique does not require the prediction or definition of any value for the interfacial free energy between the liquid and the solid.

However, since equilibrium clusters in NVT are critical in *NPT*, it is possible to connect the thermodynamics of curved interfaces in equilibrium with Δ*G*_crit_, that is, with the Gibbs free energy difference (now with constant *N*, *P*, and *T*) between a system with a critical cluster (given by $F+P_{l}V$ for inhomogeneous systems) and that of a homogeneous liquid (given by *Nμ*). By subtracting both terms, one obtains the following (using the equations of the thermodynamics of curved interfaces of this section):

$$\Delta G_{\mathrm{crit}}=\gamma A-V_{s}\left(P_{s}^{\mu }-P_{l}\right)$$ 68

This equation is exact. As described above, the values of γ, A , and $V_{s}$ for the critical cluster depend on the choice of the dividing surface, but Δ*G*_crit_ does not depend on this choice. By setting the notational derivative of Δ*G*_crit_ to zero, one recovers eq 63. By selecting the value of R (i.e., $R_{C}$ at which γ is the minimum γ_C_), one recovers the Young–Laplace equation (eq 64). By using the Young–Laplace equation, one can rewrite eq 68 as

$$\Delta G_{\mathrm{crit}}=\frac{1}{3}\gamma_{C}A_{C}=\frac{1}{2}V_{C}\left(P_{s}^{\mu }-P_{l}\right)=\frac{16\pi {\left(\gamma_{C}\right)}^{3}}{3{\left(P_{s}^{\mu }-P_{l}\right)}^{2}}$$ 69

which is an exact result ( $A_{C}$ and $V_{C}$ being the area and volume of the solid critical cluster evaluated at the surface of tension), already known to Gibbs. The free energy barrier for nucleation (which is needed to determine *J* within CNT) is therefore one-third of the interfacial free energy of the critical solid cluster (when choosing the radius at the surface of tension, which is always the recommended choice). This establishes the connection between nucleation and γ through Δ*G*_crit_. Whereas eq 68 is correct for any choice of the dividing surface of the critical/equilibrium cluster, eq 69 is correct only when choosing the surface of tension as the dividing surface of the critical/equilibrium cluster.

Some confusion about eq 68 should be clarified. Both eq 68 and eq 69 are exact (that is, they contain no approximations), but they are exact only for the critical cluster (which is at equilibrium and where thermodynamics holds), and not for a cluster of arbitrary size. They are obtained by using a rigorous thermodynamic formalism. It is tempting to assume that eq 68 is valid for solid clusters of any size ( R ) and write

$$\Delta G=\gamma A\left(R\right)-V_{s}\left(R\right)\left(P_{s}^{\mu }-P_{l}\right)$$ 70

but this is not exact, as clusters of sizes different from that of the critical cluster are not at equilibrium and therefore thermodynamics does not hold. However, if γ and *P*_*s*_^μ^ – *P*_*l*_ are assumed to be constant (and the value of γ_C_ is adopted for γ) and the first derivative of Δ*G* with respect to R is set to zero, then the correct eq 69 is recovered. This suggests that the formalism of the thermodynamics of curved interfaces can be avoided, but the result is not rigorous and, in fact, leads to incorrect values if not applied to the critical cluster. Therefore, eq 70 should be used (if at all) with great care.

However, the curvature at the top of the free energy profile, which is not available from thermodynamic reasoning and is needed to evaluate *J*_CNT_, can be estimated by using eq 70. Indeed, the latter equation can be used to describe the free energy profile at the top of the barrier, making it possible to obtain an estimate of the Zeldovich factor *Z*:

$$Z=\sqrt{\frac{\left(P_{s}^{\mu }-P_{l}\right)}{8\pi^{2}k_{B}T{\rho_{s}}^{2}{R_{C}}^{3}}}=\sqrt{\frac{\gamma_{C}}{4\pi^{2}k_{B}T{\rho_{s}}^{2}{R_{C}}^{4}}}$$ 71

where ρ_*s*_ is the number density of the solid. As shown, γ also contributes to this approximate expression of the Zeldovich factor, although its primary impact on nucleation arises through the free energy barrier. It is important to note that *Z* is dimensionless. The use of (*P*_*s*_^μ^ – *P*_*l*_) in the thermodynamic formalism is recommended; this formulation was proposed by Gibbs and integrates naturally into the thermodynamics of curved interfaces.^488,515^ Although it is not difficult to evaluate (*P*_*s*_^μ^ – *P*_*l*_), its value is sometimes computed directly with an approximation. If we assume that the solid is incompressible (which implies that its density does not change with pressure), we can show that (*P*_*s*_^μ^ – *P*_*l*_) ≃ ρ_*s*_Δμ, where Δμ is the difference in chemical potential between a bulk liquid and a bulk solid at the pressure of the liquid phase *P*_*l*_. By making this substitution, two equations presented earlier in this review are obtained (see eqs 13 and 12 in section 4.2), with the origin of the derivation now clearer:

$$\Delta G=\gamma_{C}A-V_{s}\rho_{s}\Delta \mu =\gamma_{C}A-N_{s}\Delta \mu$$ 72a

$$\Delta G_{\mathrm{crit}}=\frac{16\pi {\gamma_{C}}^{3}}{3{\left(\rho_{s}\Delta \mu \right)}^{2}}$$ 72b

where *N*_*s*_ is the number of solid particles in the largest solid cluster. The key message of this section is that *J*_CNT_ can be estimated accurately if Δ*G*_crit_ is determined correctly. Δ*G*_crit_ is equivalent to one-third of the product of γ_*C*_ and $A_{C}$ of the critical cluster, and this relationship is exact.

In umbrella sampling, as well as in metadynamics, Δ*G*_crit_ is computed directly without relying on specific thermodynamic definitions. In the seeding technique, the point at which a cluster becomes critical is determined, which remains independent of the chosen order parameter. To estimate the radius of the spherical cluster at the surface of tension (i.e., $R_{C}$ ) at which the formalism holds, it is essential to use a robust order parameter. This should yield a value for *N*_crit_ (the number of solid particles in the critical cluster), leading to accurate estimates of $R_{C}$ through the following relationship:

$$R_{C}={\left(3N_{\mathrm{crit}}/\left(4\pi \rho_{s}\right)\right)}^{1/3}$$ 73

Although ϕ^+^ should be determined using computer simulations, a fairly accurate estimate can be obtained in the case of the freezing of a pure substance as ϕ^+^ = 24*DN*_crit_^2/3^/λ^2^ where *D* is the diffusion coefficient of the molecules in the liquid phase and λ is of the order of a molecular diameter.

We conclude by presenting results for the nucleation rate. In Figure 13(a), we show the estimates of the nucleation rate for hard spheres, while Figure 13(b) displays the results for water using the mW model.^363^ These estimates are obtained from eq 67 using computer simulations to determine ρ_*l*_, ϕ^+^, a suitable order parameter to estimate the size of the critical solid cluster *N*_crit_, and consequently $R_{C}$ . The results are compared with those obtained from umbrella sampling, brute-force simulations, and forward flux sampling. As illustrated, eq 67 accurately estimates *J* for these two systems.

**Figure 13:**
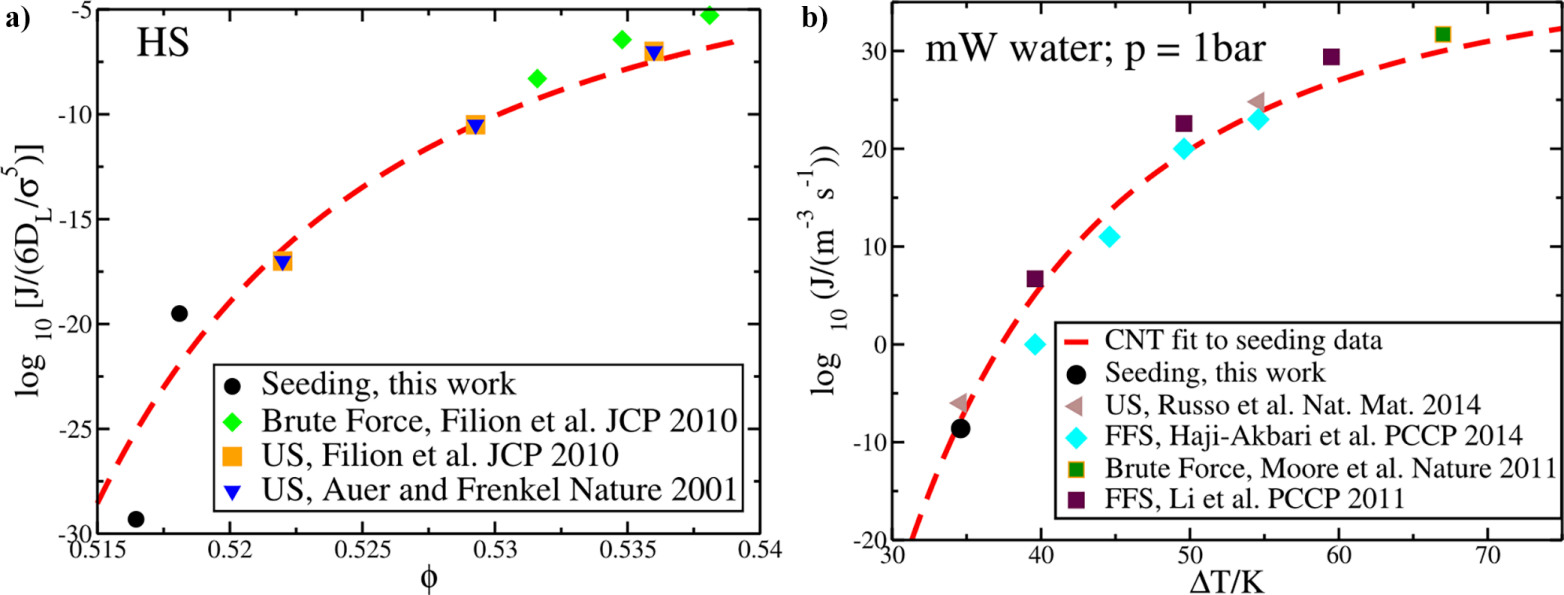
Nucleation rates of (a) HS and (b) the mW model of water obtained from seeding compared to results obtained from brute force simulations, umbrella sampling, and forward flux sampling. This figure was reproduced with permission from ref 511. Copyright 2016 the American Institute of Physics.

In summary, when the capillarity approximation is not considered (which is even poorly defined for solids, since for planar interfaces at coexistence, the value of γ depends on the specific plane), and when utilizing input from simulations, good estimates of *J* can be obtained. For many systems, having the correct value of γ_C_ is crucial to produce reliable estimates of *J*_CNT_. Ultimately, the key lies in employing an order parameter that provides an informed estimate of the radius at the surface of tension of the critical cluster, $R_{C}$ .

## 10 Concluding Remarks

The purpose of this review was threefold:

1. To present a discussion of the IFE for solid–liquid systems from a thermodynamic point of view, highlighting the differences from the liquid–liquid case.
2. To use this to make the case that more refined models are needed in molecular dynamics simulations to determine solid–liquid IFEs than those used for the liquid–liquid case.
3. To give an overview of such models, which we categorized in “direct” and “indirect” methods.

Despite being a long-standing problem (it has been about 150 years since Gibbs took it up), interfaces involving solids continue to pose several challenges on both the theoretical and computational side as shown by the vast literature available on different aspect of solid–liquid interfaces (to which we refer the interested reader), such as electronic properties,^516,517^ electrocatalytical processes,^518,519^ formation of the electric double layer,^79,80,520^ and adsorption of macromolecules.^521,522^

The challenges faced by those attempting to calculate IFEs for solid–liquid interfaces arise from basic features of crystalline solids (their anisotropy and their ability to support stress) requiring a level of sophisticated treatment beyond that required by their liquid–liquid counterparts. This explains why those attempting to calculate the interfacial free energy when solids are involved so often turn to thermodynamic integration methods. In turn, the fact that so many methods are employed means that there is no such thing as *the* method to calculate the solid–liquid interfacial free energy (as discussed for liquid–liquid systems), but rather there are several different methods, each with their own merits and difficulties, which need to be chosen based on the particular problem considered (see the extended discussion in appendix B).

It is therefore not surprising that the analysis and methodologies required to determine interfacial free energies in solid–liquid system have had limited appeal within the community. The need to learn different methodologies and adapt a piece of software for the particular systems considered is a great source of inertia, effectively resisting the adoption of such techniques. A newcomer in the field who wants to determine the interfacial free energy of a liquid–liquid system will find several resources for this (relatively simple) calculation, which is now routinely done in widely used MD software packages. The same newcomer facing the problem of determining the interfacial free energy for a solid–liquid system faces a steep learning curve and a plethora of different software, each tweaked for the calculation in a particular system. As others have already pointed out^523^ (and the authors of this review agree), knowledge dissemination is important and it must include the creation of a well-documented and maintained piece of software available to the community. For this reason, part of the scientific endeavor in this field should be devoted to make it easier to deploy these methodologies. In this spirit, the authors of this review (working on different models related to solid–liquid interfaces) have published documented software complemented by examples on how to use the methodologies presented here (see the mold technique^524^ with repository available at ref 525, Cleaving^526^ with repository available at ref 527, and the Einstein crystal model with repository available at ref 528).

A topic related to determination of interfacial free energies in systems involving a solid phase that we did not discuss in this review is the description of solid–solid interfaces. The reason is not that they are uninteresting, as they have many important applications, from solid-state batteries^529,530^ to geophysics.^531^ However, as the passage from the study of liquid–liquid interfaces to solid–liquid ones is dark and full of terrors, moving to solid–solid systems further increases the complications, particularly for heterointerfaces, which would warrant a review on their own. This review is already long enough. The task of determining interfacial properties for solid–solid systems using MD simulations is still in its infancy (the interested reader can find some examples in refs 532 and 533), but we are sure that the methods and ideas presented here will set the foundations on which new models for the treatment of these more complicated systems will be built.

## Appendix A: Thermodynamic Integration

Because the direct methods presented in section 5 are mainly based on the thermodynamic integration technique, we include here a brief section to recall the main features of this methodology, leaving all the details to the excellent references available.^214,534^

As we have introduced in eq 9, γ (under certain conditions) is linked to the difference in the Helmholtz free energy between the state of the system with an interface and the state of the system without it. Unfortunately, we cannot directly calculate the Helmholtz free energy from the MD simulation, as the free energy is not the average of some function of phase space, but it is related to the canonical partition function Q(N,V,T) :

$$F=-k_{B}T\ln Q\left(N,V,T\right)$$
74

with

$$Q\left(N,V,T\right)=\frac{1}{\Lambda^{3N}N!}\int \exp\left(-\frac{U\left(r\right)}{k_{B}T}\right)dr$$
75

where *U* is the configurational energy of the *N* atoms in the system, **r** = {**r**_1_, ···, **r**_*N*_} is their position in physical space, and Λ is the de Broglie thermal wavelength.

Although *F* cannot be directly obtained from MD simulations, the same does not hold true for its derivatives. For example, pressure is minus the derivative of *F* with respect to the volume of the system at constant *N* and *T* and can be readily evaluated in MD simulations, e.g., from the virial stress. This latter observation is the insight that makes it possible to calculate free-energy differences in simulations.

Let us assume that the Helmholtz free energy depends on a generic parameter λ and that the value of *F* in which we are interested corresponds to a certain value λ_fin_. In this case we can write

$$F\left(\lambda_{\mathrm{fin}}\right)-F\left(\lambda_{\mathrm{init}}\right)=\int_{\lambda_{\mathrm{init}}}^{\lambda_{\mathrm{fin}}}\left(\frac{\partial F}{\partial \lambda }\right)d\lambda$$
76

where *F*(λ_init_) is the value of the Helmholtz free energy at another point identified by λ_init_. In order to use eq 76 to compute the value of the Helmholtz free energy at λ_fin_, two conditions must be met:

1. We know at least one value of Helmholtz free energy, indicated here as *F*(λ_init_).
2. We can build a *thermodynamic path* connecting the two states identified by λ_init_ and λ_fin_.

For each point along this path, the value of $\left(\frac{\partial F}{\partial \lambda }\right)$ , which is a quantity accessible by MD simulations, is computed and used to numerically evaluate the integral in eq 76, often using a quadrature approximation. In general, the parameter λ does not need to be a physical quantity (as, e.g., the density), but it can also be a parameter on which the interactions among atoms depend: *U*(**r**;λ). This allows us to write

$$\left(\frac{\partial F}{\partial \lambda }\right)=\left(\frac{\partial }{\partial \lambda }\right)\left(-k_{B}T\ln Q\left(N,V,T;\lambda \right)\right)=-k_{B}T\frac{\partial }{\partial \lambda }\log\left[\int \exp\left(-\frac{1}{k_{B}T}U\left(\lambda \right)\right)dr\right]=\frac{\int \left(\frac{\partial U\left(\lambda \right)}{\partial \lambda }\right)\exp\left(-\frac{1}{k_{B}T}U\left(\lambda \right)\right)dr}{\int \exp\left(-\frac{1}{k_{B}T}U\left(\lambda \right)\right)dr}={\left\langle \frac{\partial U\left(\lambda \right)}{\partial \lambda }\right\rangle }_{\lambda }$$
77

where we dropped the dependence on **r** in *U*(**r**;λ). Therefore, the integrand in eq 76 is the ensemble average of the derivative of the configurational energy *U* with respect to the parameter λ. The ensemble average itself in eq 77 depends on λ and therefore does not commute with the integral in eq 78.

In the context of this review, the quantity we wish to evaluate is given by eq 9, which requires the difference of the Helmholtz free energy to determine γ, so that eq 76 becomes

$$\gamma =\frac{1}{A}\int_{\lambda_{\mathrm{init}}}^{\lambda_{\mathrm{fin}}}\left(\frac{\partial F}{\partial \lambda }\right)d\lambda$$
78

where now the initial and final points are (generally) the system without and with an interface, respectively. The integrand in eq 78 depends on the thermodynamic path λ_init_ → λ_fin_ chosen to create an interface, the choice of which is discussed in section 5 and corresponds to the different methodologies presented.

## Appendix B: Computational Tips

In this section, we provide some heuristics to help readers to use the methodologies discussed here. We will include observations and suggestions that the authors of this review hope both newcomers and seasoned users will find useful for their work with solid–liquid interfaces. The underlying condition upon which any thermodynamic integration rests is the reversibility of the thermodynamic path. In the general case, the work needed to create a new interface is $W^{XS}\geq \gamma A$ , where the equality (eq 5) holds only for a *reversible* transformation. The degree of reversibility of the thermodynamic path can be estimated by calculating the *hysteresis* of the transformation (see ref 535 for a discussion). The hysteresis is the difference between the work calculated in the forward path (where the starting point is the bulk and the end point is the system with an interface) and the backward path (the reverse direction). In a reversible calculation this difference should be zero (see e.g., Figures 2–4 in ref 224).

The different methods discussed above have different hysteresis behavior, and different strategies have to be employed to determine it. In the dry surface method, the determination of the hysteresis can become complicated since it is difficult to bring the system back to its starting point once the liquid has detached from the solid. This behavior was observed in ref 158, in which the authors could not determine the hysteresis directly. They instead calculated the work of detachment of the liquid droplet from the solid surface for different independent initial configurations, checking that the final value of the calculated work was consistent across the different cases. While the calculation of the hysteresis is related to the whole thermodynamic path (from bulk to interface and back), if the methodology chosen to determine γ can be broken down into substeps (e.g., as in the cleaving methods, which usually include four steps, see eq 18a), each step can be checked independently and countermeasures deployed to remove hysteresis.

One of the main sources of hysteresis is the liquid ordering transition that occurs when the interface is created. The creation of structural ordering in the liquid when an external potential is applied under coexistence conditions suggests that the system must cross a high free energy barrier associated with the formation of the ordered structure. The direct and reverse thermodynamic paths followed by trajectories in phase-space will therefore be different.^96,224^ One of the ways to address this problem within the cleaving approach is to modify Step 2 so that the cleaving of the liquid is done in conditions away from solid–liquid coexistence. For example, in case of the hard-sphere systems, the cleaving can be done at lower densities,^536^ while for continuous potentials it can be carried out at elevated temperatures.^224^ It is also possible to introduce modifications of the interparticle interactions near the cleaving plane.^225^

An alternative way of introducing the external potential is given by the wells (described in the mold integration and cleaving approaches discussed in sections 5.1 and 5.2). The wells capture the atoms in the liquid phase, thus promoting the formation of an ordered structure. The ability of the wells to capture atoms can be tuned by changing their depth and attraction range. However, since each well may capture only one atom, any tuning must be done with care. Another way to reduce hysteresis is to modify the thermodynamic path so that the ordering transition is less abrupt (see ref 224 for a scheme that can do this but at the cost of longer and more complicated calculations).

Another major complication in the creation of stable, equilibrium solid–liquid interfaces arises because *N*_*i*_, the number of particles of each type, must be conserved. This constrains the choice of the initial density and composition of the bulk fluid, especially for multicomponent systems. The initial bulk density should be slightly different from the desired final coexistence density because particles will move between interface and bulk, changing the bulk density. If the final density and composition of the bulk fluid are not correct, then the pressure of the fluid will be different from the coexistence value, causing the crystal to expand or contract along the direction normal to the interface. Since the lattice spacing in the transverse (*xy*) directions remains fixed, this can lead to significant excess stress in the bulk crystal. Examination of the excess bulk crystal stress provides, therefore, a way to assess the deviation of the final system from coexistence equilibrium.

For single-component systems, the process of creating an equilibrium interface is straightforward.^537^ The simulation setup begins with the separate equilibration of the bulk crystal and fluid phases, with the density of the fluid being set to be slightly greater than the target coexistence bulk value. The crystal and fluid are then placed next to each other with a small gap between them. The gap is necessary to avoid initial high-energy interactions. As the simulation progresses, this gap will fill while lowering the bulk density. The initial density and/or the gap size are then adjusted to ensure zero excess stress in the bulk crystal region. Multicomponent systems add complexity because differences in the relative interfacial adsorption for different components require that, in addition to the density, the initial composition must also be optimized to yield a stress-free crystal. For two-component (binary) systems, the problem is tractable and has been illustrated for both binary hard sphere^206,312^ and metal alloy^124^ crystal–melt interfaces.

We have presented several different ways to calculate the IFE. In principle, they should eventually agree on the value of the IFE. However, the different choices of thermodynamic paths and implementation imply that, when they are used in a calculation, some may be more appropriate than others for systems under study. In some cases, several methods could be used, so that further considerations, such as computational cost and availability of software, may become a factor. Here, we will give a quick assessment to help the reader choose the approach best suited to their problem.

The cleaving model was one of the first methodologies to be proposed. Because it breaks down the thermodynamic path into several steps, it allows a precise control over the transformation of the system along the thermodynamic path. The steps comprising the method can be further split in substeps if required (for instance, ref 224 included two more steps to separate the contribution of the long-range component of the electrostatic interactions from the short-range ones in the formation of the interface). Furthermore, the standard deviation of the results obtained is often better controlled than for other methods (see Table 2 in ref 96). This may make cleaving a good choice for resolving the values of IFEs for different orientations of the solid in contact with the liquid. Its disadvantage is its complexity due to the presence of several different steps, each with its own setup.

The mold integration technique stands out for its simplicity in setting up an initial simulation box. One needs an initial liquid phase at the coexistence conditions at which the IFE must be evaluated. This phase should be a bulk phase if a single-component system is being studied. However, if two or more components are present and the solid is in equilibrium with more than one fluid phase (including vapor phases), as happens in hydrate systems, the initial simulation box must include the corresponding equilibrated fluid phases under the coexistence conditions. This technique does not need to create an equilibrium solid–liquid interface, as the method is based on the induction of the solid phase (from a fluid configuration) using a mold of attractive wells located at the crystallographic position of the solid phase. Because these positions are well-defined, the construction of the mold is a relatively easy task, even when dealing with complex solid structures of pure or binary mixture systems. Since the method involves a thermodynamic integration to evaluate the difference in free energy between the fluid system with and without the mold of attractive wells, it may be used in any available Monte Carlo or molecular dynamics codes. Another advantage is that the system size needed is relatively small compared to other more sophisticated techniques. However, a serious drawback is the need to find the optimal cutoff radius of the attractive wells of the mold. This is both necessary to obtain a reliable result and tricky as well as time-consuming in practice.

The Einstein crystal methodology avoids any explicit real-space transformation from a bulk system to one with an interface. This makes it a good choice for surface configurations that are not readily accessible by other methods (examples include stepped surfaces and surface patterning). It is also well-adapted to dealing with systems that involve complex electrostatics. This makes it the method of choice for systems where there is an interchange of species between the liquid and solid phases (as when solvent of crystallization is present in the solid phase). The Einstein crystal method can account for such complexities in a straightforward manner. It is also appropriate for cases where the bulk material has low solubility in a solvent. However, it requires the construction of reversible thermodynamic pathways. This can make it complex to execute in practice and requires significant computer resources. In highly soluble systems, or near the coexistence point, difficulties can arise in the correct assignment of atoms to the solid and liquid phases.

The phantom wall and dry surface methods both have the advantage that setting up the calculation is much simpler than for other methods. The starting point *already* contains a solid–liquid system in contact through an interface. Therefore, they do not need any special preparation beyond the setup of a “standard” simulation box. The calculation is obtained in a single step, namely, pushing away the liquid from the solid using transparent walls (in the phantom wall method) or switching off interactions between solid and liquid (in the dry surface method). The disadvantage is that the method requires an independent calculation of the IFE of the solid in contact with vacuum; otherwise only the work of adhesion can be determined. This is less important if one is interested in wetting properties of the liquid in contact with the solid (where the determination of the work of adhesion is enough) but it should be kept in mind if the solid–liquid IFE is the quantity sought after.

One important point to remember is that all these methodologies give the value of γ_*sl*_ for a single point. The calculation of γ_*sl*_ along the coexistence line would require to include other techniques, such as the Gibbs–Cahn integration method.

## Nomenclature

A: Total area of the interface
$A_{C}$: Surface area of the critical cluster
ϕ^+^: Attachment rate
⟨γ⟩: Interfacial free energy averaged over crystal orientations
*k*_*B*_: Boltzmann constant
*n*_*j*_: Component of the unit vector normal to the interface in direction *j*
δ_*ij*_: Kronecker delta
*E*: Internal energy
*S*: Entropy
ϵ: Unit of energy LJ potential
η: Entropy per unit area (Gibbs notation)
[·]: Excess quantity at the interface per unit of area
·^*XS*^: Excess quantity at the interface
IFE: Interfacial free energy
γ^0^: Interfacial free energy of a flat interface
γ_*C*_: Interfacial Free Energy of a cluster evaluated at the surface of tension
γ_*g*_: Interfacial free energy per interface atom
γ_*sl*_: Solid–liquid interfacial free energy
γ_*sm*_: Solid-melt interfacial free energy
γ_*sv*_: Solid–vapor interfacial free energy
γ_*sx*_: Solid-solution interfacial free energy
γ_*s*_: Solid-vacuum interfacial free energy
*G*: Gibbs free energy
*F*: Helmholtz free energy
$U_{n}$: Cleaving potential for step *n*
μ: Chemical potential
*c*: Number of chemical components
ϕ­(*r*): Short-range repulsive potential, see eq 30
Ψ: Kramer potential
R: Radius of a cluster
$R_{C}$: Critical radius of a cluster
ρ_*l*_: Number density particles in the liquid
ρ_*s*_: Number density particles in the solid
σ: Unit of distance LJ potential
*γ̅*: Interfacial stiffness
ψ_*ij*_: Stress in the direction *i*,*j*
ζ: Supersaturation
∂: Tolman length
**n̂**: Normal unit vector to the interface
V: Volume
$V_{C}$: Volume of the critical cluster
*a*_0_: Activity coefficient of a solute
*a*_sat_: Activity coefficient of a solute at saturation
*D*: Diffusion coefficient
*e*: Energy per unit area (Gibbs notation)
*f*_*ij*_: Interfacial stress in the directions *i*,*j*
*J*_CNT_: Nucleation rate in the context of classical nucleation theory
*J*_kin_: Kinetic factor of the nucleation rate
*J*_thd_: Thermodynamic factor nucleation rate
*N*_A_: Avogadro number
*N*_c_: Number of critical nuclei
*N*_*k*_: Number of chemical species *k*
*N*_crit_: Number of particles in the critical nucleus
*P*_N_: Stress normal to the interface
*P*_T_: Stress tangent to the interface
*T*: Temperature
*U*: Configurational energy
*u*_*ij*_: Strain in the direction *i*,*j*
*Z*: Zeldovich factor
CNT: Classical nucleation theory
γ: Interfacial free energy
MD: Molecular dynamics
TI: Thermodynamic integration
